# Development of two smart acoustic yam quality detection devices using a machine learning approach

**DOI:** 10.1016/j.heliyon.2023.e14567

**Published:** 2023-03-16

**Authors:** J. Audu, R.R. Dinrifo, A. Adegbenjo, S.P. Anyebe, A.F. Alonge

**Affiliations:** a Department of Agricultural and Environmental Engineering, Federal University of Agriculture Makurdi, Nigeria; b Department of Agricultural Engineering, Lagos State Polytechnic Ikorodu, Nigeria; c Department of Agricultural and Environmental Engineering, Obafemi Awolowo University, Ife, Nigeria; d Department.of Agricultural and Bio - Env. Engineering Technology, Federal Polytechnic Nassarawa, Nigeria; e Department of Agricultural & Food Engineering, University of Uyo, Nigeria

**Keywords:** Artificial intelligence, Discriminant analysis, Sound-generating technique, Acoustic, Classification

## Abstract

Quality detection has been a major problem in the agriculture and food industries. This operation is mostly done by a subjective sensory method which is prone to high error and food destruction. Therefore, there is a need to apply artificial intelligence using a machine learning approach. This study developed two intelligent acoustic yam quality detection and classification devices using two sound-generating techniques. The software (multi-wave frequency generator) sound-generating technique generated sound from a laptop to a speaker inside a detecting chamber. This sound passes through the yam and was received on the opposite side by a microphone, into another laptop for analysis using visual analyzer software. The impact sound-generating technique used sound generated from a gentle impact of the yam on a flat surface placed inside the detection chamber. The sound produced was picked up by a microphone into a laptop for analysis. Acoustic properties considered were amplitude, frequency, sound velocity, wavelength, period and sound intensity. Discriminant analysis algorithm only was used in this first stage of the study to prove the applicability of machine learning. Three qualities (good, diseased damaged and insect-damaged) of two yam varieties (white and yellow yam) were tested. The device's performance of white yam was 79% and 68.7%, yellow yam was 82.3% and 68.7% for the software sound generation-technique and surface impact sound-generating technique, respectively. The study shows that the software sound-generating technique performed better in terms of overall yam quality detection and also proves the applicability of machine learning.

## 1 Introduction

Yam tuber cultivated in South America, Oceania, the Caribbean, Asia, and West Africa belongs to a family called *Dioscoreaceae* [1]. [2] reported that there are about 800 species documented. Archeological pieces of evidence had shown that the origins of different species of yam had been traced to Africa, Asia and America [[2], [3], [4]]. [1] stated that it contains varying amounts (percentages) of carbohydrates, proteins and vitamins [2]. reported that a tube was estimated to contain an average of about 494 kJ (118 kcal) of energy. Currently, it is reported to be cultivated in about 61 countries with a global production of about 73 million metric tonnes [3,4]. This high production will impact world trade.

[5] reported that the United States, India, Belgium, Japan and Jamaica in 2019 were the global major exporters of yam. These countries exported a total of 50,593.4 tonnes valued at $ 81,321,244 million. The world's largest producer of yam was reported to be Nigeria, accounting for over 70 to 76% of the world's production (Yam Market Report. 2022). According to Yam Market Report (2022), despite this high production. The developing countries do not have a significant export record. This is due to capital-intensive involvement in procuring mechanical processing equipment by processors [4]. This necessitates the need for this study to develop a cheap and nondestructive and intelligent technique to determine yam quality.

One of the fastest ways to detect quality in an industry setting is by employing the knowledge of artificial intelligence through machine learning [6]. According to Ref. [7]; the ability to make any machine behave and think like a human to achieve or solve a particular task is known as artificial intelligence. To achieve this intelligence in a machine, then the machine needs to be trained. Machine learning is a process of manipulating computers through programming with mathematical algorithms from data to imitate the way human reasons and make decisions [[8], [9], [10], [11]]. In this study, an intelligent device was developed using a machine-learning algorithm to detect the quality of two species of yam using its acoustic properties. Though, only one machine learning algorithm was used in this study. The goal of this first stage of the project was to prove the applicability of the machine learning algorithm. After this initial study, more machine-learning algorithms will be used to evaluate the devices further. The use of acoustic properties of a material to detect material changes has been in existence since the 1800s.

According to Refs. [12,13]; the use of acoustic properties of materials in research starts in the US in the 1830s. Though, these researches were not on food materials. Ref. [13] stated that research on the acoustic properties of food begins in the 1920s. Many researchers had used the acoustic properties of crops to successfully detect moisture, defects, insect infestations and other activities affecting harvested crops [14]. This study reviewed the general application of acoustic properties within the audible frequency, which had been applied to biomaterials to detect changes in their quality. This was done due to little or scarcity of information on the application of audible sound range being used in detecting post-harvest qualities of yam tubers. So, this review was done based on studies of different researchers on different crops [15]. used sound pressure levels of dropped wheat grains to detect their moisture contents with an accuracy of 90% [[16], [17], [18], [19]]. and [38], used the acoustic properties of apples to automatically detect their quality with an accuracy range of 80–100% [20]. carried out a study to detect the quality of apples and tomatoes and achieved an accuracy of 80% [21,22]. used the acoustic properties of wheat grains to automatically detect their quality and achieved an accuracy range of 92–95% [23,24]. used audible sound properties to detect the quality of coconut fruits and achieved an accuracy range of 83–97% [25]. use sound to detect beans pod and seeds qualities and achieved an accuracy range of 80–90% [26]. detects the crispness of a carrot with its acoustic properties at audible range frequency and achieved an accuracy of 70%. Refs. [27,28] used the acoustic vibration properties of pear to automatically detect its quality and achieved an accuracy of 85–93%, respectively. Ref. [29] developed an intelligent monitoring system using CO_2_ bust sound produced by white grubs (larvae of scarab beetles) that attach maize and soybeans seeds. Detection results are transmitted with GSM (Global System for mobiles) and GPS (Global positioning system) to the farmer's phone. Refs. [30,31] used audible sound frequency range to detect the ripeness and texture of watermelon and strawberry and achieved an accuracy of 92 and 99%, respectively. The acoustic properties of yam tubers have never been used to detect their quality. Therefore, this study is aimed at finding if the acoustic property can be used to correctly detect yam quality using machine learning. Hence, the objective of this study is to develop two smart devices that employed two different sound-detecting techniques using the acoustic properties of yam tubers to detect their quality.

## 2 Materials and methods

### 2.1 Design and construction of two acoustic yam quality detection devices

The two techniques used for developing the two yam quality devices were based on sound generation. These two sound generation techniques were:

i. Software sound generation technique
ii. Surface impact sound generation technique

#### 2.1.1 Design of the yam detecting devices

The design information and calculations of the two intelligent yam quality detectors were displayed in Tables S1 and S2 displayed in the supplementary document provided. These design values were used for the construction of the two detecting devices.

#### 2.1.2 Construction of the yam detecting devices

##### 2.1.2.1 Software sound generation-technique device

**Fig. 1:**
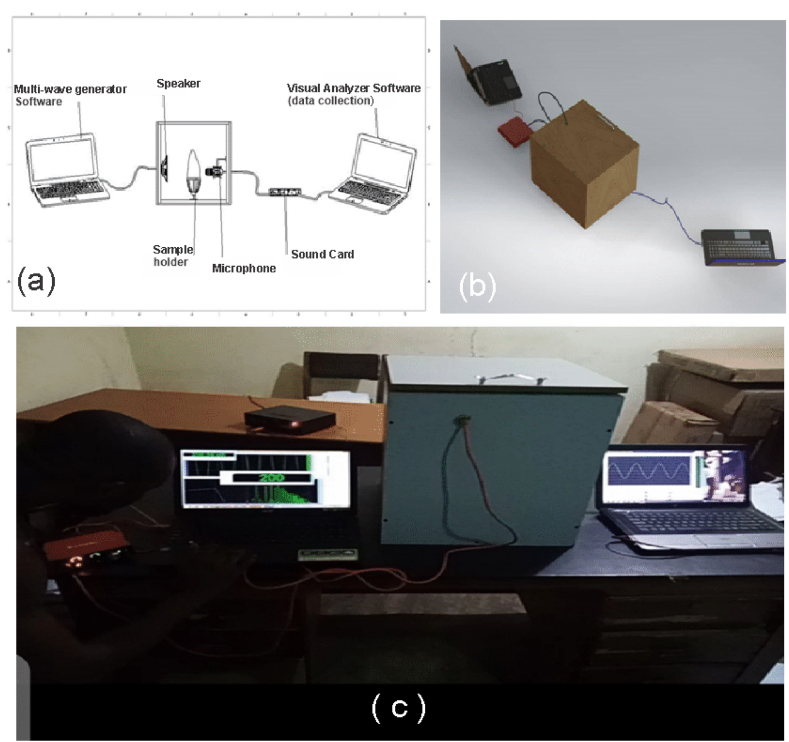
Software sound-generating technique yam quality detecting device (a) Conceptual labeled diagram (b) Conceptual 3D diagram (c) Actual Constructed device.

A 459 mm × 464 mm × 397 mm double-layer Mild density fiberboard (MDF) was used to construct the body of the detection chamber [35] as shown in Fig. 1. The inside of the detection chamber was covered with a 12.5 mm thick Styrofoam for soundproofing. Attached inside the chamber was 25 W, 4 Ω subwoofer speaker to one end of the chamber. A multi-function Condenser Microphone having a 3.5 mm Jack for computer input was attached at the opposite end of the speaker in the chamber. A sample holder of dimension 192 mm × 140.5 mm was placed between the speaker and the microphone. The speaker was then connected to a Hp laptop installed with software called multi-wave frequency generator version 1.2 for sound generation. The microphone was then connected to a Focusrite Solo Soundcard which in turn was connected to a Vibes Cor VC AMP 12 BT Home Amplifier. The Amplifier was then connected to a Hp laptop installed with software called visual analyzer 2011 for sound analysis and collection of data.

##### 2.1.2.2 Sample impact sound generation-technique device

**Fig. 2:**
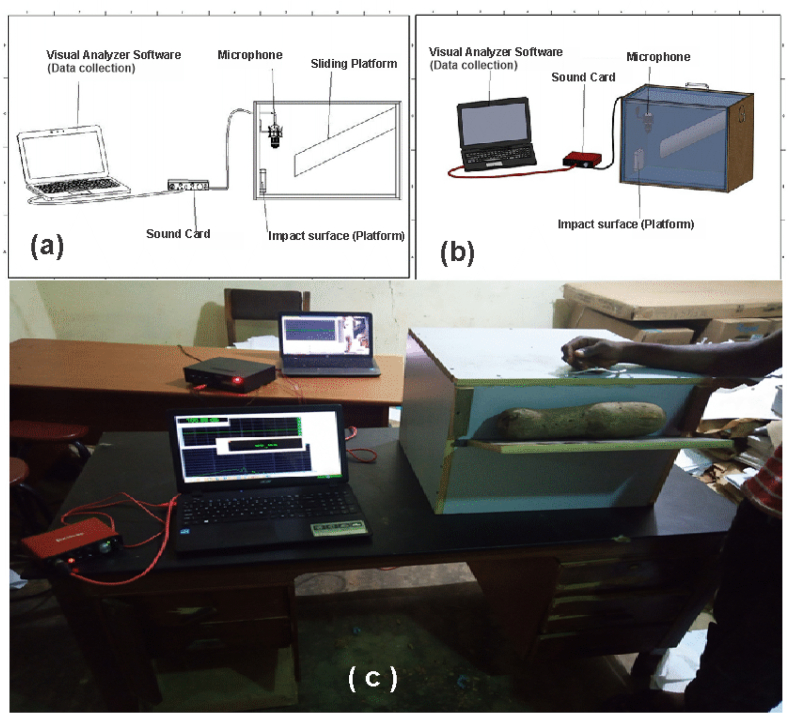
Sample impact sound generation-technique yam quality detecting device (a) Conceptual labeled diagram (b) Conceptual 3D labeled diagram (c) Actual Constructed device.

A 382.8 mm × 522 mm × 478.42 mm double-layer Mild density fiberboard (MDF) was used to construct the body of the detection chamber [35] as shown in Fig. 2. The inside of the detection chamber was covered with a 12.5 mm thick Styrofoam for soundproofing. The detecting chamber consists of a sliding panel with an impact surface directly opposite it. Above the impact surface, was attached a multi-function condenser microphone having a 3.5 mm Jack for the computer input. The microphone was then connected to a Focusrite Solo Soundcard which in turn was connected to a Vibes Cor VC AMP 12 BT Home Amplifier. The Amplifier was then connected to a Hp laptop installed with software called visual analyzer 2011 for sound analysis and collection of data.

### 2.2 Evaluation of the developed yam quality detection devices

#### 2.2.1 Sample acquisition and preparation

Six hundred tubers consisting of 300 white yams (*Dioscorea rotundata*) and 300 yellow yams (*Dioscorea cayanensis*) were acquired from National Root Crops Research Institute, Umudike, Nigeria.

**Fig. 3:**
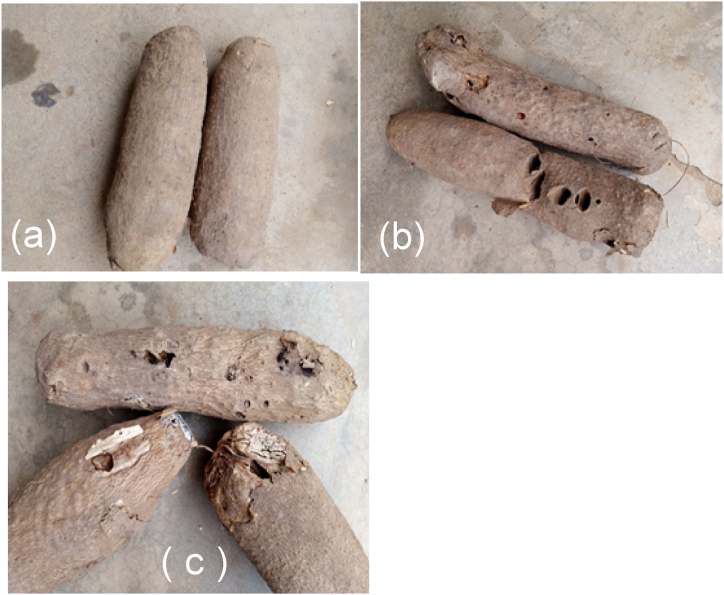
Typical yam quality samples (a) Good yams (b) Insect damage yams (c) diseased damage yams.

These acquired tubers were taken to the agronomy laboratory at the federal university of Agriculture, Makurdi, Nigeria for inspection and verification. The quality of yam tubers acquired was one hundred (100) Good yams, one hundred (100) diseased yams and one hundred (100) insect-destroyed yams of white yams and yellow yams, respectively. Typical yam quality samples are displayed in Fig. 3.

#### 2.2.2 Experimental procedures for acoustic data acquisition

##### 2.2.2.1 Software sound generation-technique device

**Fig. 4:**
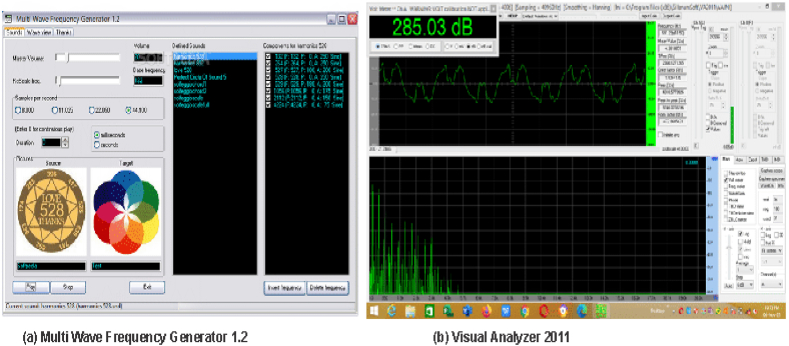
Typical screenshots of software readings used for (a) sound generation and (b) sound analyzing.

The Yam sample was placed on the sample holder and the detection chamber was closed, to make the chamber airtight. An audible sound wave of 200 Hz was generated using the multi-wave frequency generator version 1.2 installed on the laptop computer. This value (200 Hz) was used because it was recommended by the [37] to be within the safe hearing frequency range. This sound wave was passed into the detecting chamber through the speaker. The sound passes through the yam sample and is then collected by the microphone into the sound card. The sound card then passes the sound wave to the amplifier which in turn passes it into the laptop computer into the visual analyzer 2011 software. Typical screen displays of this sound-generating and analyzing software were shown in Fig. 4a and b, respectively. Then acoustic properties of the sound waves like amplitude, frequency, velocity, wavelength, period and intensity were recorded for each yam quality sample examined. Recorded data are taken for statistical analysis using a machine learning algorithm called multiple discriminant analysis. The reason for using only one machine learning algorithm and only six acoustic properties at this first stage of the project was, to prove the applicability of the machine learning approach, using acoustic properties of yam quality. When this approach has been proven, more machine-learning algorithms and acoustic properties will be used in a future evaluation study to improve detection and classification accuracy.

##### 2.2.2.2 Sample impact sound generation-technique device

The Yam sample was placed at the top of the sliding platform and then allows sliding until it hit the impact platform. Then, the sound wave produced by this impact was received by the microphone into the sound card. The sound card then passes the sound wave to the amplifier which in turn passes it into the laptop computer into the visual analyzer 2011 software. A typical reading display of this software was shown in Fig. 4b. Then acoustic properties of the sound waves like amplitude, frequency, velocity, wavelength, period and intensity were recorded for each yam quality sample examined. Recorded data are taken for statistical analysis using a machine learning algorithm called multiple discriminant analysis.

### 2.3 Data analysis

Acoustic data collected from the two detecting devices were analyzed using SPSS software version 23. A machine learning algorithm called multiple linear discriminant analysis was used to correctly classify yam quality categories. Leave-one-out cross-validation analysis was used to validate the classification results obtained.

#### 2.3.1 Discriminate analysis

The formula used by the discriminant algorithm for classification was displayed as equation (1) [32,33],

(1) $$P\left(C_{i}|DSF\right)=\left[\frac{\left[P\left(DSF|C_{i}\right)P\left(C_{i}\right)\right]}{\sum P\left(DSF|C_{i}\right)P\left(C_{i}\right)}\right]$$

Where:

$P\left(C_{i}|DSF\right)$ = Posterior probability that a case was in yam quality class i, given that it has a specific discriminant score DSF
DSF = Discriminant Score for Function (Model)
$\left(DSF|C_{i}\right)$ = Conditional probability that a case has a discriminant score of DSF, given that it was in yam quality class i
$P\left(C_{i}\right)$ = prior probability that a case was in group i, which would be equal to (n_i_/N)
N = Total number of samples
n_i_ = Number of cases correctly classified

#### 2.3.2 Classification and validation

The classification was done using the confusion matrix table. There were no hold-out samples before classification. Leave one cross-validation method was used to validate the developed classification models by reclassifying the samples by leaving one case out. To achieve cross-validation by predicting an output (Y) at point (X), given input data in matrix X_2_ and output Y_2_, equation (2) was used [32,33]:

(2) $$output\;\left(Y\right)=\hat{\mu }+R\left(X,X_{2}\right)R{\left(X_{2}\right)}^{-1}\left(Y_{2}-\mu 1_{n}\right)$$

Where:

$\hat{\mu }$ = overall mean, R = correlation matrix, μ = mean of samples under consideration, n = number of observations under consideration.

So, for leave-one-out predictions, the matrix X_2_ will have all the design points except for the one we are predicting.

## 3 Results and discussion

### 3.1 White yam *(Discorea rotundata)*

Results of the entire discriminant analysis for detection and evaluation of white yam, for the developed devices on software generation-technique and surface impact generation-technique, are displayed in Tables S3 and S4 (tables too large for manuscript) submitted as a supplementary document. These tables also show predictions for white yam quality by the discriminant software using the discriminant score functions (equations). These score functions (equations) were used to determine the probability that the predicted quality belongs to either quality class 1 (good), class 2 (diseased damaged), or class 3 (insect-damaged).

Table 1 shows the mean and standard deviation of acoustic properties obtained during the evaluation analyses of white yam quality for the developed devices, using both the software and sound generation techniques. These results show that the acoustic properties of good white yams were higher in values for the software sound generation technique than for the surface impact sound generation technique. This phenomenon can be attributed to the sound generation frequency of the software sound generation technique being constant; while that of the surface impact sound generation technique was not fixed but depended on sample impact. Looking at the statistical behavior of the obtained results, the acoustic property having the highest deviation from the mean for both techniques is the intensity of the sound. This is because according to Ref. [14]; the intensity of the sound measured through solid material depends on the area of the sample that sound must travel through. So, since these white yam samples were not of the same size, the amount of sound going through them will defer notwithstanding the technique used. Also, similar behavior was observed with potatoes, by Ref. [34] using sound impact technique only, to detect and classify using discriminant analysis.

**Table 1:** Descriptive statistic on the performance of two acoustic techniques used in detecting white yam quality.

White Yam			Software Sound generation technique		Surface impact sound generation technique	
Yam Quality	Acoustic Property	N	Mean	Std. Deviation	Mean	Std. Deviation
Good	Amplitude (db)	100	270.621	14.125	147.573	27.765
	Frequency (Hz)	100	200.000	0.000	155.714	11.506
	Intensity (db)	100	4333.156	21906	1448	247
	Period (s)	100	5.000	0.000	2.320	0.490
	Velocity (m/s)	100	25.000	0.000	16.000	0.000
	Wavelength (m)	100	0.125	0.000	0.101	0.013
Disease damaged	Amplitude (db)	100	307.764	8.240	72.907	9.309
	Frequency (Hz)	100	200.000	0.000	83.540	9.446
	Intensity (db)	100	2462	65.916	800.876	314.904
	Period (s)	100	5.000	0.000	1.010	0.100
	Velocity (m/s)	100	25.000	0.000	8.000	0.000
	Wavelength (m)	100	0.125	0.000	0.094	0.012
Insect Damaged	Amplitude (db)	100	285.566	11.599	143.055	9.576
	Frequency (Hz)	100	200.000	0.000	153.772	9.415
	Intensity (db)	100	2290	92.681	1369	225.561
	Period (s)	100	5.000	0.000	2.240	0.429
	Velocity (m/s)	100	25.000	0.000	16.000	0.000
	Wavelength (m)	100	0.125	0.000	0.102	0.009
Total	Amplitude (db)	300	287.984	19.150	121.178	38.558
	Frequency (Hz)	300	200.000	0.000	131.009	35.125
	Intensity (db)	300	3028	12639	1206	391.775
	Period (s)	300	5.000	0.000	1.857	0.710
	Velocity (m/s)	300	25.000	0.000	13.333	3.778
	Wavelength (m)	300	0.125	0.000	0.099	0.012

N is the experimental observation number.

The mean and standard deviation values of acoustic properties for diseased damaged white yams using the software and impact sound generation techniques show higher values for the software technique than for the impact technique. The same explanation given for good white yam can also be used to explain this phenomenon also. The acoustic property having the highest deviations from the mean for both techniques is the intensity of the sound. Again, the same explanation given for good white yams can also be used to explain this phenomenon in diseased damaged yams.

The insect-damaged result mean and standard deviation values of acoustic properties for software and impact sound generation techniques, also follow the same pattern. The same explanation given for white good yams is applicable here as well. The descriptive statistic alone can not give us an in-depth explanation of how these acoustic properties affect the choice for detecting these white yam qualities. So, we take a look at the test for equality of variance, group means and covariance matrices for the two acoustic techniques.

The test for equality of variance, group means and covariance matrices are statistical properties that show if the acoustic data obtained can be discriminated against using discriminant analysis. These tests are displayed in Table S5 submitted in the supplementary document. The first test on the table is the test of equality of yam quality groups means. This test is a univariate ANOVA test, using ‘Wilks' Lambda’ and ‘Fisher (F)’ values. Lower wilks' lambda and F values mean that the variable had effects on the detection classification. In the software sound generation technique, the test shows that among the five acoustic properties tested only amplitude and Intensity can be tested while the remaining variables (frequency, period, velocity and wavelength) can not be tested because they do not show any variation among its data sets. Now, between amplitude and intensity that were tested, only amplitude was found to have an effect while intensity does not have any effect on detecting white yam quality. This decision was taken because, the amplitude has a low wilks' lambda value of 0.363 and f value of 4.24E-66, while Intensity has a higher wilks' lambda value of 0.995 and f value of 4.49E-01. This means that in using the software sound generation technique only one acoustic property or variable (amplitude) is required. Therefore, this makes the software technique less expensive and an easy acoustic form of detecting yam quality.

In the surface impact sound generation technique, the test of equality shows that among the five acoustic properties tested, variables like amplitude, frequency, period, wavelength and Intensity can be tested; while only sound velocity can not be tested because velocity data do not have many variations. In this impact technique, it was found that: amplitude, frequency, intensity, period and wavelength were affecting the detection of white yam quality. The decision was taken because their wilks’ lambda values were higher while their F values were lower. This means that the surface impact sound generation technique requires five acoustic properties (variables) to detect white yam quality, unlike the software sound generation technique that requires just one.

Another statistical test conducted was the box's tests of equality of covariance matrices. It tests the homogeneity of variances and covariances within the white yam quality groups. So, to show that the covariance matrices are the same for data of all-white yam quality groups the log determinant was found. The equal log determinant shows equal covariance matrices among the quality groups. Log determinants for good, diseased damage and insect damage in the software sound generation technique were not equal. Therefore, violating the assumption that the quality group covariance matrices must be equal. Nevertheless, ‘Box M; value can still be calculated to see if it has an effect. Non-significant 'Box M; value with an unequal log determinant still proves that the quality group covariance matrices are equal, this situation always occurs when data are large. The ‘Box M value calculated was 2956.271 with an F value of 487.918. This also violates the assumption that the quality group covariance matrices are equal but the analysis can continue because these can also occur when data are large. Log determinants for good, diseased damage and insect damage in the surface impact sound generation technique were also not equal. The calculated ‘Box M value was 1244 with an F value of 40.497. This also violates the assumption that the quality group covariance matrices are equal but the analysis can continue because these can also occur when data are large. Despite the drawback of the 'Box M' test, the test of the discriminant score functions will give a better insight into the discriminant analysis.

A summary of canonical discriminant score functions of white yam for both acoustic techniques is shown in Table S6 (submitted in the supplementary document). The discriminant score functions are equations that will be used to determine the probability of a case belonging to a quality group. In this analysis, two discriminant score functions (equations) were developed for the software sound-generation technique and another two for the surface impact sound-generating technique. In the software sound generation technique, the first discriminant score functions (equations) developed had a higher Eigenvalue, % of the variance, cumulative % and canonical correlation than the second function (equation). The eigenvalue is the ratio between and within quality groups sum of squares and a higher value makes a discriminant score function (equation) a better predictor. So, for the software sound generation technique, the first discriminant score function (equation (3)) was chosen because it has a higher eigenvalue and higher canonical correlation value than the second function (equation (4)). These equations (functions) are also displayed in Table S6 (submitted in the supplementary document). In the surface impact sound-generating technique the first discriminant score functions (equations) developed had higher Eigenvalue, % of the variance, cumulative % and canonical correlation than the second discriminant score functions (equations). So, for the impact sound-generating technique the first discriminant score function (equation (5)) was chosen because it has a higher eigenvalue and higher canonical correlation value than the second function (equation (6)). These equations (functions) are also displayed in Table S6 (submitted in the supplementary document).

Wilks’ lambda test was also done to the discriminant score functions (equations) for both techniques. The test shows that for the software sound generation technique, the wilks' lambda value, chi-square value, degree of freedom and P-value for the first developed discriminant score functions (equations) were better than the second. The first discriminant score function (equation (3)) was again chosen over the second because it has a lower wilks' lambda value. Also, this same wilks’ lambda test for the surface impact sound-generating technique shows the wilks' lambda value, chi-square value, degree of freedom and P-value for the first developed discriminant score functions (equations) were better than the second. The first discriminant score function (equation (5)) was again chosen over the second (equation (6)) because it has a lower wilks' lambda value. Another test called the standardized discriminant score function coefficients test” was done for functions (equations) developed for both techniques. This test shows the importance of the coefficients to the developed functions (equations). In the software sound generation technique, only the amplitude and intensity of sound variables were considered. The choices of these two variables were the same as explained for the test of equality of variance. The importance of the values of the coefficients for amplitude (db) and intensity (db) was displaced in Table S6 (submitted in the supplementary document) for the first and second discriminant score functions (equations (3), (4))) developed. These values show that only the coefficient of amplitude and intensity in the first developed function (equation (3)), was important to it. In the surface impact sound-generating technique only the amplitude, frequency, intensity, period and wavelength variables were considered. The choices of these five variables were the same as explained for the test of equality of variance Their importance of the values of the coefficients for amplitude (db), frequency (Hz), intensity (db), period (s) and wavelength (m) were displaced in Table S6 (submitted in the supplementary document) for the first and second discriminant score function (equations (4), (5))) developed. These values show that for the first developed function (equation (5)), only the coefficient of the period has negative importance while in the second discriminant score function (equation (6)) developed, only the coefficient of frequency has negative importance (see Table S6 submitted in the supplementary document). These phenomena can be attributed to the difference in weights of the different yam samples used for the surface impact experiments. Also, a further statistical test was carried out on the functions (equations) developed. This further test was called the “structure matrix test” and was carried out for the function developed for both techniques. The structure matrix test tells the correction between the acoustic properties variable and the developed functions (equations). In the software sound generation technique, only the intensity and amplitude of sound variables were considered as explained by the equality of variance test, for the two developed functions (equations (3), (4))). The values of intensity (db) and amplitude (db) for the test obtained were - were displaced in Table S6 (submitted in the supplementary document) for the two developed functions (equations). This again shows that the intensity of the sound did not contribute to the prediction of yam quality. This phenomenon was caused by using a constant frequency of sound. In the surface impact, the sound-generating technique only the amplitude, frequency, intensity, period and wavelength variables were considered as explained by the equality of variance test, for the two developed functions (equations (5), (6))). The values of amplitude (db), frequency (Hz), intensity (db), period (s) and wavelength (m) for the test obtained were displaced in Table S6 (submitted in the supplementary document) for the two developed functions (equations (5), (6))). This again shows the superiority of the first function (equation (5)) over the second function (equation (6)). The developed discriminant score functions (equations) for both techniques are displayed from equations (3)–(6) (also see Table S6 in the supplementary document) [23,24]. observed similar discriminant pattern in their study of coconut quality detection and classification.

### 3.2 Software sound generation technique

(3) DSF1=0.086A−6.149I−24.855

(4) DSF2=0.004A+7.888E−5I−1.284

### 3.3 Surface impact sound generation technique

(5) DSF1=0.002A+0.130F+3.55E−4I−0.273T+82.855W−25.374

(6) DSF2=0.015A−0.081F+0.004I+1.425T+32.153W−2.287

Where.

DSF1 is the discriminant score function (equation) for the first developed function (equation).

DSF2 is the discriminant score function (equation) for the second developed function (equation).

A = amplitude (db), F = frequency (Hz), I = intensity (db), T = period (s) and W = wavelength (m)

The first discriminant score function (equations (3), (5))) was chosen in either technique for white yam quality detection classification.

The detection classification or confusion matrix results for the discriminant analysis of white yam quality, for both acoustic techniques used in the study are displayed in Table 2. This table shows that two experimental classifications were carried out for both acoustic techniques used in the study. These detection classifications were called original classification and validated classification. In the software sound generation technique, the actual detection classification result shows that out of the 100 good white yams used for the detection. A total of 69% of them were correctly detected and classified as good yams, 31% were wrongly detected and classified as insect damage yams while no good yams were wrongly classified as diseased damaged yams. Still on the original classification, out of the 100 diseased damaged white yams used for the detection. 97% were correctly detected and classified as diseased damaged yams while 1% were wrongly detected and classified as good yams and 2% were wrongly detected and classified as insect-damaged yams. Again, still on the original classification, out of the 100 insect-damaged white yams used for the detection. A total of 73% were correctly detected and classified as insect-damaged yams. While 19% were wrongly detected and classified as good yams and 8% were wrongly detected and classified as diseased damaged yams. The overall original performance of correctly detecting the classification of all-white yam quality considered in this study using the software sound generation technique for the original detection classification experiment was 79.7%. A cross-validation detection classification experiment was done again to confirm the original result. In the cross-validation detection and classifications done, out of the 100 good white yams used for the detection and classifications. A total of 68% of them were correctly detected and classified as good yams, 31% were wrongly detected and classified as insect damage yams and 1% were wrongly classified as diseased damaged yams. Still on the validation classification, out of the 100 diseased damaged white yams used for the detection. A total of 97% were correctly detected and classified as diseased damaged yams. In comparison, 1% were wrongly detected and classified as good yams and 2% were wrongly detected and classified as insect-damaged yams. Again, still on the validation classification, out of the 100 insect-damaged white yams used for the detection. A total of 73% were correctly detected and classified as insect-damaged yams. In comparison, 19% were wrongly detected and classified as good yams and 9% were wrongly detected and classified as diseased damaged yams. The overall cross-validated performance of correctly detecting all-white yam quality considered in this study using the software sound generation technique was 79%. In the surface impact sound-generating technique, the actual detection classification result shows that out of the 100 good white yams used for the detection. A total of 37% of them were correctly detected and classified as good yams, 62% were wrongly detected and classified as insect damage yams and 1% were wrongly classified as diseased damaged yams. Still on the original classification, out of the 100 diseased damaged white yams used for the detection. A total of 100% was correctly detected and classified as diseased damaged yams while none was wrongly detected and classified as neither good yams nor insect-damaged yams. Again, still on the original classification, of the 100 insect-damaged white yams used for the detection. 74% were correctly detected and classified as insect-damaged yams while 26% were wrongly detected and classified as good yams and none were wrongly detected and classified as diseased damaged yams. The overall original performance classification of correct detections of all-white yam quality considered in this study using the surface impact sound-generating technique was 70.3%. Cross-validation detection was done again to confirm the original result. In the cross-validation detection and classifications done, out of the 100 good white yams used for the detection and classifications. A total of 35% were correctly detected and classified as good yams, 64% were wrongly detected and classified as insect damage yams and 1% were wrongly classified as diseased damaged yams. Still on the cross-validation classification, out of the 100 diseased damaged white yams used for the detection. A total of 100% was correctly detected and classified as diseased damaged yams while none was wrongly detected and classified as neither good yams nor insect-damaged yams. Again, still on the cross-validation classification, out of the 100 insect-damaged white yams used for the detection. A total of 71% were correctly detected and classified as insect-damaged yams. In comparison, 29% were wrongly detected and classified as good yams and none were wrongly detected and classified as diseased damaged yams. The overall cross-validated performance of correct detections of all-white yam quality considered in this study using the surface impact sound-generating technique was 68.7%. Comparing both acoustic techniques, the software sound generation technique detected more good yams while the surface impact sound-generating technique detects more diseased damaged yams. Both acoustic techniques detect insect-damaged yams equally well. The reason for the high detection of good yams by the software sound generation technique could be due to the lack of interference with the transmission of a sound wave through the yam tubers. Also, the uniform texture of the sound transmission material can account for the high detection of good yams. The poor detection of diseased and insect damage in the sound generation technique could be due to differences in texture within the yam tubers. These differences in texture hinder the smooth transmission of sound through the yam tubers. The reason for the high detection of diseased yams than good yams in the surface impact sound-generating technique could be attributed to the difference in yam tuber tissue texture (deformation) as well. Tissue deformation could have softened the yam tubers; therefore, making them produce low-frequency sounds upon impact with any solid surface than good yam tubers. Though, the overall performance of white yam quality detection was higher in the software sound generation technique. The surface impact sound-generating technique is the best to use when the goal of the detection was to detect diseased damaged white yams. The acoustic detection of white yam tubers quality results achieved was better than those achieved by Ref. [26] for the detection of the crispness of a carrot. Also, the detection results obtained for white yam tubers fall within the ranges obtained by Refs. [23,24] for coconut quality detection, and below that obtained by Refs. [30,31] for the ripeness and texture of watermelon and strawberry.

**Table 2:** Classification or confusion matrix results for the discriminant analysis detection of white yam quality for both acoustic techniques.

Yam quality			Software Sound generation technique				Surface Impact sound-generating technique			
			Predicted Group Membership			Total	Predicted Group Membership			Total
			Good	Disease damaged	Insect Damaged		Good	Disease damaged	Insect Damaged	
Original	Count	Good	69	0	31	100	37	1	62	100
		Disease damaged	1	97	2	100	0	100	0	100
		Insect Damaged	19	8	73	100	26	0	74	100
	%	Good	69	0	31	100	37	1	62	100
		Disease damaged	1	97	2	100	0	100	0	100
		Insect Damaged	19	8	73	100	26	0	74	100
Cross-validated	Count	Good	68	1	31	100	35	1	64	100
		Disease damaged	1	97	2	100	0	100	0	100
		Insect Damaged	19	9	72	100	29	0	71	100
	%	Good	68	1	31	100	35	1	64	100
		Disease damaged	1	97	2	100	0	100	0	100
		Insect Damaged	19	9	72	100	29	0	71	100
Overall White yam quality detection			**79.7%** of original cases were correctly classified.				**70.3%** of original cases were correctly classified.			
			**79.0%** of cross-validated cases were correctly classified.				**68.7%** of cross-validated cases were correctly classified.			

White yam should be used as a game changer in the quest for global food security, due to its high calorific content per kg when compared to other crops. Therefore, there is a need to increase its export technological capability. This study developed a very cheap method to detect white yam quality for large export activity by just using its acoustic properties. These two developed techniques can be used to design different types of yam quality detection and separation equipment for yam exportation. The limitation of these developed techniques is that the user is expected to possess basic acoustic and computer knowledge..

### 3.4 Yellow yam (*Dioscorea cayanensis)*

Results of discriminant analysis for detection evaluation of yellow yam for the developed devices for software generation-technique and surface impact generation-technique are displayed in Tables S7 and S8 (tables too large for manuscript) submitted as a supplementary document. These tables also show predicted yellow yam quality by the discriminant software using either the first or second discriminant score function (equation). The discriminant score function then was used to determine the probability that the predicted quality belongs to either quality class 1 (good), class 2 (diseased damaged), or class 3 (insect-damaged). Table 3 shows the mean and standard deviation of acoustic properties obtained during the evaluation of yellow yam for the developed devices. These results show that the acoustic properties of good yellow yams were higher in values for the software sound generation-technique than for the surface impact sound generation technique. This phenomenon can be attributed to the sound generation frequency of the software sound generation-technique being fixed; while that of the surface impact sound generation-technique was not fixed but was based on sample impact. The acoustic property having the highest deviations from the mean for both techniques is the intensity of the sound. This is because the intensity of the sound measured depends on the area of the sample. So, since these yellow yam samples were not of the same size, the amount of sound going through them will defer notwithstanding the technique used. Also, similar behavior was observed with potatoes, by Ref. [34] using sound impact technique only, to detect and classify using discriminant analysis.

**Table 3:** Descriptive statistic on the performance of two acoustic techniques used in detecting yellow yam quality.

Yellow Yam			Software Sound generation technique		Surface impact sound generation technique	
Yam Quality	Acoustic Property	N	Mean	Std. Deviation	Mean	Std. DeviationStd. Deviation
Good Yam	Amplitude (db)	100	2907	26188.	318.391	1726
	Frequency (Hz)	100	150.830	7.841	0.006	0.001
	Velocity (v/s)	100	125666	47947	166.780	39.061
	Wavelength (m)	100	49839	213523	1045	245.458
	Period (s)	100	0.005	0.000	175420	101794
	Intensity (db)	100	56447	30494	45308	221269
Insect damaged Yam	Amplitude (db)	100	3429	25953	134.399	22.364
	Frequency (Hz)	100	156.480	9.215	0.006	0.001
	Velocity (v/m)	100	1067	951.832	151.810	36.975
	Wavelength (m)	100	152997	22924	5311	21512
	Period (s)	100	0.005	0.000	153308	76012
	Intensity (db)	100	486381	1808198	18469	6472
Diseased damaged Yam	Amplitude (db)	100	296.266	17.016	1664.229	15287.966
	Frequency (Hz)	100	175.677	19.820	0.014	0.014
	Velocity (m/s)	100	12912	117991	87.880	15.844
	Wavelength (m)	100	208022	164412	4933.341	43863.637
	Period (s)	100	0.005	0.000	146105.648	649042.884
	Intensity (db)	100	178253	934925	18523.229	4896.031
Total	Amplitude (db)	300	2211	21260	705.673	8879
	Frequency (Hz)	300	160.996	17.088	0.009	0.009
	Velocity (m/s)	300	46549	92383	135.490	47.070
	Wavelength (m)	300	136953	168918	3763	28178
	Period (s)	300	0.005	0.000	158277	380762
	Intensity (db)	300	240360	1185384	27433	128035

The mean and standard deviation values of acoustic properties like amplitude (db), frequency (Hz), velocity (m/s), wavelength (m), period (s), and intensity (db) were displayed in Table 3 for diseased damaged yams using the software sound generation technique. Similarly displayed were values obtained for diseased damaged yams using the surface impact sound generation technique. These results show that the acoustic properties of diseased damaged yellow yams were higher for the software sound generation technique than for the surface impact sound generation technique. The same explanation given for good yellow yam can also be used to explain this phenomenon. The acoustic property having the highest deviations from the mean for both techniques was the intensity of the sound for diseased damaged yellow yams. Again, the same explanation given for good yellow yam can also be used to explain this phenomenon. The mean and standard deviation values of acoustic properties like amplitude (db), frequency (Hz), velocity (m/s), wavelength (m), period (s), and intensity (db) were also, given in Table 3 for insect-damaged yellow yams using the software sound generation technique. Similarly displayed were values obtained for insect-damaged yellow yams using the surface impact sound generation technique. The same explanation given for good yellow yams is applicable here. The descriptive statistic can not give us an in-depth explanation of how these acoustic properties affect the choice for detecting these yellow yam qualities. So, we take a look at the test for equality of variance, group means and covariance matrices for the two acoustic techniques.

The test for equality of variance, group means and covariance matrices are statistical properties that show if the acoustic data obtained can be discriminated against using discriminant analysis. These tests are displayed in Table S9 for yellow yam (table too large for manuscript) submitted as a supplementary document. The first test on the table is the test of equality of yam quality groups' means of yellow yam. This test is a univariate ANOVA test, using ‘Wilks' Lambda’ and ‘Fisher (F)’ values. Lower wilks' lambda value and F value means that the variable is affecting the detection. In the software sound generation technique for yellow yam, the test shows that all six acoustic properties can be tested, unlike the white yam test. So, for all the six acoustic properties tested frequency, intensity, period and wavelength were found to be affecting the detection with low wilks' lambda values. This means that only these four acoustic properties were used to decide the quality of yellow yam. This phenomenon occurs because unlike the white yam the frequency of the sound generated was not constant but varied slightly. This variation was responsible for more sound properties being involved in the determination of yellow quality. Acoustic properties like amplitude and velocity were found to not have any effect on the detection with high wilks' lambda values. So, these two acoustic properties do not play any part in deciding the quality properties of yellow yam. In the surface impact sound generation technique, the test shows that among the six acoustic properties tested, variables like amplitude, frequency, intensity and wavelength were found to be affecting the detection with low wilks' lambda values; while velocity and period were found to not affect the detection with high wilks' lambda values. This means that only acoustic properties like amplitude, frequency, intensity and wavelength are important in deciding the quality of yellow yam using the surface impact sound generation technique while velocity and period were not. This could be attributed to the low sound produced by the yam impacts on the surface. The box's tests of equality of covariance matrices test the homogeneity of variances and covariances within the yellow yam quality groups. So, to show that the covariance matrices are the same for data of all yellow yam quality groups the log determinant was found. The equal log determinant shows equal covariance matrices among the quality groups. Log determinants for good, diseased damage and insect-damaged in the software sound generation technique were 90.789, 86.235 and 86.494. These values are not equal therefore violating the assumption that the quality group covariance matrices must be equal. Nevertheless, ‘Box M; value can still be calculated to see if it has an effect. A non-significant 'Box M; value with an unequal log determinant still proves that the quality group covariance matrices are equal, this situation always occurs when data are large. ‘Box M value was 3425 with an F value of 111.43. This also violates the assumption that the quality group covariance matrices are equal but the analysis can continue because these can also occur when data are large. Log determinant values for good, diseased damage and insect damage in the surface impact sound generation technique with its ‘Box M and F value; also violates the assumption that the quality group covariance matrices are equal but the analysis can continue because these can also occur when data are large. Despite this drawback with the ‘Box M’ test, the test of the discriminant score functions will give a better insight into the discriminant analysis.

A summary of canonical discriminant score functions of yellow yam for both acoustic techniques is shown in Table S10 (table too large for manuscript) and submitted as a supplementary document. The discriminant score functions (equations) are equations that will be used to determine the probability of a case belonging to a quality group. In this analysis, two discriminant score functions (equations) were developed for the software sound-generation technique and another two for the surface impact sound-generating technique. In the software sound generation technique, the first and the second discriminant score functions (equations) developed had Eigenvalue, % of the variance, cumulative % and canonical correlation values displayed in Table S10 (submitted as a supplementary document). The eigenvalue is the ratio between and within quality groups sum of squares and a higher value makes a discriminant score function (equation) a better predictor. So, for the software sound generation technique first, the discriminant score function (equation) was chosen because it has a higher eigenvalue and higher canonical correlation value than the second function (equation). These equations (functions) are displayed in Table S10. In the surface impact sound-generating technique the first and the second discriminant score functions (equations) developed had Eigenvalue, % of the variance, cumulative % and canonical correlation values displayed in Table S10 (submitted as a supplementary document). So, for the impact sound-generating technique the first discriminant score function (equation) was chosen because it has a higher eigenvalue and higher canonical correlation value than the second function (equation). These equations (functions) are also displayed in Table S10 (table too large for manuscript) submitted as a supplementary document. Wilks’ lambda test was also done to the discriminant score functions (equations) for both techniques. This test shows that for the software sound generation technique the wilks' lambda value, chi-square value, degree of freedom and P-value for the two developed discriminant score functions (equations) were displayed in Table S10 (submitted as a supplementary document). The first discriminant score function (equation) was chosen over the second because it has a lower wilks' lambda value and is affect the detection. Also, this test for the surface impact sound-generating technique shows the wilks' lambda value, chi-square value, degree of freedom and P- value for the two developed discriminant score functions (equations) displayed in Table S10 (submitted as a supplementary document). The first discriminant score function (equation) was chosen over the second because it has a lower wilks' lambda value and is affecting the detection classification. The standardized discriminant score function coefficients test was done for functions (equations) developed for both techniques. This test shows the importance of the coefficients to the developed functions (equations). In the software sound generation-technique five acoustic properties, amplitude, frequency, velocity, wavelength and intensity were considered. The importance of the values of the coefficients was displayed in Table S10 (submitted as a supplementary document) for the first and second discriminant score function (equation) developed. These values show that only five of the coefficients of all-acoustic properties studied in the first developed function (equation) were important to it. In the surface impact, sound-generating techniques all six acoustic properties studied which were amplitude (db), frequency (Hz)), velocity (m/s), wavelength (m), intensity (db) and period (s) were considered. These coefficient values were displayed in Table S10 (submitted as a supplementary document) for the first and second discriminant score function (equation) developed respectively. These values show that for the first developed function (equation) some of the values of the coefficients have negative importance while in the second discriminant score function (equation) developed only the coefficient of wavelength has negative importance (Table S10) (tables too large for manuscript) submitted as a supplementary document. These phenomena can be attributed to the difference in weights of the different yam samples used for the surface impact experiments. A structure matrix test was also carried out for the function developed for both techniques. The structure matrix test tells the correction between the acoustic properties variable and the developed functions (equations). In the software sound generation technique, only five of the six acoustic properties were important to the two functions (equations) developed. In the surface impact sound-generating technique, all six acoustic properties were important to the two functions (equations) developed. The developed discriminant score functions (equations) for both techniques are displayed in equations (7)–(10) (see Table S10 in the supplementary document) [23,24]. observed similar discriminant pattern in their study of coconut quality detection and classification.

### 3.5 Software sound generation technique

(7) DSF1=−6.038E−07A+5.227E−02F−8.996E−06V+1.584E−06W+3.962E−08I−8.222

(8) DSF2=−5.045E−06A+4.817E−02F+8.912E−06V−4.866E−08W−2.484E−07I−8.093

### 3.6 Surface impact sound generation technique

(9) DSF1=−7.034E−06A−4.033E+01T+2.898E−02F−1.838E−06W−3.546E−07V+4.931E−07I−3.517

(10) DSF2=2.072E−05A+6.062E+01T+4.890E−03F−1.695E−05W+3.510E−07V+5.146E−06I−1.345E

Where

DSF1 is the discriminant score function (equation) for the first developed function (equation).

DSF2 is the discriminant score function (equation) for the second developed function (equation).

A = amplitude, F = frequency, I = intensity, T = period and W = wavelength, V = velocity.

The first discriminant score function (equation) was chosen in either technique for yellow yam quality detection classification.

The detection classification or confusion matrix result for the discriminant analysis detection of yellow yam quality, for both acoustic techniques used in this study is displayed in Table 4. This table shows that two experimental classifications were carried out for both acoustic techniques used in this study. These detection classifications were called original classification and validated classification. In the software sound generation technique, the actual detection classification result shows that of the 100 good yellow yams used for the detection. 87% of them were correctly detected and classified as yellow good yams, 10% were wrongly detected and classified as diseased damaged yellow yams and 3% were wrongly classified as insect-damaged yellow yams. Still on the original classification, of the 100 diseased damaged yellow yams used for the detection. 75% were correctly detected and classified as diseased damaged yellow yams. In comparison, none was wrongly detected and classified as good yellow yam and 25% were wrongly detected and classified as insect-damaged yellow yams. Again, still on the original classification, of the 100 insect-damaged yellow yams used for the detection. 87% were correctly detected and classified as insect-damaged yellow yams while 2% were wrongly detected and classified as good yellow yams and 11% were wrongly detected and classified as diseased damaged yams. The overall original classification performance of correct detections of all yellow yam quality considered in this study using the software sound generation technique for the original detection classification experiment was 83%. A validation detection experiment classification was done again to confirm the original result. In the validation detection and classifications experiment, of the 100 good yellow yams used for the detection and classifications. 86% were correctly detected and classified as good yellow yams, 11% were wrongly detected and classified as diseased damaged yellow yams and 3% were wrongly classified as insect-damaged yellow yams. Still on the validation classification, of the 100 diseased damaged yellow yams used for the detection. 74% were correctly detected and classified as diseased damaged yellow yams. In comparison, 1% were wrongly detected and classified as good yellow yams and 25% were wrongly detected and classified as insect-damaged yellow yams. Again, still on the validation classification, of the 100 insect-damaged yellow yams used for the detection. 87% were correctly detected and classified as insect-damaged yellow yams. In comparison, 2% were wrongly detected and classified as good yellow yams and 11% were wrongly detected and classified as diseased damaged yellow yams. The overall validated performance of correct detections and classification of all yellow yam quality considered in this study using the software sound generation technique was 82.3%. In the surface impact sound-generating technique, the actual detection classification result shows that of the 100 good yellow yams used for the detection. 47% of them were correctly detected and classified as good yellow yams, 48% were wrongly detected and classified as diseased damaged yellow yams and 5% were wrongly classified as insect-damaged yams. Still on the original classification, of the 100 diseased damaged yellow yams used for the detection. 61% were correctly detected and classified as diseased damaged yellow yams, 31% were wrongly detected and classified as good yellow yams and 8% were wrongly classified as insect-damaged yams. Again, still on the original classification, of the 100 insect-damaged yellow yams used for the detection. 100% were correctly detected and classified as insect-damaged yams while none were wrongly detected and classified as neither good yams nor diseased damaged yellow yams. The overall original performance classification of correct detections of all yellow yam quality considered in this study using the surface impact sound-generating technique was 69.3%. A validation detection experiment was done again to confirm the original result. In the validation detection and classifications experiment, of the 100 good yellow yams used for the detection and classifications. 47% were correctly detected and classified as good yellow yams, 48% were wrongly detected and classified as diseased damaged yellow yams and 5% were wrongly classified as insect-damaged yams. Still on the validation classification, of the 100 diseased damaged yellow yams used for the detection. 61% were correctly detected and classified as diseased damaged yellow yams, 31% were wrongly detected and classified as good yellow yams and 8% were wrongly classified as insect-damaged yams. Again, still on the validation classification, of the 100 insect-damaged yellow yams used for the detection. 98% were correctly detected and classified as insect-damaged yams while 1% were wrongly detected and classified as good yellow yams and 1% were wrongly detected and classified as diseased damaged yellow yams. The overall validated performance of classification of correct detections of all yellow yam quality considered in this study using the surface impact sound-generating technique was 68.7%. Comparing both acoustic techniques, the software sound generation-technique detected better yellow yams while the surface impact sound-generating technique detects more insect-damaged yellow yams. Both acoustic techniques detect diseased damaged yams equally well. Though, the overall performance of yellow yam quality detection was higher in the software sound generation technique. The surface impact sound-generating technique is the best to use when the goal of the detection was to detect insect damage to yellow yams. The acoustic detection of yellow yam tubers quality results achieved falls within the ranges obtained by Ref. [26] for the detection of the crispness of a carrot [23,24], for coconut quality detection, and below that obtained by Refs. [30,31] for the ripeness and texture of watermelon and strawberry.

**Table 4:** Classification or confusion matrix results for the discriminant analysis detection of yellow yam quality for both acoustic techniques.

Yam quality			Software Sound generation technique				Surface Impact sound-generating technique			
			Predicted Group Membership			Total	Predicted Group Membership			Total
			Good	Disease damaged	Insect Damaged		Good	Disease damaged	Insect Damaged	
Original	Count	Good	87	10	3	100	47	48	5	100
		Disease damaged	0	75	25	100	31	61	8	100
		Insect Damaged	2	11	87	100	0	0	100	100
	%	Good	87	10	3	100	47	48	5	100
		Disease damaged	0	75	25	100	31	61	8	100
		Insect Damaged	2	11	87	100	0	0	100	100
Cross-validated	Count	Good	86	11	3	100	47	48	5	100
		Disease damaged	1	74	25	100	31	61	8	100
		Insect Damaged	2	11	87	100	1	1	98	100
	%	Good	86	11	3	100	47	48	5	100
		Disease damaged	1	74	25	100	31	61	8	100
		Insect Damaged	2	11	87	100	1	1	98	100
Overall White yam quality detection			**83.0%** of original cases were correctly classified.				**69.3%** of original cases were correctly classified.			
			**82.3%** of cross-validated cases were correctly classified.				**68.7%** of cross-validated cases were correctly classified.			

Yellow yam should also be used as a game changer in the quest for global food security, due to its high calorific content per kg when compared to other crops. Therefore, there is a need to increase its export technological capability. This study developed a very cheap method to detect yellow yam quality for large export activity by just using its acoustic properties. These two developed techniques can be used to design different types of yam quality detection and separation equipment for yam exportation. The limitation of these developed techniques is that the user is expected to possess basic acoustic and computer knowledge.

## 4 Conclusions

This study developed two intelligent acoustic yam quality detection and classification devices. One uses a software sound-generation technique and the other uses a surface impact sound-generating technique. The machine learning algorithm used in the study called multiply discriminant analysis shows that fewer acoustic (sound) properties are needed for detecting yam quality in the software sound generation technique than in the surface impact sound-generating technique. Three qualities (good, diseased damaged and insect-damaged) of two yam varieties (white and yellow yam) were tested. The Overall validated detection and classification performance of white yam were 79% and 68.7% for the software sound generation technique and surface impact sound-generating technique respectively. The overall validated detection and classification performance of yellow yam was 82.3% and 68.7% for the software sound generation technique and surface impact sound-generating technique respectively. The study shows that the software sound-generation technique performed better than the surface impact sound-generating technique in terms of overall yam quality detection and classification. Nevertheless, the surface impact sound-generating technique detects and classified damaged yams better while the software sound-generation technique detected and classifies good quality yams better. Therefore, the choice of the technique to be recommended for yam quality detection will depend on the goal or final expected outcome of the yam processor. This study has shown that the acoustic properties of yam can be used to detect its quality and this acoustic was used to develop two smart devices. These two smart quality detecting technologies can also be incorporated into a yam planting machine, yam export quality detection line, yam storage warehouses and storage facilities, etc.

## Declarations

### Author contribution statement

John Audu: Conceived and designed the experiments; Performed the experiments; Analyzed and interpreted the data; Wrote the paper.

Rufus Dinrifo: Analyzed and interpreted the data; Wrote the paper.

Adeyemi Adegbenjo: Analyzed and interpreted the data; Contributed reagents, materials, analysis tools or data.

Peter Anyebe; Performed the experiments; Contributed reagents, materials, analysis tools or data; Wrote the paper.

Folarin Alonge: Conceived and designed the experiments; Contributed reagents, materials, analysis tools or data; Analyzed and interpreted the data.

### Funding statement

This research did not receive any specific grant from funding agencies in the public, commercial, or not-for-profit sectors.

### Data availability statement

Data included in article/supplementary material/referenced in article.

### Declaration of interest's statement

The authors declare no conflict of interest.

### Additional information

Supplementary content related to this article has been published online at [URL].
