# Supplementary material for: Development of two smart acoustic yam quality detection devices using a machine learning approach
Source: Heliyon. 2023 Mar 16;9(3):e14567. doi: 10.1016/j.heliyon.2023.e14567 (PMC10034441; doi:10.1016/j.heliyon.2023.e14567)
Supplement: Multimedia component 1 [file mmc1.docx]

**Development of Two Smart Acoustic Yam Quality Detection Devices Using a Machine Learning Approach**

^1^Audu* J., ^2^Dinrifo R. R., ^3^Adegbenjo A., ^4^Anyebe, S. P. and ^5^Alonge, A. F.

^1*^Department of Agricultural and Environmental Engineering, Federal University of Agriculture Makurdi, Nigeria

^2^Department of Agricultural Engineering, Lagos State Polytechnic Ikorodu, Nigeria

^3^Department of Agricultural and Environmental Engineering, Obafemi Awolowo University, Ife, Nigeria

^4^Department.of Agricultural and Bio - Env. Engineering Technology, Federal Polytechnic Nassarawa, Nigeria

^5^Department of Agricultural & Food Engineering, University of Uyo, Nigeria

*Corresponding author’s Email – audujoh@gmail.com

Table S1: Design information for design consideration of quality detection

| **Item/Requirement** | **Value/ content** | **Reason for selection** |
| --- | --- | --- |
| Outer body of the detection chamber | Mild density fibre board (MDF) | Material density of 850 kgm^-3^ and when double it is able to carry yam density range of 1000 to 1500 Kg^-3^. Also recommended for sound proof construction (https://in.pinterest.com/pin/746823550685383649/) |
| Inner body of the detection chamber | 12.5 mm Styrofoam | Recommended for used as sound proof material (https://www.soundproofcow.com/) |
| Speaker | 25 watts,4Ω subwoofer speaker | Used so that sounds will be audible enough. |
| Microphone | Multi-function Condenser Microphone | 3.5 mm Jack For Computers, Sensitivity: -55dB ± 2db, Sensitivity reduction: Within -3dB at 1.5 V, Operation voltage: 1.5V, Frequent response: 50Hz - 16KHz, S / N ratio: More than 36dB, height: 13.3cm |
| Sound detecting and Analyzing equipment | 2 Hp laptop computer |  |
| Sound Card | Focusrite Solo Soundcard -3rd Gen | For connecting a microphone and an instrument or line-level audio signals to a computer running Mac OS or Windows. physical inputs up to 24-bit, 192 kHz |
| Sound Amplifier | Vibes Cor VC AMP 12 BT Home Amplifier | Frequency required: 50 Hz, Voltage required: 220V, Power Source: Electric |
| Product use for detection | White yams and yellow yams | Quality parameter: Good, diseased and insect infection |

Table S2: Design of the acoustic intelligent devices

| **Parameter to design/select** | **Design Calculation/Value** | **Remark** |
| --- | --- | --- |
| Average Weight of yam (W_s_) | 2.5kg | Ijabo et al (2019) |
| Average height of yam (H_s_) | 350mm | Ijabo et al (2019 |
| Average breath of yam (B_s_) | 24cm | Ijabo et al (2019 |
| Average density of yam (ρs) | 1180kgm^-3^ | Ijabo et al (2019 |
| Density of MDF board (ρb) | 850kgm^-3^ | https://bettersoundproofing.com/drywall-osb-plywood-mdf-for-soundproofing |
| Coefficient of friction of yam on wood surface (µ) | 0.4 | Ijabo et al (2019) |
| Thickness of foam (T_f_) | 12.5mm | Recommend ((https://www.soundproofcow.com/) |
| Thickness of MDF board (T_b_) | 18mm |  |
| Diameter of speaker (Ds) | 125mm |  |
| Distance b/w speaker and sample holder (d_1_) | 50mm | Assumed |
| Distance b/w sample holder and microphone (d_2_) | 50mm | Assumed |
| Distance sound travelled (d1 +d2) | 100mm | Considering that for every 100mm, 0.06dB of the sound is reduced. This reduction is significant |
| Allowable space in the sample holder (A_sh_) | 40mm | Assumed |
| Allowable space in the sample sliding board (A_sr_) | 50mm on each side = 100mm | Assumed |
| Allowable distance between detecting chamber to top of sample (A_bc_) | 55mm | Assumed |
| **Software sound generation technique device** | | |
| Height of detecting chamber ((H_B_) | H_B_ = Hs+ 3T_b_ +A_bc_= 350 + 3 (18) + 55 = 459mm | Thickness of MDF was increased to withstand the weight of yam |
| Internal width of sample holder (W_ish_) | W_ish_ = B_s_ + A_sh_ = 80 + 40 = 120mm | B_s_ is the internal assumed width |
| External width of sample holder (W_esh_) | W_esh_ = B_s_ + A_sh_ + 4 (T_b_) = 120 + 4 (18) = 192mm |  |
| Height of sample holder (H_sh_) | H_sh_ = Tb +35% of height of sample = 18 + 122.5 = 140.5mm | 35% chosen so that yam would not fall off the sample holder |
| Width of detecting chamber (W_b_) | W_b_ = d_1_ + d_2_ +W_esh_ + 4(T_b_) = 100+ 100+192+4(18) = 464mm |  |
| Breadth of detecting chamber (B_b_) | B_b_ = 4(T_b_) +Ds+ 2(d_1_ + d_2_) = 4(18) + 125+ 2(100) = 397mm |  |
| Area of detection chamber (A_ch_) | A_ch_ = 459mm × 464mm = 212, 976 mm^2^ |  |
| Volume of detection chamber (V_ch_) | V_ch_ = 459mm × 464mm × 397mm = 84, 551, 472 mm^3^ |  |
| **Sample impact sound generation technique device** | | |
| Width of sliding chamber (W_sb_) | W_sb_ = H_s_ + 4(T_b_) + A_sr_ = 350 + 72 + 100 = 522mm |  |
| Height of the sliding chamber (H_sb_) | µ = H_sb_/W_sb_  0.4 = H_sb_/522  H_sb_ = 208.8 +3(T_b_) +Bs = 382.8mm |  |
| Breadth of sliding chamber (B_sb_) | (SP)^2^ = (H_sb_)^2^ + (B_sb_)^2^  (522)^2^ = (382.8)^2^ + (B_sb_)^2^  B_sb_ = 478.42 mm | SP = Sliding platform |
| Area of detection slide chamber (A_sch_) | A_sch_ = 382.8mm × 522mm = 199, 821.6 mm^2^ |  |
| Volume of detection slide chamber (V_sch_) | V_sch_ = 382.8 mm × 522 mm × 478.42 mm  = 95, 598, 645.872 mm^3^ |  |

Table S3: Acoustic properties of white yam qualities for sound generation technique and their classification training result using machine learning algorithm

| SN | Yam quality | A | F | I | T | V | ʎ | Pred. Yam quality | DSF1 | DSF2 | PMC1 | PMC2 | PMC3 |
| --- | --- | --- | --- | --- | --- | --- | --- | --- | --- | --- | --- | --- | --- |
| 1 | Good | 267.470 | 200 | 2139.760 | 5 | 25 | 0.125 | Good | -1.766 | -0.145 | 0.762 | 0.002 | 0.236 |
| 2 | Good | 281.720 | 200 | 2253.760 | 5 | 25 | 0.125 | Insect Damaged | -0.536 | -0.084 | 0.376 | 0.048 | 0.575 |
| 3 | Good | 288.660 | 200 | 2309.280 | 5 | 25 | 0.125 | Insect Damaged | 0.063 | -0.054 | 0.192 | 0.169 | 0.639 |
| 4 | Good | 267.490 | 200 | 2139.920 | 5 | 25 | 0.125 | Good | -1.765 | -0.144 | 0.762 | 0.002 | 0.236 |
| 5 | Good | 258.650 | 200 | 2069.200 | 5 | 25 | 0.125 | Good | -2.528 | -0.182 | 0.897 | 0.000 | 0.103 |
| 6 | Good | 271.410 | 200 | 2171.280 | 5 | 25 | 0.125 | Good | -1.426 | -0.128 | 0.672 | 0.005 | 0.323 |
| 7 | Good | 288.680 | 200 | 2309.440 | 5 | 25 | 0.125 | Insect Damaged | 0.065 | -0.054 | 0.192 | 0.170 | 0.639 |
| 8 | Good | 288.530 | 200 | 2308.240 | 5 | 25 | 0.125 | Insect Damaged | 0.052 | -0.055 | 0.195 | 0.166 | 0.639 |
| 9 | Good | 243.860 | 200 | 1950.880 | 5 | 25 | 0.125 | Good | -3.804 | -0.245 | 0.979 | 0.000 | 0.021 |
| 10 | Good | 274.720 | 200 | 2197.760 | 5 | 25 | 0.125 | Good | -1.141 | -0.114 | 0.583 | 0.011 | 0.407 |
| 11 | Good | 278.410 | 200 | 2227.280 | 5 | 25 | 0.125 | Insect Damaged | -0.822 | -0.098 | 0.475 | 0.024 | 0.501 |
| 12 | Good | 268.410 | 200 | 2147.280 | 5 | 25 | 0.125 | Good | -1.685 | -0.141 | 0.742 | 0.002 | 0.255 |
| 13 | Good | 267.120 | 200 | 2136.960 | 5 | 25 | 0.125 | Good | -1.797 | -0.146 | 0.769 | 0.002 | 0.229 |
| 14 | Good | 258.640 | 200 | 2069.120 | 5 | 25 | 0.125 | Good | -2.529 | -0.182 | 0.897 | 0.000 | 0.103 |
| 15 | Good | 275.490 | 200 | 2203.920 | 5 | 25 | 0.125 | Good | -1.074 | -0.110 | 0.561 | 0.013 | 0.426 |
| 16 | Good | 267.520 | 200 | 2140.160 | 5 | 25 | 0.125 | Good | -1.762 | -0.144 | 0.761 | 0.002 | 0.237 |
| 17 | Good | 271.460 | 200 | 2171.680 | 5 | 25 | 0.125 | Good | -1.422 | -0.128 | 0.670 | 0.005 | 0.325 |
| 18 | Good | 284.750 | 200 | 2278.000 | 5 | 25 | 0.125 | Insect Damaged | -0.275 | -0.071 | 0.290 | 0.086 | 0.623 |
| 19 | Good | 282.200 | 200 | 2257.600 | 5 | 25 | 0.125 | Insect Damaged | -0.495 | -0.082 | 0.362 | 0.053 | 0.585 |
| 20 | Good | 276.490 | 200 | 221192.000 | 5 | 25 | 0.125 | Good | -2.334 | 17.167 | 0.977 | 0.000 | 0.023 |
| 21 | Good | 271.510 | 200 | 2172.080 | 5 | 25 | 0.125 | Good | -1.418 | -0.127 | 0.669 | 0.005 | 0.326 |
| 22 | Good | 288.660 | 200 | 2309.280 | 5 | 25 | 0.125 | Insect Damaged | 0.063 | -0.054 | 0.192 | 0.169 | 0.639 |
| 23 | Good | 278.120 | 200 | 2224.960 | 5 | 25 | 0.125 | Insect Damaged | -0.847 | -0.099 | 0.484 | 0.023 | 0.494 |
| 24 | Good | 276.680 | 200 | 2213.440 | 5 | 25 | 0.125 | Good | -0.971 | -0.105 | 0.526 | 0.017 | 0.457 |
| 25 | Good | 258.450 | 200 | 2067.700 | 5 | 25 | 0.125 | Good | -2.545 | -0.183 | 0.899 | 0.000 | 0.101 |
| 26 | Good | 276.890 | 200 | 2215.120 | 5 | 25 | 0.125 | Good | -0.953 | -0.104 | 0.520 | 0.017 | 0.463 |
| 27 | Good | 248.940 | 200 | 1991.520 | 5 | 25 | 0.125 | Good | -3.366 | -0.224 | 0.963 | 0.000 | 0.037 |
| 28 | Good | 275.790 | 200 | 2206.320 | 5 | 25 | 0.125 | Good | -1.048 | -0.109 | 0.552 | 0.014 | 0.434 |
| 29 | Good | 267.620 | 200 | 2140.960 | 5 | 25 | 0.125 | Good | -1.753 | -0.144 | 0.759 | 0.002 | 0.239 |
| 30 | Good | 275.480 | 200 | 2203.840 | 5 | 25 | 0.125 | Good | -1.075 | -0.110 | 0.561 | 0.013 | 0.426 |
| 31 | Good | 284.730 | 200 | 2277.840 | 5 | 25 | 0.125 | Insect Damaged | -0.276 | -0.071 | 0.291 | 0.086 | 0.623 |
| 32 | Good | 223.540 | 200 | 1788.320 | 5 | 25 | 0.125 | Good | -5.559 | -0.332 | 0.998 | 0.000 | 0.002 |
| 33 | Good | 276.750 | 200 | 2214.000 | 5 | 25 | 0.125 | Good | -0.965 | -0.105 | 0.524 | 0.017 | 0.459 |
| 34 | Good | 275.960 | 200 | 2207.680 | 5 | 25 | 0.125 | Good | -1.033 | -0.108 | 0.547 | 0.014 | 0.439 |
| 35 | Good | 285.230 | 200 | 2281.840 | 5 | 25 | 0.125 | Insect Damaged | -0.233 | -0.069 | 0.277 | 0.094 | 0.628 |
| 36 | Good | 263.810 | 200 | 2110.480 | 5 | 25 | 0.125 | Good | -2.082 | -0.160 | 0.829 | 0.001 | 0.170 |
| 37 | Good | 223.510 | 200 | 1788.080 | 5 | 25 | 0.125 | Good | -5.561 | -0.332 | 0.998 | 0.000 | 0.002 |
| 38 | Good | 223.540 | 200 | 1788.320 | 5 | 25 | 0.125 | Good | -5.559 | -0.332 | 0.998 | 0.000 | 0.002 |
| 39 | Good | 273.740 | 200 | 2189.920 | 5 | 25 | 0.125 | Good | -1.225 | -0.118 | 0.610 | 0.009 | 0.381 |
| 40 | Good | 264.830 | 200 | 2118.640 | 5 | 25 | 0.125 | Good | -1.994 | -0.156 | 0.812 | 0.001 | 0.187 |
| 41 | Good | 259.190 | 200 | 2073.530 | 5 | 25 | 0.125 | Good | -2.481 | -0.180 | 0.891 | 0.000 | 0.109 |
| 42 | Good | 278.390 | 200 | 2227.120 | 5 | 25 | 0.125 | Insect Damaged | -0.824 | -0.098 | 0.475 | 0.024 | 0.500 |
| 43 | Good | 269.210 | 200 | 2153.680 | 5 | 25 | 0.125 | Good | -1.616 | -0.137 | 0.725 | 0.003 | 0.273 |
| 44 | Good | 283.840 | 200 | 2270.720 | 5 | 25 | 0.125 | Insect Damaged | -0.353 | -0.075 | 0.316 | 0.073 | 0.612 |
| 45 | Good | 279.240 | 200 | 2233.920 | 5 | 25 | 0.125 | Insect Damaged | -0.750 | -0.094 | 0.450 | 0.029 | 0.521 |
| 46 | Good | 243.850 | 200 | 1950.800 | 5 | 25 | 0.125 | Good | -3.805 | -0.245 | 0.979 | 0.000 | 0.021 |
| 47 | Good | 290.110 | 200 | 2160.880 | 5 | 25 | 0.125 | Insect Damaged | 0.189 | -0.061 | 0.160 | 0.212 | 0.628 |
| 48 | Good | 274.840 | 200 | 2198.720 | 5 | 25 | 0.125 | Good | -1.130 | -0.113 | 0.579 | 0.011 | 0.410 |
| 49 | Good | 257.950 | 200 | 2063.600 | 5 | 25 | 0.125 | Good | -2.588 | -0.185 | 0.904 | 0.000 | 0.096 |
| 50 | Good | 285.490 | 200 | 2283.920 | 5 | 25 | 0.125 | Insect Damaged | -0.211 | -0.068 | 0.270 | 0.099 | 0.631 |
| 51 | Good | 256.870 | 200 | 2054.960 | 5 | 25 | 0.125 | Good | -2.681 | -0.190 | 0.914 | 0.000 | 0.086 |
| 52 | Good | 256.760 | 200 | 2053.920 | 5 | 25 | 0.125 | Good | -2.691 | -0.190 | 0.915 | 0.000 | 0.085 |
| 53 | Good | 265.740 | 200 | 2125.920 | 5 | 25 | 0.125 | Good | -1.916 | -0.152 | 0.796 | 0.001 | 0.203 |
| 54 | Good | 256.890 | 200 | 2055.120 | 5 | 25 | 0.125 | Good | -2.680 | -0.190 | 0.913 | 0.000 | 0.086 |
| 55 | Good | 275.850 | 200 | 2206.800 | 5 | 25 | 0.125 | Good | -1.043 | -0.109 | 0.550 | 0.014 | 0.436 |
| 56 | Good | 274.850 | 200 | 2198.800 | 5 | 25 | 0.125 | Good | -1.129 | -0.113 | 0.579 | 0.011 | 0.410 |
| 57 | Good | 256.830 | 200 | 2054.640 | 5 | 25 | 0.125 | Good | -2.685 | -0.190 | 0.914 | 0.000 | 0.086 |
| 58 | Good | 271.590 | 200 | 2172.720 | 5 | 25 | 0.125 | Good | -1.411 | -0.127 | 0.667 | 0.005 | 0.328 |
| 59 | Good | 263.970 | 200 | 2111.760 | 5 | 25 | 0.125 | Good | -2.068 | -0.159 | 0.826 | 0.001 | 0.173 |
| 60 | Good | 268.430 | 200 | 2147.440 | 5 | 25 | 0.125 | Good | -1.683 | -0.140 | 0.742 | 0.002 | 0.256 |
| 61 | Good | 284.950 | 200 | 2279.600 | 5 | 25 | 0.125 | Insect Damaged | -0.257 | -0.070 | 0.285 | 0.090 | 0.625 |
| 62 | Good | 254.740 | 200 | 2037.920 | 5 | 25 | 0.125 | Good | -2.865 | -0.199 | 0.931 | 0.000 | 0.069 |
| 63 | Good | 277.650 | 200 | 2221.200 | 5 | 25 | 0.125 | Good | -0.888 | -0.101 | 0.497 | 0.021 | 0.482 |
| 64 | Good | 282.870 | 200 | 2262.960 | 5 | 25 | 0.125 | Insect Damaged | -0.437 | -0.079 | 0.343 | 0.061 | 0.596 |
| 65 | Good | 265.750 | 200 | 2126.000 | 5 | 25 | 0.125 | Good | -1.915 | -0.152 | 0.796 | 0.001 | 0.203 |
| 66 | Good | 284.910 | 200 | 2279.280 | 5 | 25 | 0.125 | Insect Damaged | -0.261 | -0.070 | 0.286 | 0.089 | 0.625 |
| 67 | Good | 272.840 | 200 | 2182.720 | 5 | 25 | 0.125 | Good | -1.303 | -0.122 | 0.635 | 0.007 | 0.359 |
| 68 | Good | 254.730 | 200 | 2037.840 | 5 | 25 | 0.125 | Good | -2.866 | -0.199 | 0.931 | 0.000 | 0.069 |
| 69 | Good | 272.860 | 200 | 2182.880 | 5 | 25 | 0.125 | Good | -1.301 | -0.122 | 0.634 | 0.007 | 0.359 |
| 70 | Good | 287.940 | 200 | 2303.520 | 5 | 25 | 0.125 | Insect Damaged | 0.001 | -0.057 | 0.209 | 0.151 | 0.641 |
| 71 | Good | 282.830 | 200 | 2262.640 | 5 | 25 | 0.125 | Insect Damaged | -0.440 | -0.079 | 0.344 | 0.060 | 0.596 |
| 72 | Good | 239.220 | 200 | 1913.760 | 5 | 25 | 0.125 | Good | -4.205 | -0.265 | 0.987 | 0.000 | 0.013 |
| 73 | Good | 274.750 | 200 | 2198.000 | 5 | 25 | 0.125 | Good | -1.138 | -0.114 | 0.582 | 0.011 | 0.407 |
| 74 | Good | 274.710 | 200 | 2197.680 | 5 | 25 | 0.125 | Good | -1.141 | -0.114 | 0.583 | 0.011 | 0.406 |
| 75 | Good | 256.850 | 200 | 2054.800 | 5 | 25 | 0.125 | Good | -2.683 | -0.190 | 0.914 | 0.000 | 0.086 |
| 76 | Good | 274.950 | 200 | 2199.600 | 5 | 25 | 0.125 | Good | -1.121 | -0.113 | 0.576 | 0.011 | 0.412 |
| 77 | Good | 264.880 | 200 | 2119.040 | 5 | 25 | 0.125 | Good | -1.990 | -0.156 | 0.811 | 0.001 | 0.188 |
| 78 | Good | 274.450 | 200 | 2195.600 | 5 | 25 | 0.125 | Good | -1.164 | -0.115 | 0.590 | 0.010 | 0.400 |
| 79 | Good | 284.540 | 200 | 2276.320 | 5 | 25 | 0.125 | Insect Damaged | -0.293 | -0.072 | 0.296 | 0.083 | 0.621 |
| 80 | Good | 265.860 | 200 | 2126.880 | 5 | 25 | 0.125 | Good | -1.905 | -0.151 | 0.794 | 0.001 | 0.205 |
| 81 | Good | 284.760 | 200 | 2278.080 | 5 | 25 | 0.125 | Insect Damaged | -0.274 | -0.071 | 0.290 | 0.087 | 0.623 |
| 82 | Good | 264.780 | 200 | 2118.240 | 5 | 25 | 0.125 | Good | -1.999 | -0.156 | 0.813 | 0.001 | 0.186 |
| 83 | Good | 281.540 | 200 | 2252.320 | 5 | 25 | 0.125 | Insect Damaged | -0.552 | -0.085 | 0.382 | 0.047 | 0.572 |
| 84 | Good | 264.730 | 200 | 2117.840 | 5 | 25 | 0.125 | Good | -2.003 | -0.156 | 0.814 | 0.001 | 0.185 |
| 85 | Good | 285.750 | 200 | 2286.000 | 5 | 25 | 0.125 | Insect Damaged | -0.188 | -0.067 | 0.264 | 0.104 | 0.633 |
| 86 | Good | 264.750 | 200 | 2118.000 | 5 | 25 | 0.125 | Good | -2.001 | -0.156 | 0.813 | 0.001 | 0.186 |
| 87 | Good | 283.590 | 200 | 2268.720 | 5 | 25 | 0.125 | Insect Damaged | -0.375 | -0.076 | 0.323 | 0.070 | 0.608 |
| 88 | Good | 245.380 | 200 | 1963.040 | 5 | 25 | 0.125 | Good | -3.673 | -0.239 | 0.975 | 0.000 | 0.025 |
| 89 | Good | 286.530 | 200 | 292.240 | 5 | 25 | 0.125 | Insect Damaged | -0.109 | -0.221 | 0.237 | 0.121 | 0.642 |
| 90 | Good | 284.460 | 200 | 2275.680 | 5 | 25 | 0.125 | Insect Damaged | -0.300 | -0.072 | 0.298 | 0.082 | 0.620 |
| 91 | Good | 286.530 | 200 | 2292.240 | 5 | 25 | 0.125 | Insect Damaged | -0.121 | -0.063 | 0.243 | 0.119 | 0.638 |
| 92 | Good | 264.840 | 200 | 2118.720 | 5 | 25 | 0.125 | Good | -1.993 | -0.156 | 0.812 | 0.001 | 0.187 |
| 93 | Good | 285.640 | 200 | 2284.120 | 5 | 25 | 0.125 | Insect Damaged | -0.198 | -0.067 | 0.266 | 0.102 | 0.632 |
| 94 | Good | 272.850 | 200 | 2182.800 | 5 | 25 | 0.125 | Good | -1.302 | -0.122 | 0.634 | 0.007 | 0.359 |
| 95 | Good | 264.940 | 200 | 2119.520 | 5 | 25 | 0.125 | Good | -1.985 | -0.155 | 0.810 | 0.001 | 0.189 |
| 96 | Good | 274.640 | 200 | 2197.120 | 5 | 25 | 0.125 | Good | -1.147 | -0.114 | 0.585 | 0.010 | 0.404 |
| 97 | Good | 283.880 | 200 | 2271.040 | 5 | 25 | 0.125 | Insect Damaged | -0.350 | -0.075 | 0.314 | 0.073 | 0.612 |
| 98 | Good | 273.850 | 200 | 2190.800 | 5 | 25 | 0.125 | Good | -1.216 | -0.117 | 0.607 | 0.009 | 0.384 |
| 99 | Good | 256.350 | 200 | 2050.800 | 5 | 25 | 0.125 | Good | -2.726 | -0.192 | 0.918 | 0.000 | 0.082 |
| 100 | Good | 274.650 | 200 | 2197.200 | 5 | 25 | 0.125 | Good | -1.147 | -0.114 | 0.585 | 0.011 | 0.405 |
| 101 | Disease damaged | 253.640 | 200 | 2029.120 | 5 | 25 | 0.125 | Good | -2.960 | -0.203 | 0.938 | 0.000 | 0.062 |
| 102 | Disease damaged | 307.720 | 200 | 2461.760 | 5 | 25 | 0.125 | Disease damaged | 1.708 | 0.027 | 0.005 | 0.858 | 0.137 |
| 103 | Disease damaged | 305.840 | 200 | 2446.720 | 5 | 25 | 0.125 | Disease damaged | 1.546 | 0.019 | 0.008 | 0.814 | 0.178 |
| 104 | Disease damaged | 311.220 | 200 | 2489.760 | 5 | 25 | 0.125 | Disease damaged | 2.010 | 0.042 | 0.002 | 0.916 | 0.082 |
| 105 | Disease damaged | 307.860 | 200 | 2462.880 | 5 | 25 | 0.125 | Disease damaged | 1.720 | 0.028 | 0.005 | 0.861 | 0.134 |
| 106 | Disease damaged | 307.850 | 200 | 2462.800 | 5 | 25 | 0.125 | Disease damaged | 1.719 | 0.027 | 0.005 | 0.861 | 0.135 |
| 107 | Disease damaged | 300.120 | 200 | 2400.960 | 5 | 25 | 0.125 | Disease damaged | 1.052 | -0.005 | 0.029 | 0.621 | 0.350 |
| 108 | Disease damaged | 307.840 | 200 | 2462.720 | 5 | 25 | 0.125 | Disease damaged | 1.719 | 0.027 | 0.005 | 0.860 | 0.135 |
| 109 | Disease damaged | 307.960 | 200 | 2463.680 | 5 | 25 | 0.125 | Disease damaged | 1.729 | 0.028 | 0.005 | 0.863 | 0.133 |
| 110 | Disease damaged | 300.220 | 200 | 2401.760 | 5 | 25 | 0.125 | Disease damaged | 1.061 | -0.005 | 0.029 | 0.625 | 0.347 |
| 111 | Disease damaged | 307.840 | 200 | 2462.720 | 5 | 25 | 0.125 | Disease damaged | 1.719 | 0.027 | 0.005 | 0.860 | 0.135 |
| 112 | Disease damaged | 307.860 | 200 | 2462.880 | 5 | 25 | 0.125 | Disease damaged | 1.720 | 0.028 | 0.005 | 0.861 | 0.134 |
| 113 | Disease damaged | 309.840 | 200 | 2478.720 | 5 | 25 | 0.125 | Disease damaged | 1.891 | 0.036 | 0.003 | 0.896 | 0.101 |
| 114 | Disease damaged | 307.850 | 200 | 2462.800 | 5 | 25 | 0.125 | Disease damaged | 1.719 | 0.027 | 0.005 | 0.861 | 0.135 |
| 115 | Disease damaged | 307.960 | 200 | 2463.680 | 5 | 25 | 0.125 | Disease damaged | 1.729 | 0.028 | 0.005 | 0.863 | 0.133 |
| 116 | Disease damaged | 302.720 | 200 | 2421.760 | 5 | 25 | 0.125 | Disease damaged | 1.277 | 0.006 | 0.016 | 0.720 | 0.264 |
| 117 | Disease damaged | 306.940 | 200 | 2455.520 | 5 | 25 | 0.125 | Disease damaged | 1.641 | 0.024 | 0.006 | 0.841 | 0.153 |
| 118 | Disease damaged | 308.650 | 200 | 2469.200 | 5 | 25 | 0.125 | Disease damaged | 1.788 | 0.031 | 0.004 | 0.876 | 0.120 |
| 119 | Disease damaged | 321.340 | 200 | 2570.720 | 5 | 25 | 0.125 | Disease damaged | 2.884 | 0.085 | 0.000 | 0.983 | 0.016 |
| 120 | Disease damaged | 308.740 | 200 | 2469.920 | 5 | 25 | 0.125 | Disease damaged | 1.796 | 0.031 | 0.004 | 0.878 | 0.119 |
| 121 | Disease damaged | 307.870 | 200 | 2463.680 | 5 | 25 | 0.125 | Disease damaged | 1.721 | 0.028 | 0.005 | 0.861 | 0.134 |
| 122 | Disease damaged | 307.960 | 200 | 2463.680 | 5 | 25 | 0.125 | Disease damaged | 1.729 | 0.028 | 0.005 | 0.863 | 0.133 |
| 123 | Disease damaged | 307.850 | 200 | 2462.800 | 5 | 25 | 0.125 | Disease damaged | 1.719 | 0.027 | 0.005 | 0.861 | 0.135 |
| 124 | Disease damaged | 307.850 | 200 | 2462.800 | 5 | 25 | 0.125 | Disease damaged | 1.719 | 0.027 | 0.005 | 0.861 | 0.135 |
| 125 | Disease damaged | 307.860 | 200 | 2462.880 | 5 | 25 | 0.125 | Disease damaged | 1.720 | 0.028 | 0.005 | 0.861 | 0.134 |
| 126 | Disease damaged | 307.840 | 200 | 2462.720 | 5 | 25 | 0.125 | Disease damaged | 1.719 | 0.027 | 0.005 | 0.860 | 0.135 |
| 127 | Disease damaged | 311.540 | 200 | 2492.320 | 5 | 25 | 0.125 | Disease damaged | 2.038 | 0.043 | 0.002 | 0.920 | 0.078 |
| 128 | Disease damaged | 312.240 | 200 | 2497.920 | 5 | 25 | 0.125 | Disease damaged | 2.098 | 0.046 | 0.002 | 0.928 | 0.070 |
| 129 | Disease damaged | 315.170 | 200 | 2521.360 | 5 | 25 | 0.125 | Disease damaged | 2.351 | 0.059 | 0.001 | 0.955 | 0.044 |
| 130 | Disease damaged | 305.940 | 200 | 2447.520 | 5 | 25 | 0.125 | Disease damaged | 1.555 | 0.019 | 0.008 | 0.817 | 0.175 |
| 131 | Disease damaged | 307.890 | 200 | 2463.120 | 5 | 25 | 0.125 | Disease damaged | 1.723 | 0.028 | 0.005 | 0.861 | 0.134 |
| 132 | Disease damaged | 305.780 | 200 | 2446.240 | 5 | 25 | 0.125 | Disease damaged | 1.541 | 0.019 | 0.008 | 0.813 | 0.179 |
| 133 | Disease damaged | 308.950 | 200 | 2471.600 | 5 | 25 | 0.125 | Disease damaged | 1.814 | 0.032 | 0.004 | 0.881 | 0.115 |
| 134 | Disease damaged | 307.340 | 200 | 2458.720 | 5 | 25 | 0.125 | Disease damaged | 1.675 | 0.025 | 0.005 | 0.850 | 0.145 |
| 135 | Disease damaged | 305.860 | 200 | 2446.880 | 5 | 25 | 0.125 | Disease damaged | 1.548 | 0.019 | 0.008 | 0.815 | 0.177 |
| 136 | Disease damaged | 307.850 | 200 | 2462.800 | 5 | 25 | 0.125 | Disease damaged | 1.719 | 0.027 | 0.005 | 0.861 | 0.135 |
| 137 | Disease damaged | 307.880 | 200 | 2463.040 | 5 | 25 | 0.125 | Disease damaged | 1.722 | 0.028 | 0.005 | 0.861 | 0.134 |
| 138 | Disease damaged | 311.230 | 200 | 2489.840 | 5 | 25 | 0.125 | Disease damaged | 2.011 | 0.042 | 0.002 | 0.916 | 0.082 |
| 139 | Disease damaged | 308.740 | 200 | 2469.920 | 5 | 25 | 0.125 | Disease damaged | 1.796 | 0.031 | 0.004 | 0.878 | 0.119 |
| 140 | Disease damaged | 307.950 | 200 | 2463.600 | 5 | 25 | 0.125 | Disease damaged | 1.728 | 0.028 | 0.005 | 0.863 | 0.133 |
| 141 | Disease damaged | 308.960 | 200 | 2471.680 | 5 | 25 | 0.125 | Disease damaged | 1.815 | 0.032 | 0.004 | 0.882 | 0.115 |
| 142 | Disease damaged | 304.640 | 200 | 2437.120 | 5 | 25 | 0.125 | Disease damaged | 1.442 | 0.014 | 0.010 | 0.781 | 0.208 |
| 143 | Disease damaged | 307.640 | 200 | 2461.120 | 5 | 25 | 0.125 | Disease damaged | 1.701 | 0.027 | 0.005 | 0.856 | 0.139 |
| 144 | Disease damaged | 312.590 | 200 | 2500.720 | 5 | 25 | 0.125 | Disease damaged | 2.129 | 0.048 | 0.001 | 0.932 | 0.066 |
| 145 | Disease damaged | 306.980 | 200 | 2455.840 | 5 | 25 | 0.125 | Disease damaged | 1.644 | 0.024 | 0.006 | 0.842 | 0.152 |
| 146 | Disease damaged | 305.970 | 200 | 2447.760 | 5 | 25 | 0.125 | Disease damaged | 1.557 | 0.019 | 0.008 | 0.818 | 0.175 |
| 147 | Disease damaged | 306.980 | 200 | 2455.840 | 5 | 25 | 0.125 | Disease damaged | 1.644 | 0.024 | 0.006 | 0.842 | 0.152 |
| 148 | Disease damaged | 306.980 | 200 | 2455.840 | 5 | 25 | 0.125 | Disease damaged | 1.644 | 0.024 | 0.006 | 0.842 | 0.152 |
| 149 | Disease damaged | 307.880 | 200 | 2463.040 | 5 | 25 | 0.125 | Disease damaged | 1.722 | 0.028 | 0.005 | 0.861 | 0.134 |
| 150 | Disease damaged | 307.750 | 200 | 2462.000 | 5 | 25 | 0.125 | Disease damaged | 1.711 | 0.027 | 0.005 | 0.859 | 0.137 |
| 151 | Disease damaged | 306.840 | 200 | 2462.720 | 5 | 25 | 0.125 | Disease damaged | 1.632 | 0.024 | 0.006 | 0.839 | 0.155 |
| 152 | Disease damaged | 304.120 | 200 | 2432.960 | 5 | 25 | 0.125 | Disease damaged | 1.397 | 0.012 | 0.012 | 0.766 | 0.222 |
| 153 | Disease damaged | 332.450 | 200 | 2659.600 | 5 | 25 | 0.125 | Disease damaged | 3.843 | 0.132 | 0.000 | 0.997 | 0.003 |
| 154 | Disease damaged | 303.210 | 200 | 2425.680 | 5 | 25 | 0.125 | Disease damaged | 1.319 | 0.008 | 0.015 | 0.737 | 0.249 |
| 155 | Disease damaged | 301.430 | 200 | 2411.440 | 5 | 25 | 0.125 | Disease damaged | 1.165 | 0.000 | 0.022 | 0.673 | 0.305 |
| 156 | Disease damaged | 307.850 | 200 | 2462.800 | 5 | 25 | 0.125 | Disease damaged | 1.719 | 0.027 | 0.005 | 0.861 | 0.135 |
| 157 | Disease damaged | 307.740 | 200 | 2461.920 | 5 | 25 | 0.125 | Disease damaged | 1.710 | 0.027 | 0.005 | 0.858 | 0.137 |
| 158 | Disease damaged | 308.650 | 200 | 2468.800 | 5 | 25 | 0.125 | Disease damaged | 1.788 | 0.031 | 0.004 | 0.876 | 0.120 |
| 159 | Disease damaged | 300.170 | 200 | 2401.360 | 5 | 25 | 0.125 | Disease damaged | 1.056 | -0.005 | 0.029 | 0.623 | 0.348 |
| 160 | Disease damaged | 309.680 | 200 | 2477.440 | 5 | 25 | 0.125 | Disease damaged | 1.877 | 0.035 | 0.003 | 0.894 | 0.103 |
| 161 | Disease damaged | 307.980 | 200 | 2463.840 | 5 | 25 | 0.125 | Disease damaged | 1.731 | 0.028 | 0.005 | 0.863 | 0.132 |
| 162 | Disease damaged | 306.950 | 200 | 2455.600 | 5 | 25 | 0.125 | Disease damaged | 1.642 | 0.024 | 0.006 | 0.841 | 0.153 |
| 163 | Disease damaged | 307.980 | 200 | 2463.840 | 5 | 25 | 0.125 | Disease damaged | 1.731 | 0.028 | 0.005 | 0.863 | 0.132 |
| 164 | Disease damaged | 312.970 | 200 | 2503.760 | 5 | 25 | 0.125 | Disease damaged | 2.161 | 0.049 | 0.001 | 0.936 | 0.063 |
| 165 | Disease damaged | 296.640 | 200 | 2373.120 | 5 | 25 | 0.125 | Insect Damaged | 0.752 | -0.020 | 0.058 | 0.470 | 0.472 |
| 166 | Disease damaged | 300.220 | 200 | 2401.760 | 5 | 25 | 0.125 | Disease damaged | 1.061 | -0.005 | 0.029 | 0.625 | 0.347 |
| 167 | Disease damaged | 298.890 | 200 | 2391.120 | 5 | 25 | 0.125 | Disease damaged | 0.946 | -0.011 | 0.038 | 0.569 | 0.393 |
| 168 | Disease damaged | 308.640 | 200 | 2469.120 | 5 | 25 | 0.125 | Disease damaged | 1.788 | 0.031 | 0.004 | 0.876 | 0.120 |
| 169 | Disease damaged | 307.760 | 200 | 2462.080 | 5 | 25 | 0.125 | Disease damaged | 1.712 | 0.027 | 0.005 | 0.859 | 0.136 |
| 170 | Disease damaged | 309.670 | 200 | 2477.360 | 5 | 25 | 0.125 | Disease damaged | 1.877 | 0.035 | 0.003 | 0.894 | 0.103 |
| 171 | Disease damaged | 306.780 | 200 | 2454.240 | 5 | 25 | 0.125 | Disease damaged | 1.627 | 0.023 | 0.006 | 0.837 | 0.156 |
| 172 | Disease damaged | 311.180 | 200 | 2489.440 | 5 | 25 | 0.125 | Disease damaged | 2.007 | 0.042 | 0.002 | 0.916 | 0.082 |
| 173 | Disease damaged | 324.360 | 200 | 2594.880 | 5 | 25 | 0.125 | Disease damaged | 3.145 | 0.098 | 0.000 | 0.990 | 0.010 |
| 174 | Disease damaged | 298.540 | 200 | 2388.320 | 5 | 25 | 0.125 | Disease damaged | 0.916 | -0.012 | 0.040 | 0.554 | 0.406 |
| 175 | Disease damaged | 319.870 | 200 | 2558.960 | 5 | 25 | 0.125 | Disease damaged | 2.757 | 0.079 | 0.000 | 0.979 | 0.021 |
| 176 | Disease damaged | 316.840 | 200 | 2534.720 | 5 | 25 | 0.125 | Disease damaged | 2.495 | 0.066 | 0.000 | 0.966 | 0.034 |
| 177 | Disease damaged | 302.850 | 200 | 2422.800 | 5 | 25 | 0.125 | Disease damaged | 1.288 | 0.006 | 0.016 | 0.724 | 0.260 |
| 178 | Disease damaged | 309.750 | 200 | 2478.000 | 5 | 25 | 0.125 | Disease damaged | 1.883 | 0.036 | 0.003 | 0.895 | 0.102 |
| 179 | Disease damaged | 307.650 | 200 | 2461.200 | 5 | 25 | 0.125 | Disease damaged | 1.702 | 0.027 | 0.005 | 0.856 | 0.139 |
| 180 | Disease damaged | 310.850 | 200 | 2486.800 | 5 | 25 | 0.125 | Disease damaged | 1.978 | 0.040 | 0.002 | 0.911 | 0.087 |
| 181 | Disease damaged | 307.840 | 200 | 2462.720 | 5 | 25 | 0.125 | Disease damaged | 1.719 | 0.027 | 0.005 | 0.860 | 0.135 |
| 182 | Disease damaged | 306.670 | 200 | 2453.360 | 5 | 25 | 0.125 | Disease damaged | 1.618 | 0.022 | 0.006 | 0.835 | 0.159 |
| 183 | Disease damaged | 309.640 | 200 | 2477.120 | 5 | 25 | 0.125 | Disease damaged | 1.874 | 0.035 | 0.003 | 0.893 | 0.104 |
| 184 | Disease damaged | 299.840 | 200 | 2398.720 | 5 | 25 | 0.125 | Disease damaged | 1.028 | -0.007 | 0.031 | 0.609 | 0.360 |
| 185 | Disease damaged | 307.860 | 200 | 2462.880 | 5 | 25 | 0.125 | Disease damaged | 1.720 | 0.028 | 0.005 | 0.861 | 0.134 |
| 186 | Disease damaged | 309.670 | 200 | 2477.360 | 5 | 25 | 0.125 | Disease damaged | 1.877 | 0.035 | 0.003 | 0.894 | 0.103 |
| 187 | Disease damaged | 307.580 | 200 | 2460.640 | 5 | 25 | 0.125 | Disease damaged | 1.696 | 0.026 | 0.005 | 0.855 | 0.140 |
| 188 | Disease damaged | 296.990 | 200 | 2375.920 | 5 | 25 | 0.125 | Disease damaged | 0.782 | -0.019 | 0.054 | 0.486 | 0.460 |
| 189 | Disease damaged | 308.560 | 200 | 2468.480 | 5 | 25 | 0.125 | Disease damaged | 1.781 | 0.031 | 0.004 | 0.874 | 0.122 |
| 190 | Disease damaged | 328.560 | 200 | 2628.480 | 5 | 25 | 0.125 | Disease damaged | 3.507 | 0.116 | 0.000 | 0.995 | 0.005 |
| 191 | Disease damaged | 309.850 | 200 | 2478.800 | 5 | 25 | 0.125 | Disease damaged | 1.892 | 0.036 | 0.003 | 0.896 | 0.101 |
| 192 | Disease damaged | 304.730 | 200 | 2437.840 | 5 | 25 | 0.125 | Disease damaged | 1.450 | 0.014 | 0.010 | 0.784 | 0.206 |
| 193 | Disease damaged | 303.210 | 200 | 2425.680 | 5 | 25 | 0.125 | Disease damaged | 1.319 | 0.008 | 0.015 | 0.737 | 0.249 |
| 194 | Disease damaged | 308.750 | 200 | 2470.000 | 5 | 25 | 0.125 | Disease damaged | 1.797 | 0.031 | 0.004 | 0.878 | 0.118 |
| 195 | Disease damaged | 314.560 | 200 | 2516.480 | 5 | 25 | 0.125 | Disease damaged | 2.299 | 0.056 | 0.001 | 0.950 | 0.049 |
| 196 | Disease damaged | 321.130 | 200 | 2569.040 | 5 | 25 | 0.125 | Disease damaged | 2.866 | 0.084 | 0.000 | 0.983 | 0.017 |
| 197 | Disease damaged | 317.450 | 200 | 2539.600 | 5 | 25 | 0.125 | Disease damaged | 2.548 | 0.068 | 0.000 | 0.969 | 0.031 |
| 198 | Disease damaged | 287.450 | 200 | 2299.600 | 5 | 25 | 0.125 | Insect Damaged | -0.042 | -0.059 | 0.221 | 0.139 | 0.641 |
| 199 | Disease damaged | 306.840 | 200 | 2454.720 | 5 | 25 | 0.125 | Disease damaged | 1.632 | 0.023 | 0.006 | 0.839 | 0.155 |
| 200 | Disease damaged | 322.760 | 200 | 2582.080 | 5 | 25 | 0.125 | Disease damaged | 3.006 | 0.091 | 0.000 | 0.987 | 0.013 |
| 201 | Insect Damaged | 309.850 | 200 | 2478.800 | 5 | 25 | 0.125 | Disease damaged | 1.892 | 0.036 | 0.003 | 0.896 | 0.101 |
| 202 | Insect Damaged | 322.110 | 200 | 2576.880 | 5 | 25 | 0.125 | Disease damaged | 2.950 | 0.088 | 0.000 | 0.985 | 0.014 |
| 203 | Insect Damaged | 292.110 | 200 | 2336.880 | 5 | 25 | 0.125 | Insect Damaged | 0.361 | -0.040 | 0.122 | 0.281 | 0.597 |
| 204 | Insect Damaged | 279.860 | 200 | 2238.880 | 5 | 25 | 0.125 | Insect Damaged | -0.697 | -0.092 | 0.432 | 0.033 | 0.535 |
| 205 | Insect Damaged | 288.630 | 200 | 2309.040 | 5 | 25 | 0.125 | Insect Damaged | 0.060 | -0.054 | 0.193 | 0.169 | 0.639 |
| 206 | Insect Damaged | 288.760 | 200 | 2310.080 | 5 | 25 | 0.125 | Insect Damaged | 0.071 | -0.054 | 0.190 | 0.172 | 0.638 |
| 207 | Insect Damaged | 283.640 | 200 | 2269.120 | 5 | 25 | 0.125 | Insect Damaged | -0.371 | -0.076 | 0.321 | 0.070 | 0.609 |
| 208 | Insect Damaged | 294.220 | 200 | 2353.760 | 5 | 25 | 0.125 | Insect Damaged | 0.543 | -0.031 | 0.088 | 0.365 | 0.547 |
| 209 | Insect Damaged | 284.980 | 200 | 2279.840 | 5 | 25 | 0.125 | Insect Damaged | -0.255 | -0.070 | 0.284 | 0.090 | 0.626 |
| 210 | Insect Damaged | 291.730 | 200 | 2333.840 | 5 | 25 | 0.125 | Insect Damaged | 0.328 | -0.041 | 0.129 | 0.267 | 0.605 |
| 211 | Insect Damaged | 290.540 | 200 | 2462.880 | 5 | 25 | 0.125 | Insect Damaged | 0.224 | -0.035 | 0.152 | 0.225 | 0.623 |
| 212 | Insect Damaged | 293.260 | 200 | 2346.080 | 5 | 25 | 0.125 | Insect Damaged | 0.460 | -0.035 | 0.103 | 0.325 | 0.572 |
| 213 | Insect Damaged | 290.630 | 200 | 2325.040 | 5 | 25 | 0.125 | Insect Damaged | 0.233 | -0.046 | 0.150 | 0.229 | 0.622 |
| 214 | Insect Damaged | 290.540 | 200 | 2324.320 | 5 | 25 | 0.125 | Insect Damaged | 0.225 | -0.046 | 0.152 | 0.226 | 0.623 |
| 215 | Insect Damaged | 291.630 | 200 | 2333.040 | 5 | 25 | 0.125 | Insect Damaged | 0.319 | -0.042 | 0.131 | 0.263 | 0.606 |
| 216 | Insect Damaged | 290.630 | 200 | 2325.040 | 5 | 25 | 0.125 | Insect Damaged | 0.233 | -0.046 | 0.150 | 0.229 | 0.622 |
| 217 | Insect Damaged | 290.630 | 200 | 2325.040 | 5 | 25 | 0.125 | Insect Damaged | 0.233 | -0.046 | 0.150 | 0.229 | 0.622 |
| 218 | Insect Damaged | 291.650 | 200 | 2333.200 | 5 | 25 | 0.125 | Insect Damaged | 0.321 | -0.042 | 0.130 | 0.264 | 0.606 |
| 219 | Insect Damaged | 288.730 | 200 | 2309.840 | 5 | 25 | 0.125 | Insect Damaged | 0.069 | -0.054 | 0.190 | 0.171 | 0.638 |
| 220 | Insect Damaged | 274.940 | 200 | 2199.520 | 5 | 25 | 0.125 | Good | -1.122 | -0.113 | 0.577 | 0.011 | 0.412 |
| 221 | Insect Damaged | 287.640 | 200 | 2301.120 | 5 | 25 | 0.125 | Insect Damaged | -0.025 | -0.059 | 0.216 | 0.143 | 0.641 |
| 222 | Insect Damaged | 293.750 | 200 | 2350.000 | 5 | 25 | 0.125 | Insect Damaged | 0.502 | -0.033 | 0.095 | 0.345 | 0.560 |
| 223 | Insect Damaged | 254.730 | 200 | 2462.800 | 5 | 25 | 0.125 | Good | -2.869 | -0.165 | 0.931 | 0.000 | 0.069 |
| 224 | Insect Damaged | 278.840 | 200 | 2230.720 | 5 | 25 | 0.125 | Insect Damaged | -0.785 | -0.096 | 0.462 | 0.027 | 0.511 |
| 225 | Insect Damaged | 257.960 | 200 | 2063.680 | 5 | 25 | 0.125 | Good | -2.587 | -0.185 | 0.903 | 0.000 | 0.096 |
| 226 | Insect Damaged | 291.630 | 200 | 2333.040 | 5 | 25 | 0.125 | Insect Damaged | 0.319 | -0.042 | 0.131 | 0.263 | 0.606 |
| 227 | Insect Damaged | 289.670 | 200 | 2317.360 | 5 | 25 | 0.125 | Insect Damaged | 0.150 | -0.050 | 0.170 | 0.198 | 0.632 |
| 228 | Insect Damaged | 268.940 | 200 | 2151.520 | 5 | 25 | 0.125 | Good | -1.639 | -0.138 | 0.731 | 0.003 | 0.267 |
| 229 | Insect Damaged | 289.680 | 200 | 2317.440 | 5 | 25 | 0.125 | Insect Damaged | 0.151 | -0.050 | 0.169 | 0.198 | 0.632 |
| 230 | Insect Damaged | 258.750 | 200 | 2070.000 | 5 | 25 | 0.125 | Good | -2.519 | -0.182 | 0.895 | 0.000 | 0.104 |
| 231 | Insect Damaged | 287.640 | 200 | 2301.120 | 5 | 25 | 0.125 | Insect Damaged | -0.025 | -0.059 | 0.216 | 0.143 | 0.641 |
| 232 | Insect Damaged | 293.860 | 200 | 2350.880 | 5 | 25 | 0.125 | Insect Damaged | 0.512 | -0.032 | 0.093 | 0.350 | 0.557 |
| 233 | Insect Damaged | 286.940 | 200 | 2295.520 | 5 | 25 | 0.125 | Insect Damaged | -0.086 | -0.062 | 0.233 | 0.127 | 0.639 |
| 234 | Insect Damaged | 290.840 | 200 | 2326.720 | 5 | 25 | 0.125 | Insect Damaged | 0.251 | -0.045 | 0.146 | 0.236 | 0.619 |
| 235 | Insect Damaged | 291.640 | 200 | 2333.120 | 5 | 25 | 0.125 | Insect Damaged | 0.320 | -0.042 | 0.130 | 0.263 | 0.606 |
| 236 | Insect Damaged | 290.740 | 200 | 2325.920 | 5 | 25 | 0.125 | Insect Damaged | 0.242 | -0.045 | 0.148 | 0.232 | 0.620 |
| 237 | Insect Damaged | 292.630 | 200 | 2341.040 | 5 | 25 | 0.125 | Insect Damaged | 0.406 | -0.037 | 0.113 | 0.300 | 0.587 |
| 238 | Insect Damaged | 291.710 | 200 | 2333.680 | 5 | 25 | 0.125 | Insect Damaged | 0.326 | -0.041 | 0.129 | 0.266 | 0.605 |
| 239 | Insect Damaged | 278.750 | 200 | 2230.000 | 5 | 25 | 0.125 | Insect Damaged | -0.793 | -0.097 | 0.465 | 0.026 | 0.509 |
| 240 | Insect Damaged | 282.740 | 200 | 2261.920 | 5 | 25 | 0.125 | Insect Damaged | -0.448 | -0.080 | 0.347 | 0.059 | 0.594 |
| 241 | Insect Damaged | 279.900 | 200 | 2239.200 | 5 | 25 | 0.125 | Insect Damaged | -0.693 | -0.092 | 0.430 | 0.033 | 0.536 |
| 242 | Insect Damaged | 287.680 | 200 | 2301.440 | 5 | 25 | 0.125 | Insect Damaged | -0.022 | -0.058 | 0.215 | 0.144 | 0.641 |
| 243 | Insect Damaged | 286.990 | 200 | 2295.920 | 5 | 25 | 0.125 | Insect Damaged | -0.081 | -0.061 | 0.232 | 0.129 | 0.640 |
| 244 | Insect Damaged | 282.870 | 200 | 2262.960 | 5 | 25 | 0.125 | Insect Damaged | -0.437 | -0.079 | 0.343 | 0.061 | 0.596 |
| 245 | Insect Damaged | 294.110 | 200 | 2352.880 | 5 | 25 | 0.125 | Insect Damaged | 0.533 | -0.031 | 0.090 | 0.360 | 0.550 |
| 246 | Insect Damaged | 267.460 | 200 | 2139.680 | 5 | 25 | 0.125 | Good | -1.767 | -0.145 | 0.762 | 0.002 | 0.236 |
| 247 | Insect Damaged | 283.830 | 200 | 2270.640 | 5 | 25 | 0.125 | Insect Damaged | -0.354 | -0.075 | 0.316 | 0.073 | 0.611 |
| 248 | Insect Damaged | 281.890 | 200 | 2255.120 | 5 | 25 | 0.125 | Insect Damaged | -0.522 | -0.083 | 0.371 | 0.050 | 0.579 |
| 249 | Insect Damaged | 291.740 | 200 | 2333.920 | 5 | 25 | 0.125 | Insect Damaged | 0.329 | -0.041 | 0.129 | 0.267 | 0.604 |
| 250 | Insect Damaged | 288.690 | 200 | 2309.520 | 5 | 25 | 0.125 | Insect Damaged | 0.065 | -0.054 | 0.191 | 0.170 | 0.639 |
| 251 | Insect Damaged | 289.770 | 200 | 2318.160 | 5 | 25 | 0.125 | Insect Damaged | 0.159 | -0.050 | 0.168 | 0.201 | 0.631 |
| 252 | Insect Damaged | 274.820 | 200 | 2198.560 | 5 | 25 | 0.125 | Good | -1.132 | -0.113 | 0.580 | 0.011 | 0.409 |
| 253 | Insect Damaged | 292.560 | 200 | 2340.480 | 5 | 25 | 0.125 | Insect Damaged | 0.400 | -0.038 | 0.114 | 0.298 | 0.588 |
| 254 | Insect Damaged | 285.800 | 200 | 2286.400 | 5 | 25 | 0.125 | Insect Damaged | -0.184 | -0.066 | 0.262 | 0.105 | 0.633 |
| 255 | Insect Damaged | 263.540 | 200 | 2108.320 | 5 | 25 | 0.125 | Good | -2.106 | -0.161 | 0.833 | 0.001 | 0.166 |
| 256 | Insect Damaged | 287.640 | 200 | 2301.120 | 5 | 25 | 0.125 | Insect Damaged | -0.025 | -0.059 | 0.216 | 0.143 | 0.641 |
| 257 | Insect Damaged | 289.320 | 200 | 2314.560 | 5 | 25 | 0.125 | Insect Damaged | 0.120 | -0.051 | 0.177 | 0.188 | 0.635 |
| 258 | Insect Damaged | 258.610 | 200 | 2068.880 | 5 | 25 | 0.125 | Good | -2.531 | -0.182 | 0.897 | 0.000 | 0.103 |
| 259 | Insect Damaged | 286.670 | 200 | 2293.360 | 5 | 25 | 0.125 | Insect Damaged | -0.109 | -0.063 | 0.240 | 0.122 | 0.638 |
| 260 | Insect Damaged | 249.840 | 200 | 1998.720 | 5 | 25 | 0.125 | Good | -3.288 | -0.220 | 0.959 | 0.000 | 0.041 |
| 261 | Insect Damaged | 274.960 | 200 | 2199.680 | 5 | 25 | 0.125 | Good | -1.120 | -0.113 | 0.576 | 0.011 | 0.413 |
| 262 | Insect Damaged | 292.760 | 200 | 2342.080 | 5 | 25 | 0.125 | Insect Damaged | 0.417 | -0.037 | 0.111 | 0.305 | 0.584 |
| 263 | Insect Damaged | 286.840 | 200 | 2294.720 | 5 | 25 | 0.125 | Insect Damaged | -0.094 | -0.062 | 0.236 | 0.125 | 0.639 |
| 264 | Insect Damaged | 297.630 | 200 | 2381.040 | 5 | 25 | 0.125 | Disease damaged | 0.837 | -0.016 | 0.048 | 0.514 | 0.438 |
| 265 | Insect Damaged | 287.680 | 200 | 2301.440 | 5 | 25 | 0.125 | Insect Damaged | -0.022 | -0.058 | 0.215 | 0.144 | 0.641 |
| 266 | Insect Damaged | 299.990 | 200 | 2399.920 | 5 | 25 | 0.125 | Disease damaged | 1.041 | -0.006 | 0.030 | 0.615 | 0.355 |
| 267 | Insect Damaged | 296.850 | 200 | 2374.800 | 5 | 25 | 0.125 | Disease damaged | 0.770 | -0.019 | 0.056 | 0.479 | 0.465 |
| 268 | Insect Damaged | 301.850 | 200 | 2414.800 | 5 | 25 | 0.125 | Disease damaged | 1.201 | 0.002 | 0.020 | 0.689 | 0.291 |
| 269 | Insect Damaged | 293.900 | 200 | 2351.200 | 5 | 25 | 0.125 | Insect Damaged | 0.515 | -0.032 | 0.093 | 0.351 | 0.556 |
| 270 | Insect Damaged | 287.840 | 200 | 2302.720 | 5 | 25 | 0.125 | Insect Damaged | -0.008 | -0.058 | 0.211 | 0.148 | 0.641 |
| 271 | Insect Damaged | 292.940 | 200 | 2343.520 | 5 | 25 | 0.125 | Insect Damaged | 0.432 | -0.036 | 0.108 | 0.313 | 0.580 |
| 272 | Insect Damaged | 284.830 | 200 | 2278.640 | 5 | 25 | 0.125 | Insect Damaged | -0.268 | -0.071 | 0.288 | 0.088 | 0.624 |
| 273 | Insect Damaged | 292.730 | 200 | 2341.840 | 5 | 25 | 0.125 | Insect Damaged | 0.414 | -0.037 | 0.111 | 0.304 | 0.584 |
| 274 | Insect Damaged | 279.990 | 200 | 2239.920 | 5 | 25 | 0.125 | Insect Damaged | -0.686 | -0.091 | 0.428 | 0.034 | 0.538 |
| 275 | Insect Damaged | 302.360 | 200 | 2418.880 | 5 | 25 | 0.125 | Disease damaged | 1.245 | 0.004 | 0.018 | 0.707 | 0.275 |
| 276 | Insect Damaged | 294.820 | 200 | 2358.560 | 5 | 25 | 0.125 | Insect Damaged | 0.595 | -0.028 | 0.080 | 0.390 | 0.530 |
| 277 | Insect Damaged | 274.840 | 200 | 2198.720 | 5 | 25 | 0.125 | Good | -1.130 | -0.113 | 0.579 | 0.011 | 0.410 |
| 278 | Insect Damaged | 282.840 | 200 | 2262.720 | 5 | 25 | 0.125 | Insect Damaged | -0.440 | -0.079 | 0.344 | 0.060 | 0.596 |
| 279 | Insect Damaged | 296.790 | 200 | 2374.320 | 5 | 25 | 0.125 | Disease damaged | 0.765 | -0.020 | 0.056 | 0.477 | 0.467 |
| 280 | Insect Damaged | 289.110 | 200 | 2312.880 | 5 | 25 | 0.125 | Insect Damaged | 0.102 | -0.052 | 0.182 | 0.182 | 0.636 |
| 281 | Insect Damaged | 292.320 | 200 | 2338.560 | 5 | 25 | 0.125 | Insect Damaged | 0.379 | -0.039 | 0.118 | 0.289 | 0.593 |
| 282 | Insect Damaged | 287.530 | 200 | 2300.240 | 5 | 25 | 0.125 | Insect Damaged | -0.035 | -0.059 | 0.219 | 0.141 | 0.641 |
| 283 | Insect Damaged | 264.900 | 200 | 2119.200 | 5 | 25 | 0.125 | Good | -1.988 | -0.156 | 0.811 | 0.001 | 0.188 |
| 284 | Insect Damaged | 296.650 | 200 | 2373.200 | 5 | 25 | 0.125 | Insect Damaged | 0.753 | -0.020 | 0.058 | 0.470 | 0.472 |
| 285 | Insect Damaged | 268.830 | 200 | 2150.640 | 5 | 25 | 0.125 | Good | -1.649 | -0.139 | 0.733 | 0.003 | 0.264 |
| 286 | Insect Damaged | 272.790 | 200 | 2182.320 | 5 | 25 | 0.125 | Good | -1.307 | -0.122 | 0.636 | 0.007 | 0.357 |
| 287 | Insect Damaged | 277.670 | 200 | 2221.360 | 5 | 25 | 0.125 | Good | -0.886 | -0.101 | 0.497 | 0.021 | 0.482 |
| 288 | Insect Damaged | 272.750 | 200 | 2182.000 | 5 | 25 | 0.125 | Good | -1.311 | -0.122 | 0.637 | 0.007 | 0.356 |
| 289 | Insect Damaged | 286.900 | 200 | 2295.200 | 5 | 25 | 0.125 | Insect Damaged | -0.089 | -0.062 | 0.234 | 0.127 | 0.639 |
| 290 | Insect Damaged | 267.540 | 200 | 2140.320 | 5 | 25 | 0.125 | Good | -1.760 | -0.144 | 0.761 | 0.002 | 0.237 |
| 291 | Insect Damaged | 284.620 | 200 | 2276.960 | 5 | 25 | 0.125 | Insect Damaged | -0.286 | -0.071 | 0.294 | 0.084 | 0.622 |
| 292 | Insect Damaged | 287.800 | 200 | 2302.200 | 5 | 25 | 0.125 | Insect Damaged | -0.011 | -0.058 | 0.212 | 0.147 | 0.641 |
| 293 | Insect Damaged | 290.790 | 200 | 2326.320 | 5 | 25 | 0.125 | Insect Damaged | 0.247 | -0.045 | 0.147 | 0.234 | 0.619 |
| 294 | Insect Damaged | 286.840 | 200 | 2294.720 | 5 | 25 | 0.125 | Insect Damaged | -0.094 | -0.062 | 0.236 | 0.125 | 0.639 |
| 295 | Insect Damaged | 254.850 | 200 | 2038.800 | 5 | 25 | 0.125 | Good | -2.856 | -0.198 | 0.930 | 0.000 | 0.070 |
| 296 | Insect Damaged | 286.850 | 200 | 2294.800 | 5 | 25 | 0.125 | Insect Damaged | -0.093 | -0.062 | 0.235 | 0.126 | 0.639 |
| 297 | Insect Damaged | 283.690 | 200 | 2269.520 | 5 | 25 | 0.125 | Insect Damaged | -0.366 | -0.075 | 0.320 | 0.071 | 0.609 |
| 298 | Insect Damaged | 289.780 | 200 | 2318.240 | 5 | 25 | 0.125 | Insect Damaged | 0.160 | -0.050 | 0.167 | 0.202 | 0.631 |
| 299 | Insect Damaged | 290.330 | 200 | 2322.640 | 5 | 25 | 0.125 | Insect Damaged | 0.207 | -0.047 | 0.156 | 0.219 | 0.625 |
| 300 | Insect Damaged | 289.740 | 200 | 2317.920 | 5 | 25 | 0.125 | Insect Damaged | 0.156 | -0.050 | 0.168 | 0.200 | 0.632 |

A = Amplitude, F = Frequency, I = Intensity, T = Period, V = Velocity, ʎ = Wavelength,

‘Pred. Yam quality’ is the predicted yam quality class.

Quality class: 1= Good, 2 = Diseased damaged and 3 = Insect damaged

‘DSF1’ is ‘Discriminant Scores from Function (Model) 1’.

‘DSF2’ is ‘Discriminant Scores from Function (Model) 2’,

‘PMC1’ is the ‘Probabilities of Membership in Yam Quality Class 1’.

‘PMC2’ is the ‘Probabilities of Membership in Yam Quality Class 2’.

‘PMC3’ is the ‘Probabilities of Membership in Yam Quality Class 3’

Table S4: Acoustic properties of white yam qualities for surface impact sound technique and their classification training result using machine learning algorithm

| SN | Yam quality | A | F | I | T | V | ʎ | Pred. Yam quality | DSF1 | DSF2 | PMC1 | PMC2 | PMC3 |
| --- | --- | --- | --- | --- | --- | --- | --- | --- | --- | --- | --- | --- | --- |
| 1 | Good | 155.470 | 166.970 | 1619.470 | 3 | 16 | 0.090 | Good | 3.837 | 0.744 | 0.590 | 0 | 0.410 |
| 2 | Good | 143.720 | 154.320 | 1381.920 | 2 | 16 | 0.100 | Insect Damaged | 3.188 | -0.544 | 0.450 | 0 | 0.550 |
| 3 | Good | 142.660 | 153.650 | 1371.730 | 2 | 16 | 0.100 | Insect Damaged | 3.095 | -0.550 | 0.445 | 0 | 0.555 |
| 4 | Good | 155.490 | 166.540 | 1619.680 | 3 | 16 | 0.090 | Good | 3.782 | 0.780 | 0.590 | 0 | 0.410 |
| 5 | Good | 153.650 | 164.460 | 1584.020 | 3 | 16 | 0.090 | Good | 3.495 | 0.766 | 0.576 | 0 | 0.424 |
| 6 | Good | 144.410 | 155.430 | 1402.030 | 2 | 16 | 0.100 | Insect Damaged | 3.340 | -0.536 | 0.458 | 0 | 0.542 |
| 7 | Good | 155.680 | 166.130 | 1621.660 | 3 | 16 | 0.090 | Good | 3.730 | 0.824 | 0.591 | 0 | 0.409 |
| 8 | Good | 131.730 | 142.780 | 1176.160 | 2 | 16 | 0.110 | Insect Damaged | 2.420 | -0.362 | 0.428 | 0 | 0.572 |
| 9 | Good | 141.820 | 152.640 | 1350.660 | 2 | 16 | 0.100 | Insect Damaged | 2.954 | -0.572 | 0.436 | 0 | 0.564 |
| 10 | Good | 137.840 | 148.670 | 1276.290 | 2 | 16 | 0.110 | Insect Damaged | 3.233 | -0.312 | 0.472 | 0 | 0.528 |
| 11 | Good | 156.720 | 167.430 | 1649.680 | 3 | 16 | 0.090 | Good | 3.910 | 0.857 | 0.603 | 0 | 0.397 |
| 12 | Good | 142.830 | 153.610 | 1373.360 | 2 | 16 | 0.100 | Insect Damaged | 3.091 | -0.537 | 0.446 | 0 | 0.554 |
| 13 | Good | 160.370 | 171.320 | 1724.400 | 3 | 16 | 0.090 | Good | 4.450 | 0.921 | 0.632 | 0 | 0.368 |
| 14 | Good | 158.340 | 169.540 | 1702.580 | 3 | 16 | 0.090 | Good | 4.206 | 0.940 | 0.623 | 0 | 0.377 |
| 15 | Good | 135.830 | 146.430 | 1246.140 | 2 | 16 | 0.110 | Insect Damaged | 2.927 | -0.292 | 0.458 | 0 | 0.542 |
| 16 | Good | 135.320 | 146.750 | 1241.460 | 2 | 16 | 0.110 | Insect Damaged | 2.966 | -0.346 | 0.456 | 0 | 0.544 |
| 17 | Good | 135.760 | 146.810 | 1245.500 | 2 | 16 | 0.110 | Insect Damaged | 2.976 | -0.327 | 0.458 | 0 | 0.542 |
| 18 | Good | 152.430 | 163.720 | 1555.400 | 3 | 16 | 0.090 | Good | 3.386 | 0.683 | 0.563 | 0 | 0.437 |
| 19 | Good | 145.870 | 156.760 | 1430.090 | 2 | 16 | 0.100 | Insect Damaged | 3.526 | -0.500 | 0.470 | 0 | 0.530 |
| 20 | Good | 132.540 | 143.650 | 1194.050 | 2 | 16 | 0.110 | Insect Damaged | 2.541 | -0.343 | 0.436 | 0 | 0.564 |
| 21 | Good | 158.450 | 169.430 | 1685.630 | 3 | 16 | 0.090 | Good | 4.186 | 0.877 | 0.617 | 0 | 0.383 |
| 22 | Good | 148.530 | 156.420 | 1456.170 | 2 | 16 | 0.100 | Insect Damaged | 3.497 | -0.320 | 0.484 | 0 | 0.516 |
| 23 | Good | 131.760 | 142.350 | 1176.420 | 2 | 16 | 0.110 | Insect Damaged | 2.364 | -0.326 | 0.429 | 0 | 0.571 |
| 24 | Good | 160.760 | 171.350 | 1728.600 | 3 | 16 | 0.090 | Good | 4.456 | 0.943 | 0.634 | 0 | 0.366 |
| 25 | Good | 142.830 | 153.760 | 1373.360 | 2 | 16 | 0.100 | Insect Damaged | 3.110 | -0.549 | 0.446 | 0 | 0.554 |
| 26 | Good | 142.450 | 153.870 | 1369.710 | 2 | 16 | 0.100 | Insect Damaged | 3.122 | -0.580 | 0.444 | 0 | 0.556 |
| 27 | Good | 153.750 | 164.730 | 1585.050 | 3 | 16 | 0.090 | Good | 3.531 | 0.750 | 0.576 | 0 | 0.424 |
| 28 | Good | 143.320 | 153.730 | 1378.070 | 2 | 16 | 0.100 | Insect Damaged | 3.109 | -0.519 | 0.448 | 0 | 0.552 |
| 29 | Good | 146.980 | 157.960 | 1455.240 | 2 | 16 | 0.100 | Insect Damaged | 3.693 | -0.471 | 0.480 | 0 | 0.520 |
| 30 | Good | 140.630 | 151.490 | 1326.690 | 2 | 16 | 0.110 | Insect Damaged | 3.623 | -0.280 | 0.493 | 0 | 0.507 |
| 31 | Good | 151.730 | 162.840 | 1548.260 | 3 | 16 | 0.090 | Good | 3.268 | 0.713 | 0.560 | 0 | 0.440 |
| 32 | Good | 152.540 | 163.280 | 1556.530 | 3 | 16 | 0.090 | Good | 3.330 | 0.725 | 0.564 | 0 | 0.436 |
| 33 | Good | 136.750 | 147.790 | 1266.200 | 2 | 16 | 0.110 | Insect Damaged | 3.113 | -0.301 | 0.467 | 0 | 0.533 |
| 34 | Good | 161.960 | 172.640 | 1741.500 | 3 | 16 | 0.090 | Good | 4.630 | 0.912 | 0.640 | 0 | 0.360 |
| 35 | Good | 143.230 | 154.820 | 1390.580 | 2 | 16 | 0.100 | Insect Damaged | 3.254 | -0.554 | 0.452 | 0 | 0.548 |
| 36 | Good | 147.810 | 158.360 | 1463.460 | 2 | 16 | 0.100 | Insect Damaged | 3.750 | -0.456 | 0.484 | 0 | 0.516 |
| 37 | Good | 223.510 | 163.730 | 2280.710 | 3 | 16 | 0.090 | Good | 3.811 | 4.893 | 0.855 | 0 | 0.145 |
| 38 | Good | 223.540 | 145.770 | 2050.820 | 2 | 16 | 0.110 | Good | 3.332 | 4.565 | 0.828 | 0 | 0.172 |
| 39 | Good | 273.740 | 148.760 | 2558.310 | 2 | 16 | 0.110 | Good | 4.017 | 7.276 | 0.933 | 0 | 0.067 |
| 40 | Good | 161.760 | 172.380 | 1758.260 | 3 | 16 | 0.090 | Good | 4.602 | 1.003 | 0.646 | 0 | 0.354 |
| 41 | Good | 154.830 | 165.430 | 1612.810 | 3 | 16 | 0.090 | Good | 3.634 | 0.830 | 0.587 | 0 | 0.413 |
| 42 | Good | 136.190 | 147.320 | 1261.010 | 2 | 16 | 0.110 | Insect Damaged | 3.049 | -0.294 | 0.464 | 0 | 0.536 |
| 43 | Good | 141.390 | 152.940 | 1470.570 | 2 | 16 | 0.100 | Insect Damaged | 3.035 | -0.083 | 0.481 | 0 | 0.519 |
| 44 | Good | 145.210 | 156.470 | 1423.620 | 2 | 16 | 0.100 | Insect Damaged | 3.485 | -0.515 | 0.467 | 0 | 0.534 |
| 45 | Good | 140.840 | 151.470 | 1341.330 | 2 | 16 | 0.110 | Insect Damaged | 3.626 | -0.211 | 0.499 | 0 | 0.501 |
| 46 | Good | 161.240 | 172.320 | 1752.600 | 3 | 16 | 0.090 | Good | 4.591 | 0.975 | 0.643 | 0 | 0.357 |
| 47 | Good | 134.850 | 145.450 | 1225.900 | 2 | 16 | 0.110 | Insect Damaged | 2.791 | -0.315 | 0.450 | 0 | 0.550 |
| 48 | Good | 156.110 | 167.630 | 800.560 | 2 | 16 | 0.190 | Good | 12.192 | -1.062 | 0.797 | 0 | 0.203 |
| 49 | Good | 144.840 | 155.450 | 1420.000 | 2 | 16 | 0.100 | Insect Damaged | 3.350 | -0.453 | 0.465 | 0 | 0.535 |
| 50 | Good | 132.950 | 143.580 | 1197.740 | 2 | 16 | 0.110 | Insect Damaged | 2.534 | -0.315 | 0.438 | 0 | 0.562 |
| 51 | Good | 140.490 | 151.460 | 1338.000 | 2 | 16 | 0.110 | Insect Damaged | 3.622 | -0.230 | 0.497 | 0 | 0.503 |
| 52 | Good | 154.870 | 165.420 | 1613.220 | 2 | 16 | 0.090 | Insect Damaged | 3.906 | -0.592 | 0.480 | 0 | 0.520 |
| 53 | Good | 142.760 | 153.710 | 1372.690 | 2 | 16 | 0.100 | Insect Damaged | 3.103 | -0.549 | 0.445 | 0 | 0.555 |
| 54 | Good | 135.740 | 146.360 | 1245.320 | 2 | 16 | 0.110 | Insect Damaged | 2.918 | -0.291 | 0.458 | 0 | 0.542 |
| 55 | Good | 142.890 | 153.760 | 1373.940 | 2 | 16 | 0.100 | Insect Damaged | 3.110 | -0.546 | 0.446 | 0 | 0.554 |
| 56 | Good | 135.850 | 146.820 | 1257.870 | 2 | 16 | 0.110 | Insect Damaged | 2.982 | -0.272 | 0.463 | 0 | 0.537 |
| 57 | Good | 146.850 | 157.350 | 1453.960 | 2 | 16 | 0.100 | Insect Damaged | 3.613 | -0.430 | 0.480 | 0 | 0.520 |
| 58 | Good | 138.830 | 149.530 | 1297.470 | 2 | 16 | 0.110 | Insect Damaged | 3.354 | -0.275 | 0.481 | 0 | 0.519 |
| 59 | Good | 138.590 | 149.560 | 1307.450 | 2 | 16 | 0.110 | Insect Damaged | 3.361 | -0.238 | 0.484 | 0 | 0.516 |
| 60 | Good | 152.970 | 163.740 | 1577.010 | 3 | 16 | 0.090 | Good | 3.398 | 0.783 | 0.572 | 0 | 0.428 |
| 61 | Good | 168.430 | 179.360 | 1892.470 | 3 | 16 | 0.080 | Good | 4.741 | 0.799 | 0.636 | 0 | 0.364 |
| 62 | Good | 146.950 | 157.350 | 1454.950 | 2 | 16 | 0.100 | Insect Damaged | 3.614 | -0.424 | 0.480 | 0 | 0.520 |
| 63 | Good | 131.740 | 142.670 | 1176.250 | 2 | 16 | 0.110 | Insect Damaged | 2.406 | -0.353 | 0.428 | 0 | 0.572 |
| 64 | Good | 167.650 | 178.630 | 1883.700 | 3 | 16 | 0.080 | Good | 4.642 | 0.809 | 0.632 | 0 | 0.368 |
| 65 | Good | 133.380 | 144.830 | 1212.540 | 2 | 16 | 0.110 | Insect Damaged | 2.702 | -0.345 | 0.443 | 0 | 0.557 |
| 66 | Good | 135.750 | 146.730 | 1245.410 | 2 | 16 | 0.110 | Insect Damaged | 2.966 | -0.321 | 0.458 | 0 | 0.542 |
| 67 | Good | 157.910 | 168.480 | 1679.890 | 3 | 16 | 0.090 | Good | 4.060 | 0.921 | 0.615 | 0 | 0.385 |
| 68 | Good | 142.840 | 153.760 | 1373.460 | 2 | 16 | 0.100 | Insect Damaged | 3.110 | -0.549 | 0.446 | 0 | 0.554 |
| 69 | Good | 142.730 | 153.640 | 1372.400 | 2 | 16 | 0.100 | Insect Damaged | 3.094 | -0.545 | 0.445 | 0 | 0.555 |
| 70 | Good | 160.860 | 171.210 | 1729.670 | 3 | 16 | 0.090 | Good | 4.438 | 0.960 | 0.635 | 0 | 0.365 |
| 71 | Good | 135.940 | 146.850 | 1258.700 | 2 | 16 | 0.110 | Insect Damaged | 2.986 | -0.270 | 0.463 | 0 | 0.537 |
| 72 | Good | 151.830 | 162.760 | 1549.280 | 3 | 16 | 0.090 | Good | 3.258 | 0.725 | 0.561 | 0 | 0.439 |
| 73 | Good | 142.220 | 153.120 | 1367.500 | 2 | 16 | 0.100 | Insect Damaged | 3.024 | -0.532 | 0.443 | 0 | 0.557 |
| 74 | Good | 0.000 | 167.340 | 1650.000 | 3 | 16 | 0.090 | Insect Damaged | 3.533 | -1.482 | 0.388 | 0 | 0.612 |
| 75 | Good | 143.710 | 154.820 | 1395.240 | 2 | 16 | 0.100 | Insect Damaged | 3.257 | -0.527 | 0.455 | 0 | 0.546 |
| 76 | Good | 147.850 | 158.360 | 1463.860 | 2 | 16 | 0.100 | Insect Damaged | 3.750 | -0.453 | 0.485 | 0 | 0.515 |
| 77 | Good | 152.950 | 163.730 | 1576.800 | 3 | 16 | 0.090 | Good | 3.396 | 0.783 | 0.572 | 0 | 0.428 |
| 78 | Good | 134.880 | 145.770 | 1215.130 | 2 | 16 | 0.110 | Insect Damaged | 2.828 | -0.387 | 0.446 | 0 | 0.554 |
| 79 | Good | 137.450 | 148.760 | 1284.570 | 2 | 16 | 0.100 | Insect Damaged | 2.418 | -0.611 | 0.408 | 0 | 0.592 |
| 80 | Good | 131.540 | 142.380 | 1174.460 | 2 | 16 | 0.110 | Insect Damaged | 2.367 | -0.340 | 0.428 | 0 | 0.572 |
| 81 | Good | 131.860 | 142.670 | 1177.320 | 2 | 16 | 0.110 | Insect Damaged | 2.406 | -0.346 | 0.429 | 0 | 0.571 |
| 82 | Good | 147.760 | 158.630 | 1462.970 | 3 | 16 | 0.100 | Good | 3.511 | 0.945 | 0.591 | 0 | 0.409 |
| 83 | Good | 264.780 | 144.830 | 1316.630 | 2 | 16 | 0.110 | Good | 3.046 | 2.074 | 0.660 | 0 | 0.340 |
| 84 | Good | 143.540 | 154.730 | 1393.590 | 2 | 16 | 0.100 | Insect Damaged | 3.244 | -0.529 | 0.454 | 0 | 0.546 |
| 85 | Good | 137.730 | 148.670 | 1287.190 | 2 | 16 | 0.100 | Insect Damaged | 2.408 | -0.588 | 0.409 | 0 | 0.591 |
| 86 | Good | 158.750 | 167.430 | 1671.050 | 3 | 16 | 0.090 | Good | 3.923 | 0.980 | 0.613 | 0 | 0.387 |
| 87 | Good | 142.750 | 153.610 | 1372.590 | 2 | 16 | 0.100 | Insect Damaged | 3.090 | -0.542 | 0.445 | 0 | 0.555 |
| 88 | Good | 150.590 | 161.320 | 1521.110 | 3 | 16 | 0.090 | Good | 3.059 | 0.701 | 0.549 | 0 | 0.451 |
| 89 | Good | 135.380 | 146.850 | 1242.010 | 2 | 16 | 0.110 | Insect Damaged | 2.979 | -0.351 | 0.456 | 0 | 0.544 |
| 90 | Good | 151.530 | 162.760 | 1546.220 | 3 | 16 | 0.090 | Good | 3.256 | 0.707 | 0.559 | 0 | 0.441 |
| 91 | Good | 156.530 | 167.340 | 1647.680 | 3 | 16 | 0.090 | Good | 3.898 | 0.852 | 0.602 | 0 | 0.398 |
| 92 | Good | 135.840 | 146.660 | 1246.230 | 2 | 16 | 0.110 | Insect Damaged | 2.957 | -0.310 | 0.458 | 0 | 0.542 |
| 93 | Good | 143.640 | 154.320 | 1394.560 | 2 | 16 | 0.100 | Insect Damaged | 3.192 | -0.490 | 0.454 | 0 | 0.546 |
| 94 | Good | 142.850 | 153.650 | 1373.550 | 2 | 16 | 0.100 | Insect Damaged | 3.096 | -0.539 | 0.446 | 0 | 0.554 |
| 95 | Good | 155.940 | 166.540 | 1624.370 | 3 | 16 | 0.090 | Good | 3.784 | 0.807 | 0.593 | 0 | 0.407 |
| 96 | Good | 153.640 | 164.460 | 1583.910 | 3 | 16 | 0.090 | Good | 3.495 | 0.765 | 0.575 | 0 | 0.425 |
| 97 | Good | 144.880 | 155.430 | 1420.390 | 2 | 16 | 0.120 | Good | 5.005 | 0.194 | 0.599 | 0 | 0.401 |
| 98 | Good | 136.850 | 147.380 | 1267.120 | 2 | 16 | 0.110 | Insect Damaged | 3.060 | -0.263 | 0.467 | 0 | 0.533 |
| 99 | Good | 136.350 | 147.640 | 1262.500 | 2 | 16 | 0.110 | Insect Damaged | 3.091 | -0.311 | 0.465 | 0 | 0.535 |
| 100 | Good | 76.720 | 87.130 | 833.910 | 1 | 16 | 0.090 | Disease damaged | -6.425 | -0.242 | 0.000 | 1 | 0 |
| 101 | Disease damaged | 61.840 | 72.780 | 567.330 | 1 | 8 | 0.110 | Disease damaged | -6.757 | 0.182 | 0.000 | 1 | 0 |
| 102 | Disease damaged | 61.220 | 72.640 | 556.540 | 1 | 8 | 0.110 | Disease damaged | -6.780 | 0.137 | 0.000 | 1 | 0 |
| 103 | Disease damaged | 67.860 | 78.670 | 671.880 | 1 | 8 | 0.100 | Disease damaged | -6.771 | -0.072 | 0.000 | 1 | 0 |
| 104 | Disease damaged | 60.850 | 71.430 | 548.190 | 1 | 8 | 0.110 | Disease damaged | -6.941 | 0.193 | 0.000 | 1 | 0 |
| 105 | Disease damaged | 83.120 | 94.610 | 989.520 | 1 | 8 | 0.080 | Disease damaged | -6.214 | -0.397 | 0.000 | 1 | 0 |
| 106 | Disease damaged | 60.840 | 71.320 | 543.210 | 1 | 8 | 0.110 | Disease damaged | -6.957 | 0.180 | 0.000 | 1 | 0 |
| 107 | Disease damaged | 68.960 | 79.540 | 689.600 | 1 | 8 | 0.100 | Disease damaged | -6.650 | -0.049 | 0.000 | 1 | 0 |
| 108 | Disease damaged | 75.220 | 86.430 | 817.600 | 1 | 8 | 0.090 | Disease damaged | -6.525 | -0.279 | 0.000 | 1 | 0 |
| 109 | Disease damaged | 85.840 | 96.750 | 1046.800 | 1 | 8 | 0.080 | Disease damaged | -5.910 | -0.281 | 0.000 | 1 | 0 |
| 110 | Disease damaged | 65.860 | 76.810 | 633.260 | 1 | 8 | 0.100 | Disease damaged | -7.031 | -0.120 | 0.000 | 1 | 0 |
| 111 | Disease damaged | 82.840 | 93.720 | 974.580 | 1 | 8 | 0.080 | Disease damaged | -6.336 | -0.394 | 0.000 | 1 | 0 |
| 112 | Disease damaged | 75.850 | 86.760 | 824.460 | 1 | 8 | 0.090 | Disease damaged | -6.479 | -0.266 | 0.000 | 1 | 0 |
| 113 | Disease damaged | 77.960 | 88.630 | 866.220 | 1 | 8 | 0.090 | Disease damaged | -6.217 | -0.205 | 0.000 | 1 | 0 |
| 114 | Disease damaged | 60.720 | 71.460 | 547.020 | 1 | 8 | 0.110 | Disease damaged | -6.938 | 0.183 | 0.000 | 1 | 0 |
| 115 | Disease damaged | 62.940 | 73.830 | 582.770 | 1 | 8 | 0.100 | Disease damaged | -7.441 | -0.141 | 0.000 | 1 | 0 |
| 116 | Disease damaged | 83.650 | 94.820 | 995.830 | 1 | 8 | 0.080 | Disease damaged | -6.184 | -0.379 | 0.000 | 1 | 0 |
| 117 | Disease damaged | 87.340 | 98.360 | 1078.200 | 2 | 8 | 0.080 | Disease damaged | -5.960 | 1.173 | 0.000 | 1 | 0 |
| 118 | Disease damaged | 62.740 | 73.730 | 580.920 | 1 | 8 | 0.100 | Disease damaged | -7.456 | -0.144 | 0.000 | 1 | 0 |
| 119 | Disease damaged | 74.870 | 85.770 | 805.050 | 1 | 8 | 0.090 | Disease damaged | -6.616 | -0.285 | 0.000 | 1 | 0 |
| 120 | Disease damaged | 77.960 | 88.760 | 866.220 | 1 | 8 | 0.090 | Disease damaged | -6.200 | -0.215 | 0.000 | 1 | 0 |
| 121 | Disease damaged | 61.850 | 72.380 | 562.270 | 1 | 8 | 0.110 | Disease damaged | -6.811 | 0.192 | 0.000 | 1 | 0 |
| 122 | Disease damaged | 74.850 | 85.430 | 718.920 | 1 | 8 | 0.090 | Disease damaged | -6.691 | -0.631 | 0.000 | 1 | 0 |
| 123 | Disease damaged | 66.860 | 77.320 | 649.120 | 1 | 8 | 0.100 | Disease damaged | -6.957 | -0.077 | 0.000 | 1 | 0 |
| 124 | Disease damaged | 81.840 | 92.940 | 951.620 | 1 | 8 | 0.080 | Disease damaged | -6.447 | -0.446 | 0.000 | 1 | 0 |
| 125 | Disease damaged | 65.540 | 76.470 | 630.190 | 1 | 8 | 0.100 | Disease damaged | -7.076 | -0.110 | 0.000 | 1 | 0 |
| 126 | Disease damaged | 70.240 | 81.470 | 716.730 | 1 | 8 | 0.090 | Disease damaged | -7.215 | -0.390 | 0.000 | 1 | 0 |
| 127 | Disease damaged | 61.170 | 72.320 | 556.090 | 1 | 8 | 0.110 | Disease damaged | -6.822 | 0.160 | 0.000 | 1 | 0 |
| 128 | Disease damaged | 84.940 | 95.450 | 1023.300 | 1 | 8 | 0.080 | Disease damaged | -6.089 | -0.291 | 0.000 | 1 | 0 |
| 129 | Disease damaged | 86.890 | 97.630 | 1072.700 | 1 | 8 | 0.080 | Disease damaged | -5.785 | -0.224 | 0.000 | 1 | 0 |
| 130 | Disease damaged | 74.780 | 85.450 | 804.080 | 1 | 8 | 0.090 | Disease damaged | -6.658 | -0.265 | 0.000 | 1 | 0 |
| 131 | Disease damaged | 62.950 | 73.580 | 582.870 | 1 | 8 | 0.100 | Disease damaged | -7.474 | -0.121 | 0.000 | 1 | 0 |
| 132 | Disease damaged | 80.340 | 91.460 | 923.440 | 1 | 8 | 0.080 | Disease damaged | -6.653 | -0.471 | 0.000 | 1 | 0 |
| 133 | Disease damaged | 57.860 | 68.420 | 489.790 | 1 | 8 | 0.110 | Disease damaged | -7.359 | 0.138 | 0.000 | 1 | 0 |
| 134 | Disease damaged | 62.850 | 73.710 | 581.940 | 1 | 8 | 0.110 | Disease damaged | -6.629 | 0.185 | 0.000 | 1 | 0 |
| 135 | Disease damaged | 65.880 | 76.360 | 633.460 | 1 | 8 | 0.100 | Disease damaged | -7.089 | -0.082 | 0.000 | 1 | 0 |
| 136 | Disease damaged | 74.230 | 85.990 | 798.170 | 1 | 8 | 0.090 | Disease damaged | -6.591 | -0.342 | 0.000 | 1 | 0 |
| 137 | Disease damaged | 65.740 | 76.430 | 632.110 | 1 | 8 | 0.100 | Disease damaged | -7.080 | -0.096 | 0.000 | 1 | 0 |
| 138 | Disease damaged | 87.950 | 98.530 | 1085.800 | 1 | 8 | 0.080 | Disease damaged | -5.661 | -0.224 | 0.000 | 1 | 0 |
| 139 | Disease damaged | 60.490 | 71.280 | 540.080 | 1 | 8 | 0.110 | Disease damaged | -6.964 | 0.164 | 0.000 | 1 | 0 |
| 140 | Disease damaged | 82.960 | 93.650 | 976.000 | 1 | 8 | 0.090 | Disease damaged | -5.515 | -0.059 | 0.000 | 1 | 0 |
| 141 | Disease damaged | 76.640 | 87.460 | 842.190 | 1 | 8 | 0.090 | Disease damaged | -6.380 | -0.234 | 0.000 | 1 | 0 |
| 142 | Disease damaged | 63.640 | 74.980 | 600.370 | 1 | 8 | 0.110 | Disease damaged | -6.456 | 0.174 | 0.000 | 1 | 0 |
| 143 | Disease damaged | 77.590 | 88.810 | 862.110 | 1 | 8 | 0.090 | Disease damaged | -6.196 | -0.242 | 0.000 | 1 | 0 |
| 144 | Disease damaged | 82.980 | 93.640 | 976.230 | 1 | 8 | 0.090 | Disease damaged | -5.517 | -0.057 | 0.000 | 1 | 0 |
| 145 | Disease damaged | 83.970 | 94.410 | 999.640 | 1 | 8 | 0.080 | Disease damaged | -6.235 | -0.324 | 0.000 | 1 | 0 |
| 146 | Disease damaged | 85.980 | 96.970 | 1048.500 | 1 | 8 | 0.080 | Disease damaged | -5.881 | -0.289 | 0.000 | 1 | 0 |
| 147 | Disease damaged | 63.980 | 74.320 | 597.940 | 1 | 8 | 0.110 | Disease damaged | -6.542 | 0.222 | 0.000 | 1 | 0 |
| 148 | Disease damaged | 62.880 | 73.650 | 582.220 | 1 | 8 | 0.110 | Disease damaged | -6.637 | 0.191 | 0.000 | 1 | 0 |
| 149 | Disease damaged | 81.750 | 92.540 | 950.580 | 1 | 8 | 0.090 | Disease damaged | -5.671 | -0.098 | 0.000 | 1 | 0 |
| 150 | Disease damaged | 73.840 | 84.460 | 785.530 | 1 | 8 | 0.090 | Disease damaged | -6.795 | -0.279 | 0.000 | 1 | 0 |
| 151 | Disease damaged | 84.120 | 95.430 | 1013.400 | 1 | 8 | 0.080 | Disease damaged | -6.097 | -0.345 | 0.000 | 1 | 0 |
| 152 | Disease damaged | 65.450 | 76.130 | 623.330 | 1 | 8 | 0.110 | Disease damaged | -6.295 | 0.208 | 0.000 | 1 | 0 |
| 153 | Disease damaged | 81.210 | 92.780 | 944.300 | 1 | 8 | 0.080 | Disease damaged | -6.472 | -0.474 | 0.000 | 1 | 0 |
| 154 | Disease damaged | 61.430 | 72.640 | 0.000 | 1 | 8 | 0.110 | Disease damaged | -6.978 | -2.274 | 0.000 | 1 | 0 |
| 155 | Disease damaged | 73.850 | 84.670 | 785.630 | 1 | 8 | 0.090 | Disease damaged | -6.768 | -0.296 | 0.000 | 1 | 0 |
| 156 | Disease damaged | 66.740 | 77.430 | 647.960 | 1 | 8 | 0.100 | Disease damaged | -6.943 | -0.093 | 0.000 | 1 | 0 |
| 157 | Disease damaged | 72.650 | 83.610 | 764.730 | 1 | 8 | 0.090 | Disease damaged | -6.915 | -0.319 | 0.000 | 1 | 0 |
| 158 | Disease damaged | 60.170 | 71.320 | 501.410 | 1 | 8 | 0.110 | Disease damaged | -6.974 | -0.011 | 0.000 | 1 | 0 |
| 159 | Disease damaged | 88.680 | 99.540 | 1108.500 | 1 | 8 | 0.080 | Disease damaged | -5.520 | -0.196 | 0.000 | 1 | 0 |
| 160 | Disease damaged | 63.950 | 74.430 | 878.030 | 1 | 8 | 0.110 | Disease damaged | -6.428 | 1.427 | 0.000 | 1 | 0 |
| 161 | Disease damaged | 76.960 | 84.350 | 658.580 | 1 | 8 | 0.100 | Disease damaged | -6.019 | -0.453 | 0.000 | 1 | 0 |
| 162 | Disease damaged | 75.980 | 86.750 | 825.860 | 1 | 8 | 0.090 | Disease damaged | -6.479 | -0.257 | 0.000 | 1 | 0 |
| 163 | Disease damaged | 85.970 | 96.810 | 1048.400 | 1 | 8 | 0.080 | Disease damaged | -5.902 | -0.277 | 0.000 | 1 | 0 |
| 164 | Disease damaged | 62.640 | 73.720 | 580.000 | 1 | 8 | 0.100 | Disease damaged | -7.457 | -0.149 | 0.000 | 1 | 0 |
| 165 | Disease damaged | 65.220 | 76.760 | 627.110 | 1 | 8 | 0.100 | Disease damaged | -7.041 | -0.152 | 0.000 | 1 | 0 |
| 166 | Disease damaged | 62.890 | 73.650 | 582.310 | 1 | 8 | 0.110 | Disease damaged | -6.637 | 0.192 | 0.000 | 1 | 0 |
| 167 | Disease damaged | 88.640 | 99.430 | 1108.000 | 1 | 8 | 0.080 | Disease damaged | -5.535 | -0.190 | 0.000 | 1 | 0 |
| 168 | Disease damaged | 77.760 | 88.630 | 864.000 | 1 | 8 | 0.090 | Disease damaged | -6.218 | -0.217 | 0.000 | 1 | 0 |
| 169 | Disease damaged | 73.670 | 84.540 | 783.720 | 1 | 8 | 0.090 | Disease damaged | -6.786 | -0.296 | 0.000 | 1 | 0 |
| 170 | Disease damaged | 76.780 | 87.480 | 843.730 | 1 | 8 | 0.090 | Disease damaged | -6.376 | -0.227 | 0.000 | 1 | 0 |
| 171 | Disease damaged | 87.180 | 98.670 | 1076.200 | 1 | 8 | 0.080 | Disease damaged | -5.648 | -0.289 | 0.000 | 1 | 0 |
| 172 | Disease damaged | 84.360 | 95.430 | 1016.380 | 1 | 8 | 0.080 | Disease damaged | -6.096 | -0.328 | 0.000 | 1 | 0 |
| 173 | Disease damaged | 78.540 | 89.320 | 882.470 | 1 | 8 | 0.090 | Disease damaged | -6.120 | -0.181 | 0.000 | 1 | 0 |
| 174 | Disease damaged | 80.870 | 91.940 | 929.540 | 1 | 8 | 0.090 | Disease damaged | -5.758 | -0.154 | 0.000 | 1 | 0 |
| 175 | Disease damaged | 82.840 | 93.470 | 974.580 | 1 | 8 | 0.090 | Disease damaged | -5.540 | -0.053 | 0.000 | 1 | 0 |
| 176 | Disease damaged | 60.850 | 71.470 | 548.190 | 1 | 8 | 0.110 | Disease damaged | -6.936 | 0.190 | 0.000 | 1 | 0 |
| 177 | Disease damaged | 71.750 | 82.320 | 739.690 | 1 | 8 | 0.090 | Disease damaged | -7.094 | -0.336 | 0.000 | 1 | 0 |
| 178 | Disease damaged | 75.650 | 85.450 | 813.440 | 1 | 8 | 0.090 | Disease damaged | -6.653 | -0.211 | 0.000 | 1 | 0 |
| 179 | Disease damaged | 76.850 | 87.630 | 844.500 | 1 | 8 | 0.090 | Disease damaged | -6.356 | -0.235 | 0.000 | 1 | 0 |
| 180 | Disease damaged | 64.840 | 75.450 | 611.690 | 1 | 8 | 0.110 | Disease damaged | -6.388 | 0.203 | 0.000 | 1 | 0 |
| 181 | Disease damaged | 72.670 | 83.580 | 764.940 | 1 | 8 | 0.090 | Disease damaged | -6.919 | -0.315 | 0.000 | 1 | 0 |
| 182 | Disease damaged | 58.640 | 69.460 | 2932.000 | 1 | 8 | 0.050 | Disease damaged | -11.326 | 8.728 | 0.000 | 1 | 0 |
| 183 | Disease damaged | 66.840 | 77.430 | 648.930 | 1 | 8 | 0.100 | Disease damaged | -6.942 | -0.087 | 0.000 | 1 | 0 |
| 184 | Disease damaged | 62.860 | 73.610 | 582.030 | 1 | 8 | 0.110 | Disease damaged | -6.642 | 0.193 | 0.000 | 1 | 0 |
| 185 | Disease damaged | 60.670 | 71.320 | 541.690 | 1 | 8 | 0.110 | Disease damaged | -6.958 | 0.171 | 0.000 | 1 | 0 |
| 186 | Disease damaged | 75.580 | 86.850 | 821.520 | 1 | 8 | 0.090 | Disease damaged | -6.469 | -0.290 | 0.000 | 1 | 0 |
| 187 | Disease damaged | 81.990 | 92.760 | 891.190 | 1 | 8 | 0.090 | Disease damaged | -5.663 | -0.370 | 0.000 | 1 | 0 |
| 188 | Disease damaged | 62.560 | 73.120 | 573.940 | 1 | 8 | 0.110 | Disease damaged | -6.709 | 0.193 | 0.000 | 1 | 0 |
| 189 | Disease damaged | 86.560 | 97.340 | 1055.600 | 1 | 8 | 0.080 | Disease damaged | -5.829 | -0.280 | 0.000 | 1 | 0 |
| 190 | Disease damaged | 72.850 | 83.650 | 766.840 | 1 | 8 | 0.090 | Disease damaged | -6.909 | -0.310 | 0.000 | 1 | 0 |
| 191 | Disease damaged | 85.730 | 96.540 | 1945.400 | 1 | 8 | 0.080 | Disease damaged | -5.619 | 3.632 | 0.000 | 1 | 0 |
| 192 | Disease damaged | 66.210 | 77.640 | 642.810 | 1 | 8 | 0.100 | Disease damaged | -6.919 | -0.140 | 0.000 | 1 | 0 |
| 193 | Disease damaged | 83.750 | 94.820 | 997.020 | 1 | 8 | 0.080 | Disease damaged | -6.183 | -0.372 | 0.000 | 1 | 0 |
| 194 | Disease damaged | 77.560 | 88.360 | 861.770 | 1 | 8 | 0.090 | Disease damaged | -6.254 | -0.208 | 0.000 | 1 | 0 |
| 195 | Disease damaged | 62.130 | 73.730 | 575.270 | 1 | 8 | 0.110 | Disease damaged | -6.630 | 0.144 | 0.000 | 1 | 0 |
| 196 | Disease damaged | 64.450 | 75.770 | 613.800 | 1 | 8 | 0.110 | Disease damaged | -6.347 | 0.181 | 0.000 | 1 | 0 |
| 197 | Disease damaged | 87.450 | 98.760 | 1079.600 | 1 | 8 | 0.080 | Disease damaged | -5.635 | -0.277 | 0.000 | 1 | 0 |
| 198 | Disease damaged | 61.840 | 72.380 | 562.180 | 1 | 8 | 0.110 | Disease damaged | -6.811 | 0.192 | 0.000 | 1 | 0 |
| 199 | Disease damaged | 71.760 | 82.670 | 747.500 | 1 | 8 | 0.090 | Disease damaged | -7.045 | -0.331 | 0.000 | 1 | 0 |
| 200 | Disease damaged | 78.130 | 68.630 | 498.700 | 1 | 8 | 0.120 | Disease damaged | -6.453 | 0.785 | 0.000 | 1 | 0 |
| 201 | Insect Damaged | 135.110 | 146.420 | 1239.540 | 2 | 16 | 0.110 | Insect Damaged | 2.922 | -0.331 | 0.455 | 0 | 0.545 |
| 202 | Insect Damaged | 131.860 | 142.350 | 1177.320 | 2 | 16 | 0.110 | Insect Damaged | 2.365 | -0.320 | 0.429 | 0 | 0.571 |
| 203 | Insect Damaged | 140.630 | 151.350 | 1339.330 | 2 | 16 | 0.110 | Insect Damaged | 3.609 | -0.214 | 0.498 | 0 | 0.502 |
| 204 | Insect Damaged | 132.760 | 143.760 | 1196.030 | 2 | 16 | 0.110 | Insect Damaged | 2.556 | -0.340 | 0.437 | 0 | 0.563 |
| 205 | Insect Damaged | 142.640 | 153.870 | 1384.850 | 2 | 16 | 0.100 | Insect Damaged | 3.128 | -0.511 | 0.450 | 0 | 0.550 |
| 206 | Insect Damaged | 153.220 | 164.730 | 1579.580 | 3 | 16 | 0.090 | Good | 3.527 | 0.718 | 0.573 | 0 | 0.427 |
| 207 | Insect Damaged | 152.980 | 163.730 | 1577.110 | 3 | 16 | 0.090 | Good | 3.396 | 0.785 | 0.572 | 0 | 0.428 |
| 208 | Insect Damaged | 146.730 | 157.960 | 1452.770 | 2 | 16 | 0.100 | Insect Damaged | 3.691 | -0.486 | 0.479 | 0 | 0.521 |
| 209 | Insect Damaged | 130.260 | 141.490 | 1289.700 | 2 | 16 | 0.110 | Insect Damaged | 2.290 | 0.213 | 0.471 | 0 | 0.530 |
| 210 | Insect Damaged | 151.630 | 162.840 | 1547.240 | 3 | 16 | 0.090 | Good | 3.267 | 0.707 | 0.560 | 0 | 0.440 |
| 111 | Insect Damaged | 157.870 | 154.630 | 1572.360 | 2 | 16 | 0.090 | Insect Damaged | 2.500 | 0.147 | 0.475 | 0 | 0.525 |
| 212 | Insect Damaged | 154.540 | 165.430 | 1609.790 | 3 | 16 | 0.090 | Good | 3.632 | 0.812 | 0.586 | 0 | 0.414 |
| 213 | Insect Damaged | 138.630 | 149.320 | 1295.600 | 2 | 16 | 0.110 | Insect Damaged | 3.326 | -0.269 | 0.480 | 0 | 0.520 |
| 214 | Insect Damaged | 140.630 | 151.940 | 1339.330 | 2 | 16 | 0.100 | Insect Damaged | 2.857 | -0.583 | 0.431 | 0 | 0.569 |
| 215 | Insect Damaged | 132.630 | 143.470 | 1194.860 | 2 | 16 | 0.110 | Insect Damaged | 2.518 | -0.323 | 0.436 | 0 | 0.564 |
| 216 | Insect Damaged | 140.650 | 151.470 | 1339.520 | 2 | 16 | 0.110 | Insect Damaged | 3.625 | -0.222 | 0.498 | 0 | 0.502 |
| 217 | Insect Damaged | 131.730 | 142.320 | 1176.160 | 2 | 16 | 0.110 | Insect Damaged | 2.360 | -0.325 | 0.429 | 0 | 0.571 |
| 218 | Insect Damaged | 144.940 | 155.450 | 1420.980 | 2 | 16 | 0.100 | Insect Damaged | 3.351 | -0.448 | 0.466 | 0 | 0.534 |
| 219 | Insect Damaged | 136.640 | 147.630 | 1265.180 | 2 | 16 | 0.100 | Insect Damaged | 2.263 | -0.616 | 0.400 | 0 | 0.600 |
| 220 | Insect Damaged | 144.750 | 155.450 | 1419.110 | 2 | 16 | 0.100 | Insect Damaged | 3.350 | -0.459 | 0.465 | 0 | 0.535 |
| 221 | Insect Damaged | 132.730 | 143.580 | 1195.760 | 2 | 16 | 0.110 | Insect Damaged | 2.533 | -0.327 | 0.437 | 0 | 0.563 |
| 222 | Insect Damaged | 150.840 | 161.460 | 1523.660 | 3 | 16 | 0.090 | Good | 3.078 | 0.704 | 0.550 | 0 | 0.450 |
| 223 | Insect Damaged | 146.960 | 157.430 | 1455.040 | 2 | 16 | 0.100 | Insect Damaged | 3.624 | -0.430 | 0.480 | 0 | 0.520 |
| 224 | Insect Damaged | 142.630 | 153.610 | 1371.440 | 2 | 16 | 0.100 | Insect Damaged | 3.089 | -0.548 | 0.445 | 0 | 0.555 |
| 225 | Insect Damaged | 130.670 | 141.320 | 1156.370 | 2 | 16 | 0.110 | Insect Damaged | 2.221 | -0.346 | 0.420 | 0 | 0.580 |
| 226 | Insect Damaged | 135.940 | 146.850 | 1258.700 | 2 | 16 | 0.110 | Insect Damaged | 2.986 | -0.270 | 0.463 | 0 | 0.537 |
| 227 | Insect Damaged | 151.680 | 162.760 | 1547.750 | 3 | 16 | 0.090 | Good | 3.257 | 0.716 | 0.560 | 0 | 0.440 |
| 228 | Insect Damaged | 142.750 | 153.120 | 1372.590 | 2 | 16 | 0.100 | Insect Damaged | 3.027 | -0.502 | 0.446 | 0 | 0.554 |
| 229 | Insect Damaged | 135.640 | 147.340 | 1255.920 | 2 | 16 | 0.110 | Insect Damaged | 3.048 | -0.326 | 0.461 | 0 | 0.539 |
| 230 | Insect Damaged | 142.860 | 153.650 | 1373.650 | 2 | 16 | 0.100 | Insect Damaged | 3.096 | -0.539 | 0.446 | 0 | 0.554 |
| 231 | Insect Damaged | 145.940 | 156.540 | 1430.780 | 2 | 16 | 0.100 | Insect Damaged | 3.498 | -0.478 | 0.470 | 0 | 0.530 |
| 232 | Insect Damaged | 123.840 | 134.460 | 1049.490 | 2 | 16 | 0.110 | Insect Damaged | 1.278 | -0.357 | 0.375 | 0 | 0.625 |
| 233 | Insect Damaged | 144.640 | 155.430 | 1418.030 | 2 | 16 | 0.100 | Insect Damaged | 3.346 | -0.463 | 0.464 | 0 | 0.536 |
| 234 | Insect Damaged | 155.740 | 166.130 | 1622.290 | 3 | 16 | 0.090 | Good | 3.730 | 0.828 | 0.592 | 0 | 0.408 |
| 235 | Insect Damaged | 131.630 | 142.780 | 1175.260 | 2 | 16 | 0.110 | Insect Damaged | 2.419 | -0.367 | 0.428 | 0 | 0.572 |
| 236 | Insect Damaged | 120.710 | 131.320 | 997.600 | 2 | 16 | 0.120 | Insect Damaged | 1.674 | -0.054 | 0.419 | 0 | 0.581 |
| 237 | Insect Damaged | 135.750 | 146.850 | 1256.940 | 2 | 16 | 0.110 | Insect Damaged | 2.985 | -0.280 | 0.462 | 0 | 0.538 |
| 238 | Insect Damaged | 121.740 | 132.760 | 1014.500 | 2 | 16 | 0.120 | Insect Damaged | 1.869 | -0.082 | 0.426 | 0 | 0.574 |
| 239 | Insect Damaged | 142.900 | 153.120 | 1374.030 | 2 | 16 | 0.100 | Insect Damaged | 3.027 | -0.494 | 0.446 | 0 | 0.554 |
| 240 | Insect Damaged | 136.680 | 147.340 | 1265.550 | 2 | 16 | 0.110 | Insect Damaged | 3.054 | -0.269 | 0.467 | 0 | 0.533 |
| 241 | Insect Damaged | 131.750 | 142.660 | 1176.330 | 2 | 16 | 0.110 | Insect Damaged | 2.404 | -0.351 | 0.428 | 0 | 0.572 |
| 242 | Insect Damaged | 143.990 | 154.320 | 1397.960 | 2 | 16 | 0.100 | Insect Damaged | 3.194 | -0.470 | 0.456 | 0 | 0.544 |
| 243 | Insect Damaged | 142.870 | 153.650 | 1373.750 | 2 | 16 | 0.100 | Insect Damaged | 3.096 | -0.538 | 0.446 | 0 | 0.554 |
| 244 | Insect Damaged | 131.110 | 142.670 | 1092.580 | 2 | 16 | 0.110 | Insect Damaged | 2.374 | -0.725 | 0.396 | 0 | 0.604 |
| 245 | Insect Damaged | 167.460 | 178.630 | 1881.000 | 3 | 16 | 0.080 | Good | 4.640 | 0.794 | 0.631 | 0 | 0.369 |
| 246 | Insect Damaged | 133.830 | 144.830 | 1216.630 | 2 | 16 | 0.110 | Insect Damaged | 2.705 | -0.321 | 0.445 | 0 | 0.555 |
| 247 | Insect Damaged | 135.890 | 146.730 | 1246.690 | 2 | 16 | 0.110 | Insect Damaged | 2.966 | -0.313 | 0.459 | 0 | 0.541 |
| 248 | Insect Damaged | 157.740 | 168.480 | 1660.420 | 3 | 16 | 0.090 | Good | 4.053 | 0.834 | 0.607 | 0 | 0.393 |
| 249 | Insect Damaged | 142.690 | 153.760 | 1372.010 | 2 | 16 | 0.100 | Insect Damaged | 3.109 | -0.557 | 0.445 | 0 | 0.555 |
| 250 | Insect Damaged | 142.770 | 153.640 | 1372.780 | 2 | 16 | 0.100 | Insect Damaged | 3.094 | -0.543 | 0.445 | 0 | 0.555 |
| 251 | Insect Damaged | 126.820 | 137.830 | 704.550 | 2 | 16 | 0.110 | Insect Damaged | 1.599 | -2.081 | 0.262 | 0 | 0.738 |
| 252 | Insect Damaged | 142.560 | 153.670 | 1370.760 | 2 | 16 | 0.100 | Insect Damaged | 3.097 | -0.557 | 0.444 | 0 | 0.556 |
| 253 | Insect Damaged | 155.800 | 166.970 | 1731.110 | 2 | 16 | 0.090 | Good | 4.150 | -0.192 | 0.526 | 0 | 0.474 |
| 254 | Insect Damaged | 143.540 | 154.320 | 1393.590 | 2 | 16 | 0.100 | Insect Damaged | 3.191 | -0.496 | 0.454 | 0 | 0.546 |
| 255 | Insect Damaged | 142.640 | 153.650 | 1371.530 | 2 | 16 | 0.100 | Insect Damaged | 3.095 | -0.551 | 0.445 | 0 | 0.555 |
| 256 | Insect Damaged | 155.320 | 166.540 | 1617.910 | 2 | 16 | 0.090 | Insect Damaged | 4.053 | -0.656 | 0.482 | 0 | 0.518 |
| 257 | Insect Damaged | 153.610 | 164.460 | 1583.600 | 2 | 16 | 0.090 | Insect Damaged | 3.768 | -0.662 | 0.468 | 0 | 0.532 |
| 258 | Insect Damaged | 137.670 | 148.670 | 1274.720 | 2 | 16 | 0.110 | Insect Damaged | 3.232 | -0.322 | 0.471 | 0 | 0.529 |
| 259 | Insect Damaged | 156.840 | 167.430 | 1650.940 | 2 | 16 | 0.090 | Insect Damaged | 4.184 | -0.562 | 0.496 | 0 | 0.504 |
| 260 | Insect Damaged | 142.960 | 153.610 | 1374.610 | 2 | 16 | 0.100 | Insect Damaged | 3.091 | -0.530 | 0.446 | 0 | 0.554 |
| 261 | Insect Damaged | 160.760 | 171.320 | 1728.600 | 3 | 16 | 0.090 | Good | 4.452 | 0.945 | 0.634 | 0 | 0.366 |
| 262 | Insect Damaged | 135.840 | 146.850 | 1246.230 | 3 | 16 | 0.110 | Good | 2.709 | 1.100 | 0.566 | 0 | 0.434 |
| 263 | Insect Damaged | 151.630 | 162.760 | 1547.240 | 3 | 16 | 0.090 | Good | 3.257 | 0.713 | 0.560 | 0 | 0.440 |
| 264 | Insect Damaged | 142.680 | 153.120 | 1371.920 | 2 | 16 | 0.100 | Insect Damaged | 3.026 | -0.506 | 0.445 | 0 | 0.555 |
| 265 | Insect Damaged | 156.990 | 167.340 | 1440.270 | 3 | 16 | 0.090 | Good | 3.825 | -0.040 | 0.523 | 0 | 0.477 |
| 266 | Insect Damaged | 135.850 | 146.660 | 125.850 | 2 | 16 | 0.110 | Insect Damaged | 2.559 | -5.169 | 0.130 | 0 | 0.870 |
| 267 | Insect Damaged | 143.850 | 154.320 | 1396.600 | 2 | 16 | 0.100 | Insect Damaged | 3.193 | -0.478 | 0.456 | 0 | 0.544 |
| 268 | Insect Damaged | 143.900 | 153.650 | 1383.170 | 2 | 16 | 0.100 | Insect Damaged | 3.102 | -0.482 | 0.451 | 0 | 0.549 |
| 269 | Insect Damaged | 155.840 | 166.540 | 1623.330 | 3 | 16 | 0.090 | Good | 3.784 | 0.801 | 0.592 | 0 | 0.408 |
| 270 | Insect Damaged | 153.940 | 164.460 | 1587.010 | 3 | 16 | 0.090 | Good | 3.497 | 0.783 | 0.577 | 0 | 0.423 |
| 271 | Insect Damaged | 144.830 | 155.430 | 1406.110 | 2 | 16 | 0.100 | Insect Damaged | 3.343 | -0.512 | 0.460 | 0 | 0.540 |
| 272 | Insect Damaged | 136.730 | 147.380 | 1266.010 | 2 | 16 | 0.110 | Insect Damaged | 3.059 | -0.269 | 0.467 | 0 | 0.533 |
| 273 | Insect Damaged | 136.990 | 147.640 | 1268.420 | 2 | 16 | 0.110 | Insect Damaged | 3.095 | -0.276 | 0.468 | 0 | 0.532 |
| 274 | Insect Damaged | 142.360 | 153.820 | 1368.840 | 2 | 16 | 0.100 | Insect Damaged | 3.115 | -0.581 | 0.443 | 0 | 0.557 |
| 275 | Insect Damaged | 136.820 | 147.430 | 1266.850 | 2 | 16 | 0.110 | Insect Damaged | 3.066 | -0.268 | 0.467 | 0 | 0.533 |
| 276 | Insect Damaged | 142.840 | 153.610 | 1373.460 | 2 | 16 | 0.100 | Insect Damaged | 3.091 | -0.537 | 0.446 | 0 | 0.554 |
| 277 | Insect Damaged | 150.840 | 161.320 | 1523.630 | 3 | 16 | 0.090 | Good | 3.060 | 0.715 | 0.551 | 0 | 0.449 |
| 278 | Insect Damaged | 148.790 | 159.540 | 1473.160 | 2 | 16 | 0.100 | Insect Damaged | 3.908 | -0.494 | 0.489 | 0 | 0.511 |
| 279 | Insect Damaged | 135.110 | 146.430 | 1239.540 | 2 | 16 | 0.110 | Insect Damaged | 2.923 | -0.331 | 0.455 | 0 | 0.545 |
| 280 | Insect Damaged | 135.320 | 146.750 | 1241.460 | 2 | 16 | 0.110 | Insect Damaged | 2.966 | -0.346 | 0.456 | 0 | 0.544 |
| 281 | Insect Damaged | 135.530 | 146.810 | 1243.390 | 2 | 16 | 0.110 | Insect Damaged | 2.975 | -0.339 | 0.457 | 0 | 0.543 |
| 282 | Insect Damaged | 152.900 | 163.720 | 1560.200 | 3 | 16 | 0.090 | Good | 3.389 | 0.711 | 0.566 | 0 | 0.434 |
| 283 | Insect Damaged | 135.650 | 146.760 | 1244.490 | 2 | 16 | 0.110 | Insect Damaged | 2.969 | -0.329 | 0.457 | 0 | 0.543 |
| 284 | Insect Damaged | 153.830 | 164.460 | 1585.870 | 3 | 16 | 0.090 | Good | 3.496 | 0.776 | 0.576 | 0 | 0.424 |
| 285 | Insect Damaged | 144.790 | 155.430 | 1405.720 | 2 | 16 | 0.130 | Good | 5.828 | 0.450 | 0.657 | 0 | 0.343 |
| 286 | Insect Damaged | 155.670 | 167.130 | 1621.560 | 3 | 16 | 0.090 | Good | 3.859 | 0.743 | 0.591 | 0 | 0.409 |
| 287 | Insect Damaged | 131.750 | 142.780 | 1176.330 | 2 | 16 | 0.110 | Insect Damaged | 2.420 | -0.361 | 0.428 | 0 | 0.572 |
| 288 | Insect Damaged | 141.900 | 152.640 | 1351.420 | 2 | 16 | 0.100 | Insect Damaged | 2.955 | -0.568 | 0.437 | 0 | 0.563 |
| 289 | Insect Damaged | 137.540 | 148.670 | 1273.510 | 2 | 16 | 0.110 | Insect Damaged | 3.231 | -0.329 | 0.470 | 0 | 0.530 |
| 290 | Insect Damaged | 156.620 | 167.430 | 1648.630 | 3 | 16 | 0.090 | Good | 3.910 | 0.850 | 0.602 | 0 | 0.398 |
| 291 | Insect Damaged | 142.800 | 153.610 | 1373.070 | 2 | 16 | 0.100 | Insect Damaged | 3.090 | -0.539 | 0.445 | 0 | 0.555 |
| 292 | Insect Damaged | 160.790 | 171.320 | 1728.920 | 3 | 16 | 0.090 | Good | 4.452 | 0.947 | 0.635 | 0 | 0.365 |
| 293 | Insect Damaged | 158.840 | 169.540 | 1689.780 | 3 | 16 | 0.090 | Good | 4.203 | 0.892 | 0.619 | 0 | 0.381 |
| 294 | Insect Damaged | 135.850 | 146.430 | 1246.330 | 2 | 16 | 0.110 | Insect Damaged | 2.927 | -0.291 | 0.459 | 0 | 0.541 |
| 295 | Insect Damaged | 135.850 | 146.750 | 1246.330 | 2 | 16 | 0.110 | Insect Damaged | 2.969 | -0.317 | 0.458 | 0 | 0.542 |
| 296 | Insect Damaged | 135.690 | 146.810 | 1244.860 | 2 | 16 | 0.110 | Insect Damaged | 2.976 | -0.330 | 0.458 | 0 | 0.542 |
| 297 | Insect Damaged | 152.780 | 163.720 | 1558.970 | 3 | 16 | 0.090 | Good | 3.388 | 0.704 | 0.565 | 0 | 0.435 |
| 298 | Insect Damaged | 145.330 | 156.760 | 1424.800 | 2 | 16 | 0.100 | Insect Damaged | 3.523 | -0.531 | 0.467 | 0 | 0.533 |
| 299 | Insect Damaged | 137.740 | 148.630 | 1275.370 | 2 | 16 | 0.110 | Insect Damaged | 3.227 | -0.314 | 0.471 | 0 | 0.529 |
| 300 | Insect Damaged | 160.730 | 171.460 | 1728.270 | 3 | 16 | 0.090 | Good | 4.470 | 0.932 | 0.634 | 0 | 0.366 |

A = Amplitude, F = Frequency, I = Intensity, T = Period, V = Velocity, ʎ = Wavelength,

‘Pred. Yam quality’ is the predicted yam quality class.

Quality class: 1= Good, 2 = Diseased damaged and 3 = Insect damaged

‘DSF1’ is ‘Discriminant Scores from Function (Model) 1’.

‘DSF2’ is ‘Discriminant Scores from Function (Model) 2’,

‘PMC1’ is the ‘Probabilities of Membership in Yam Quality Class 1’.

‘PMC2’ is the ‘Probabilities of Membership in Yam Quality Class 2’.

‘PMC3’ is the ‘Probabilities of Membership in Yam Quality Class 3’

Table S5: Test for equality of variance, group means and covariance matrices of white yam quality for two acoustic techniques

| Tests of Equality of Group Means | | | | | | | | | | | | | |
| --- | --- | --- | --- | --- | --- | --- | --- | --- | --- | --- | --- | --- | --- |
| Acoustic Property | Software Sound generation technique | | | | | | Surface impact sound generation technique | | | | | | |
|  | Wilks' Lambda | F | df1 | df2 | Sig. | | Wilks' Lambda | F | df1 | df2 | | Sig. | |
| Amplitude | 0.363 | 260.709 | 2 | 297 | 4.24E-66 | | 0.211 | 553.905 | 2 | 297 | | 6.07E-101 | |
| Frequency | ^a^ |  |  |  |  | | 0.083 | 1634.940 | 2 | 297 | | 4.89E-161 | |
| Intensity | 0.995 | 0.803 | 2 | 297 | 4.49E-01 | | 0.456 | 176.994 | 2 | 297 | | 2.45E-51 | |
| Period | ^a^ |  |  |  |  | | 0.285 | 372.534 | 2 | 297 | | 1.11E-81 | |
| Velocity | ^a^ |  |  |  |  | | ^a^ |  |  |  | |  | |
| Wavelength | ^a^ |  |  |  |  | | 0.927 | 11.763 | 2 | 297 | | 1.21E-05 | |
| *a. Cannot be computed because this variable is a constant.* | | | | | | | | | | | | | |
| Box's Test of Equality of Covariance Matrices | | | | | | | | | | | | | |
|  | Log Determinants | |  |  |  | Log Determinants | | | | | | | |
| Yam quality | Rank | Log Determinant |  |  |  | Rank | | Log Determinant |  | |  | |  |
| Good | 2 | 25.283 |  |  |  | 5 | | 9.486 |  | |  | |  |
| Disease damaged | 2 | 3.781 |  |  |  | 5 | | 1.574 |  | |  | |  |
| Insect Damaged | 2 | 12.435 |  |  |  | 5 | | 1.344 |  | |  | |  |
| Pooled within-groups | 2 | 23.787 |  |  |  | 5 | | 8.326 |  | |  | |  |
| *The ranks and natural logarithms of determinants printed are those of the group covariance matrices.* | | | | | | | | | | | | | |
| Box's Test Results | | | | | | | | | | | | | |
| Box's M | | 2956.271 |  |  |  |  | | 1244.839 |  | |  | |  |
| F | Approx. | 487.918 |  |  |  |  | | 40.497 |  | |  | |  |
|  | df1 | 6.000 |  |  |  |  | | 30 |  | |  | |  |
|  | df2 | 2198439.692 |  |  |  |  | | 279508.225 |  | |  | |  |
|  | Sig. | 0.000 |  |  |  |  | | 5.92E-236 |  | |  | |  |
| *Tests null hypothesis of equal population covariance matrices.* | | | | | | | | | | | | | |

Table S6: Summary of canonical discriminant score functions of white yam for both acoustic techniques

| Software Sound generation technique | | | | | Surface impact sound generation technique | | | |
| --- | --- | --- | --- | --- | --- | --- | --- | --- |
| Eigen values | | | | | | | | |
| Function (DSF) | Eigen value | % of Variance | Cumulative % | Canonical Correlation | Eigen value | % of Variance | Cumulative % | Canonical Correlation |
| 1 | 1.766^a^ | 99.870 | 99.870 | 0.799 | 21.669^a^ | 99.910 | 99.910 | 0.978 |
| 2 | 0.002^a^ | 0.130 | 100.000 | 0.048 | 0.020^a^ | 0.090 | 100.000 | 0.139 |
| *a. First 2 canonical discriminant functions were used in the analysis.* | | | | | | | | |
| Wilks' Lambda | | | | | | | | |
| Test of Function(s) (DSF) | Wilks' Lambda | Chi-square | df | Sig. | Wilks' Lambda | Chi-square | df | Sig. |
| 1 through 2 | 0.361 | 302.369 | 4 | 3.34E-64 | 0.043 | 926.420 | 10 | 1.310E-192 |
| 2 | 0.998 | 0.680 | 1 | 0.410 | 0.981 | 5.722 | 4 | 0.221 |
| Standardized Canonical Discriminant Function Coefficients | | | | | | | | |
| Acoustic Property | Function (DSF) | |  |  | Function (DSF) | |  |  |
|  | 1 | 2 |  |  | 1 | 2 |  |  |
| Amplitude | 1.000 | 0.042 |  |  | 0.042 | 0.266 |  |  |
| Frequency |  |  |  |  | 1.318 | -0.822 |  |  |
| Intensity | -0.078 | 0.998 |  |  | 0.094 | 1.152 |  |  |
| Period |  |  |  |  | -0.104 | 0.542 |  |  |
| Wavelength |  |  |  |  | 0.948 | 0.368 |  |  |
| Structure Matrix | | | | | | | | |
| Acoustic Property | Function (DSF) | |  |  | Function (DSF) | |  |  |
|  | 1 | 2 |  |  | 1 | 2 |  |  |
| Frequency |  |  |  |  | 0.713^*^ | 0.154 |  |  |
| Intensity | -0.042 | 0.999^*^ |  |  | 0.233 | 0.746^*^ |  |  |
| Amplitude | 0.997^*^ | 0.078 |  |  | 0.415 | 0.509^*^ |  |  |
| Period |  |  |  |  | 0.340 | 0.423^*^ |  |  |
| Wavelength |  |  |  |  | 0.060 | -0.265^*^ |  |  |
| *Pooled within-groups correlations between discriminating variables and standardized canonical discriminant functions   Variables ordered by absolute size of correlation within function.* | | | | | | | | |
| **. Largest absolute correlation between each variable and any discriminant function* | | | | | | | | |
| Discriminant Score Function (DSF) Equations | | | | | | | | |
| DSF1 = 0.086 A - 6.149 I - 24.855 | | | | | DSF1 = 0.002 A + 0.130 F + 3.55E-4 I - 0.273 T + 82.855 W - 25.374 | | | |
| DSF2 = 0.004 A + 7.888 E-5 I - 1.284 | | | | | DSF2 = 0.015 A - 0.081 F + 0.004 I + 1.425 T + 32.153 W - 2.287 | | | |
| *A = Amplitude, F = Frequency, I = Intensity, T = Period, W = Wavelength* | | | | | |  |  |  |

Table S7: Acoustic properties of yellow yam qualities for sound generation technique and their classification training result using machine learning algorithm

| S/N | Yam quality | A | Freq | V | λ | T | I | Pred. Yam quality | DSF1 | DSF2 | PMC1 | PMC2 | PMC3 |
| --- | --- | --- | --- | --- | --- | --- | --- | --- | --- | --- | --- | --- | --- |
| 1 | Good | 245.76 | 143 | 128484.86 | 898.5 | 0.01 | 6039 | Good | -1.90143 | -0.062 | 0.8962 | 0.0956 | 0.0082 |
| 2 | Good | 272.4 | 160 | 160849.54 | 1005.3 | 0.01 | 763 | Good | -1.304 | 1.0466 | 0.8713 | 0.0839 | 0.0448 |
| 3 | Good | 264.41 | 159 | 158845.21 | 999.03 | 0.01 | 6991 | Good | -1.338 | 0.979 | 0.8742 | 0.0851 | 0.0407 |
| 4 | Good | 262.61 | 142 | 126694.15 | 892.21 | 0.01 | 6896 | Good | -1.93758 | -0.126 | 0.8966 | 0.096 | 0.0074 |
| 5 | Good | 278.51 | 140 | 123150.43 | 879.65 | 0.01 | 7756 | Good | -2.01024 | -0.255 | 0.8973 | 0.0967 | 0.006 |
| 6 | Good | 277.16 | 146 | 133932.38 | 917.35 | 0.01 | 7681 | Good | -1.79354 | 0.1305 | 0.8944 | 0.0944 | 0.0112 |
| 7 | Good | 220.96 | 157 | 154874.23 | 986.46 | 0.01 | 4882 | Good | -1.4069 | 0.848 | 0.8796 | 0.0868 | 0.0336 |
| 8 | Good | 262.43 | 161 | 162866.45 | 1011.6 | 0.01 | 6886 | Good | -1.26962 | 1.1112 | 0.8677 | 0.083 | 0.0493 |
| 9 | Good | 277.81 | 160 | 160849.54 | 1005.3 | 0.01 | 7717 | Good | -1.30373 | 1.0448 | 0.8711 | 0.0841 | 0.0448 |
| 10 | Good | 2272.4 | 153 | 147083.08 | 961.33 | 0.01 | 74201 | Good | -1.54444 | 0.5583 | 0.885 | 0.0924 | 0.0226 |
| 11 | Good | 245.76 | 142 | 126694.15 | 892.21 | 0.01 | 6039 | Good | -1.93761 | -0.126 | 0.8966 | 0.096 | 0.0074 |
| 12 | Good | 275.86 | 147 | 135773.35 | 923.63 | 0.01 | 7609 | Good | -1.75782 | 0.1951 | 0.8937 | 0.0939 | 0.0124 |
| 13 | Good | 273.31 | 155 | 150953.53 | 973.89 | 0.01 | 7469 | Good | -1.47613 | 0.7158 | 0.8838 | 0.0886 | 0.0276 |
| 14 | Good | 264.32 | 149 | 139493 | 936.19 | 0.01 | 6986 | Good | -1.68674 | 0.3248 | 0.8921 | 0.0928 | 0.0152 |
| 15 | Good | 262.61 | 138 | 119656.98 | 867.08 | 0.01 | 6896 | Good | -2.08341 | -0.382 | 0.8979 | 0.0972 | 0.0049 |
| 16 | Good | 275.66 | 140 | 123150.43 | 980.18 | 0.01 | 6034 | Good | -2.01015 | -0.254 | 0.8973 | 0.0967 | 0.0061 |
| 17 | Good | 245.66 | 156 | 152907.6 | 980.18 | 0.01 | 6034 | Good | -1.44146 | 0.7819 | 0.8818 | 0.0877 | 0.0305 |
| 18 | Good | 220.75 | 149 | 139493 | 936.19 | 0.01 | 4873 | Good | -1.6868 | 0.3256 | 0.8921 | 0.0927 | 0.0152 |
| 19 | Good | 280.56 | 139 | 121397.42 | 873.36 | 0.01 | 78713 | Good | -2.04395 | -0.336 | 0.8957 | 0.0988 | 0.0055 |
| 20 | Good | 262169 | 162 | 164895.92 | 1017.9 | 0.01 | 69006 | Good | -1.39128 | -0.159 | 0.7705 | 0.2036 | 0.0259 |
| 21 | Good | 307.22 | 156 | 152907.6 | 980.18 | 0.01 | 9438 | Good | -1.44137 | 0.7808 | 0.8817 | 0.0878 | 0.0305 |
| 22 | Good | 299.81 | 149 | 139493 | 936.19 | 0.01 | 89886 | Good | -1.68348 | 0.3041 | 0.8899 | 0.0949 | 0.0152 |
| 23 | Good | 301.96 | 157 | 154874.23 | 986.46 | 0.01 | 91179 | Good | -1.40353 | 0.8262 | 0.8774 | 0.0889 | 0.0337 |
| 24 | Good | 304.95 | 144 | 130288.13 | 904.78 | 0.01 | 92994 | Good | -1.86196 | -0.02 | 0.8934 | 0.0975 | 0.0092 |
| 25 | Good | 293.91 | 158 | 156853.44 | 992.74 | 0.01 | 8638 | Good | -1.37232 | 0.9125 | 0.877 | 0.086 | 0.037 |
| 26 | Good | 299.67 | 155 | 150953.53 | 973.89 | 0.01 | 89802 | Good | -1.47288 | 0.6953 | 0.8817 | 0.0906 | 0.0277 |
| 27 | Good | 276.49 | 143 | 128484.87 | 898.5 | 0.01 | 76446 | Good | -1.89866 | -0.08 | 0.8943 | 0.0975 | 0.0083 |
| 28 | Good | 271.59 | 159 | 158845.21 | 999.03 | 0.01 | 7376 | Good | -1.33799 | 0.9789 | 0.8742 | 0.0851 | 0.0407 |
| 29 | Good | 288.67 | 160 | 160845.54 | 1E+06 | 0.01 | 83330 | Disease damaged | 0.28962 | 0.9771 | 0.1864 | 0.2442 | 0.5694 |
| 30 | Good | 275.49 | 140 | 123150.43 | 879.65 | 0.01 | 75895 | Good | -2.00754 | -0.272 | 0.8955 | 0.0985 | 0.0061 |
| 31 | Good | 278.12 | 162 | 160849.54 | 1005.3 | 0.01 | 77351 | Good | -1.19643 | 1.1239 | 0.8511 | 0.0904 | 0.0585 |
| 32 | Good | 282.2 | 139 | 121397.42 | 873.36 | 0.01 | 79637 | Good | -2.04391 | -0.336 | 0.8957 | 0.0989 | 0.0055 |
| 33 | Good | 297.56 | 143 | 128484.86 | 898.5 | 0.01 | 77040 | Good | -1.89865 | -0.08 | 0.8943 | 0.0975 | 0.0083 |
| 34 | Good | 285.9 | 150 | 141371.67 | 942.48 | 0.01 | 80599 | Good | -1.64845 | 0.3713 | 0.8891 | 0.094 | 0.0169 |
| 35 | Good | 290.01 | 146 | 133932.38 | 917.35 | 0.01 | 84106 | Good | -1.79052 | 0.1115 | 0.8924 | 0.0963 | 0.0112 |
| 36 | Good | 291.2 | 161 | 162866.45 | 1011.6 | 0.01 | 8426 | Good | -1.26957 | 1.1107 | 0.8677 | 0.0831 | 0.0493 |
| 37 | Good | 293.67 | 159 | 158845.21 | 999.03 | 0.01 | 86242 | Good | -1.33488 | 0.9592 | 0.8723 | 0.0869 | 0.0408 |
| 38 | Good | 300.12 | 139 | 121397.42 | 873.36 | 0.01 | 90072 | Good | -2.04351 | -0.339 | 0.8954 | 0.0991 | 0.0055 |
| 39 | Good | 260.15 | 158 | 156853.44 | 992.74 | 0.01 | 67678 | Good | -1.36996 | 0.898 | 0.8755 | 0.0874 | 0.0371 |
| 40 | Good | 230.13 | 149 | 134993 | 936.19 | 0.01 | 52960 | Good | -1.64441 | 0.2735 | 0.8806 | 0.1029 | 0.0166 |
| 41 | Good | 234.14 | 150 | 141371.67 | 942.48 | 0.01 | 54822 | Good | -1.64944 | 0.378 | 0.8898 | 0.0933 | 0.0168 |
| 42 | Good | 261.49 | 142 | 126694.15 | 892.21 | 0.01 | 0 | Good | -1.93785 | -0.125 | 0.8967 | 0.0958 | 0.0074 |
| 43 | Good | 256.17 | 147 | 135773.35 | 923.63 | 0.01 | 65623 | Good | -1.75551 | 0.1808 | 0.8922 | 0.0953 | 0.0124 |
| 44 | Good | 217.16 | 143 | 128484.86 | 898.5 | 0.01 | 4715.5 | Good | -1.90147 | -0.062 | 0.8962 | 0.0956 | 0.0083 |
| 45 | Good | 268.45 | 149 | 139493 | 936.19 | 0.01 | 72044 | Good | -1.68416 | 0.3086 | 0.8904 | 0.0944 | 0.0152 |
| 46 | Good | 264.43 | 153 | 147083.08 | 961.33 | 0.01 | 69923 | Good | -1.54339 | 0.5695 | 0.8856 | 0.0917 | 0.0227 |
| 47 | Good | 287.54 | 157 | 154874.23 | 986.46 | 0.01 | 82679 | Good | -1.40386 | 0.8284 | 0.8776 | 0.0887 | 0.0337 |
| 48 | Good | 266.43 | 150 | 141371.67 | 942.48 | 0.01 | 70985 | Good | -1.64882 | 0.3738 | 0.8894 | 0.0938 | 0.0169 |
| 49 | Good | 276.68 | 156 | 152907.6 | 980.18 | 0.01 | 75999 | Good | -1.43871 | 0.7644 | 0.88 | 0.0894 | 0.0305 |
| 50 | Good | 296.82 | 151 | 143262.91 | 948.76 | 0.01 | 88102 | Good | -1.61289 | 0.4345 | 0.8878 | 0.0936 | 0.0186 |
| 51 | Good | 276.12 | 143 | 132103.97 | 911.06 | 0.01 | 76242 | Good | -1.93121 | -0.047 | 0.9018 | 0.0905 | 0.0077 |
| 52 | Good | 288.11 | 143 | 128484.86 | 898.5 | 0.01 | 85007 | Good | -1.89833 | -0.082 | 0.8941 | 0.0977 | 0.0083 |
| 53 | Good | 275.49 | 155 | 150953.53 | 973.89 | 0.01 | 75895 | Good | -1.47342 | 0.6988 | 0.882 | 0.0903 | 0.0277 |
| 54 | Good | 272.9 | 160 | 160849.54 | 1005.3 | 0.01 | 74474 | Good | -1.30108 | 1.0283 | 0.8695 | 0.0856 | 0.0449 |
| 55 | Good | 279.11 | 161 | 162866.45 | 1011.6 | 0.01 | 77903 | Good | -1.26681 | 1.0935 | 0.866 | 0.0846 | 0.0494 |
| 56 | Good | 263.15 | 153 | 147083.08 | 961.33 | 0.01 | 69248 | Good | -1.54342 | 0.5697 | 0.8856 | 0.0917 | 0.0227 |
| 57 | Good | 252.9 | 159 | 158845.21 | 999.03 | 0.01 | 63958 | Good | -1.33574 | 0.9649 | 0.8728 | 0.0864 | 0.0408 |
| 58 | Good | 270.11 | 149 | 139493 | 936.19 | 0.01 | 72959 | Good | -1.68413 | 0.3084 | 0.8903 | 0.0944 | 0.0152 |
| 59 | Good | 246.76 | 151 | 143262.91 | 948.76 | 0.01 | 60891 | Good | -1.61394 | 0.4415 | 0.8885 | 0.0929 | 0.0186 |
| 60 | Good | 273.82 | 149 | 139493 | 936.19 | 0.01 | 74977 | Good | -1.68405 | 0.3079 | 0.8903 | 0.0945 | 0.0152 |
| 61 | Good | 271.61 | 139 | 121397.42 | 873.36 | 0.01 | 73772 | Good | -2.04414 | -0.335 | 0.8959 | 0.0987 | 0.0055 |
| 62 | Good | 243.11 | 138 | 119656.98 | 867.08 | 0.01 | 59103 | Good | -2.08133 | -0.395 | 0.8966 | 0.0985 | 0.0049 |
| 63 | Good | 259.17 | 162 | 164895.92 | 1017.9 | 0.01 | 67169 | Good | -1.2332 | 1.1625 | 0.8625 | 0.0833 | 0.0543 |
| 64 | Good | 276.54 | 160 | 160849.54 | 1005.3 | 0.01 | 76474 | Good | -1.30101 | 1.0277 | 0.8694 | 0.0857 | 0.0449 |
| 65 | Good | 277.01 | 159 | 138845.21 | 999.03 | 0.01 | 76735 | Good | -1.15532 | 0.7834 | 0.8137 | 0.1277 | 0.0587 |
| 66 | Good | 296.11 | 153 | 147083.08 | 961.33 | 0.01 | 87681 | Good | -1.54271 | 0.565 | 0.8851 | 0.0921 | 0.0227 |
| 67 | Good | 240.54 | 146 | 133932.38 | 917.35 | 0.01 | 57859 | Good | -1.79153 | 0.1182 | 0.8931 | 0.0956 | 0.0112 |
| 68 | Good | 277.16 | 145 | 132103.97 | 911.06 | 0.01 | 76818 | Good | -1.82664 | 0.0489 | 0.8932 | 0.0966 | 0.0102 |
| 69 | Good | 271.81 | 158 | 156853.44 | 992.74 | 0.01 | 73881 | Good | -1.36972 | 0.8965 | 0.8753 | 0.0876 | 0.0371 |
| 70 | Good | 272.83 | 160 | 160849.54 | 1005.3 | 0.01 | 74436 | Good | -1.30109 | 1.0283 | 0.8695 | 0.0856 | 0.0449 |
| 71 | Good | 275.49 | 153 | 147083.08 | 961.33 | 0.01 | 75895 | Good | -1.54316 | 0.568 | 0.8854 | 0.0918 | 0.0227 |
| 72 | Good | 255.76 | 142 | 126694.15 | 892.21 | 0.01 | 60398 | Good | -1.93546 | -0.14 | 0.8952 | 0.0974 | 0.0075 |
| 73 | Good | 271.91 | 143 | 12848.86 | 898.5 | 0.01 | 73935 | Insect Damaged | -0.85844 | -1.11 | 0.3785 | 0.5789 | 0.0427 |
| 74 | Good | 276.4 | 152 | 145166.71 | 955.04 | 0.01 | 76397 | Good | -1.57819 | 0.5026 | 0.8869 | 0.0926 | 0.0206 |
| 75 | Good | 253.11 | 147 | 135773.35 | 923.63 | 0.01 | 64065 | Good | -1.75557 | 0.1812 | 0.8923 | 0.0953 | 0.0124 |
| 76 | Good | 236.19 | 157 | 154874.23 | 986.46 | 0.01 | 55786 | Good | -1.4049 | 0.8353 | 0.8783 | 0.0881 | 0.0336 |
| 77 | Good | 274.6 | 152 | 145166.71 | 955.04 | 0.01 | 75405 | Good | -1.57823 | 0.5029 | 0.8869 | 0.0925 | 0.0206 |
| 78 | Good | 249.59 | 153 | 147083.08 | 961.33 | 0.01 | 62295 | Good | -1.54369 | 0.5715 | 0.8858 | 0.0915 | 0.0227 |
| 79 | Good | 268.7 | 144 | 130288.13 | 904.78 | 0.01 | 72200 | Good | -1.86276 | -0.014 | 0.8939 | 0.0969 | 0.0092 |
| 80 | Good | 267.33 | 156 | 152907.6 | 980.18 | 0.01 | 0 | Good | -1.44172 | 0.7833 | 0.8819 | 0.0876 | 0.0305 |
| 81 | Good | 290.16 | 159 | 158845.21 | 999.03 | 0.01 | 84193 | Good | -1.33496 | 0.9597 | 0.8723 | 0.0869 | 0.0408 |
| 82 | Good | 243.6 | 140 | 123150.43 | 879.65 | 0.01 | 59341 | Good | -2.00818 | -0.267 | 0.8959 | 0.098 | 0.0061 |
| 83 | Good | 247.47 | 146 | 133932.38 | 917.34 | 0.01 | 61241 | Good | -1.7914 | 0.1174 | 0.893 | 0.0957 | 0.0112 |
| 84 | Good | 279.67 | 145 | 132103.97 | 911.06 | 0.01 | 78215 | Good | -1.82659 | 0.0485 | 0.8932 | 0.0967 | 0.0102 |
| 85 | Good | 261.72 | 120 | 90477.87 | 753.98 | 0.01 | 68497 | Good | -2.75956 | -1.524 | 0.9009 | 0.0984 | 0.0007 |
| 86 | Good | 273 | 149 | 139493 | 936.19 | 0.01 | 74529 | Good | -1.68407 | 0.308 | 0.8903 | 0.0945 | 0.0152 |
| 87 | Good | 259.69 | 160 | 160841.54 | 1005.3 | 0.01 | 67439 | Good | -1.30128 | 1.03 | 0.8696 | 0.0855 | 0.0449 |
| 88 | Good | 247.33 | 159 | 158845.21 | 999.03 | 0.01 | 61172 | Good | -1.33585 | 0.9657 | 0.8729 | 0.0863 | 0.0408 |
| 89 | Good | 239.43 | 149 | 139493 | 936.19 | 0.01 | 57327 | Good | -1.68473 | 0.3124 | 0.8908 | 0.094 | 0.0152 |
| 90 | Good | 270.46 | 159 | 999.03 | 958845 | 0.01 | 73149 | Disease damaged | 1.60185 | -0.491 | 0.0088 | 0.3788 | 0.6125 |
| 91 | Good | 270.56 | 148 | 929.91 | 137627 | 0.01 | 75203 | Insect Damaged | -0.27322 | -0.982 | 0.2014 | 0.6943 | 0.1043 |
| 92 | Good | 276.94 | 156 | 980.18 | 152908 | 0.01 | 76696 | Insect Damaged | 0.16877 | -0.597 | 0.1323 | 0.6401 | 0.2276 |
| 93 | Good | 271.59 | 162 | 1017.88 | 2E+06 | 0.01 | 73761 | Disease damaged | 2.81352 | -0.379 | 0.0005 | 0.1435 | 0.856 |
| 94 | Good | 266.09 | 149 | 936.19 | 139493 | 0.01 | 70804 | Insect Damaged | -0.21822 | -0.933 | 0.1926 | 0.6916 | 0.1159 |
| 95 | Good | 260.11 | 148 | 929.91 | 137627 | 0.01 | 0 | Insect Damaged | -0.2762 | -0.963 | 0.2047 | 0.6898 | 0.1056 |
| 96 | Good | 249.71 | 159 | 999.03 | 158845 | 0.01 | 62355 | Insect Damaged | 0.33428 | -0.449 | 0.109 | 0.5967 | 0.2943 |
| 97 | Good | 239.15 | 160 | 1005.31 | 160850 | 0.01 | 57193 | Insect Damaged | 0.38947 | -0.4 | 0.1016 | 0.5796 | 0.3188 |
| 98 | Good | 271 | 152 | 955.04 | 145167 | 0.01 | 73441 | Insect Damaged | -0.05248 | -0.789 | 0.166 | 0.6774 | 0.1566 |
| 99 | Good | 275.9 | 143 | 898.5 | 128485 | 0.01 | 76121 | Insect Damaged | -0.54876 | -1.223 | 0.2476 | 0.6917 | 0.0607 |
| 100 | Good | 280.6 | 155 | 973.89 | 150954 | 0.01 | 78736 | Insect Damaged | 0.11354 | -0.646 | 0.1404 | 0.6517 | 0.2079 |
| 101 | Insect Damaged | 293.33 | 148 | 929.91 | 137627 | 0.01 | 86042 | Insect Damaged | -0.27281 | -0.985 | 0.2009 | 0.695 | 0.1041 |
| 102 | Insect Damaged | 285.75 | 163 | 1024.16 | 166938 | 0.01 | 81653 | Insect Damaged | 0.55671 | -0.261 | 0.0802 | 0.5239 | 0.3958 |
| 103 | Insect Damaged | 250.11 | 165 | 1036.73 | 171060 | 0.01 | 62555 | Insect Damaged | 0.66694 | -0.16 | 0.0679 | 0.48 | 0.4522 |
| 104 | Insect Damaged | 285.17 | 140 | 879.65 | 123150 | 0.01 | 81322 | Insect Damaged | -0.71366 | -1.369 | 0.276 | 0.6808 | 0.0432 |
| 105 | Insect Damaged | 296.45 | 169 | 1061.86 | 179454 | 0.01 | 87883 | Disease damaged | 0.89008 | 0.0257 | 0.0462 | 0.3906 | 0.5632 |
| 106 | Insect Damaged | 306.91 | 155 | 973.89 | 150954 | 0.01 | 94194 | Insect Damaged | 0.11414 | -0.65 | 0.14 | 0.6526 | 0.2074 |
| 107 | Insect Damaged | 296.82 | 168 | 1055.58 | 177337 | 0.01 | 88102 | Disease damaged | 0.83451 | -0.022 | 0.0511 | 0.4137 | 0.5353 |
| 108 | Insect Damaged | 281.92 | 141 | 885.92 | 124916 | 0.01 | 79479 | Insect Damaged | -0.65871 | -1.32 | 0.2665 | 0.6851 | 0.0484 |
| 109 | Insect Damaged | 278.41 | 148 | 929.91 | 137627 | 0.01 | 77512 | Insect Damaged | -0.27314 | -0.982 | 0.2013 | 0.6944 | 0.1043 |
| 110 | Insect Damaged | 311.41 | 144 | 904.78 | 130288 | 0.01 | 96976 | Insect Damaged | -0.49288 | -1.18 | 0.2371 | 0.6953 | 0.0676 |
| 111 | Insect Damaged | 294.6 | 158 | 992.74 | 156853 | 0.01 | 86789 | Insect Damaged | 0.27985 | -0.503 | 0.1161 | 0.6138 | 0.2701 |
| 112 | Insect Damaged | 291.61 | 156 | 980.18 | 152908 | 0.01 | 85036 | Insect Damaged | 0.16909 | -0.599 | 0.132 | 0.6407 | 0.2273 |
| 113 | Insect Damaged | 284.99 | 150 | 942.48 | 141372 | 0.01 | 81219 | Insect Damaged | -0.16263 | -0.887 | 0.1832 | 0.6886 | 0.1281 |
| 114 | Insect Damaged | 299.75 | 166 | 1043.01 | 173139 | 0.01 | 89850 | Disease damaged | 0.7235 | -0.119 | 0.0617 | 0.4594 | 0.4788 |
| 115 | Insect Damaged | 298 | 168 | 1055.58 | 177337 | 0.01 | 88804 | Disease damaged | 0.83454 | -0.023 | 0.0511 | 0.4137 | 0.5353 |
| 116 | Insect Damaged | 301.17 | 149 | 936.19 | 139493 | 0.01 | 90703 | Insect Damaged | -0.21745 | -0.938 | 0.1917 | 0.6928 | 0.1155 |
| 117 | Insect Damaged | 273.75 | 151 | 948.76 | 143263 | 0.01 | 74939 | Insect Damaged | -0.10766 | -0.837 | 0.1747 | 0.6835 | 0.1419 |
| 118 | Insect Damaged | 317.69 | 153 | 961.33 | 14708 | 0.01 | 100927 | Insect Damaged | -0.20584 | -0.741 | 0.213 | 0.6504 | 0.1366 |
| 119 | Insect Damaged | 315.01 | 145 | 911.06 | 132104 | 0.01 | 99231 | Insect Damaged | -0.4377 | -1.132 | 0.2277 | 0.6969 | 0.0754 |
| 120 | Insect Damaged | 250.76 | 149 | 936.19 | 139493 | 0.01 | 62881 | Insect Damaged | -0.21853 | -0.931 | 0.1929 | 0.6911 | 0.116 |
| 121 | Insect Damaged | 264.43 | 168 | 1055.58 | 177337 | 0.01 | 69923 | Disease damaged | 0.83381 | -0.018 | 0.0512 | 0.4125 | 0.5363 |
| 122 | Insect Damaged | 285.71 | 170 | 1068.14 | 181584 | 0.01 | 81630 | Disease damaged | 0.94543 | 0.0754 | 0.0417 | 0.3673 | 0.591 |
| 123 | Insect Damaged | 297.9 | 150 | 942.48 | 141372 | 0.01 | 88744 | Insect Damaged | -0.16234 | -0.889 | 0.1829 | 0.6891 | 0.128 |
| 124 | Insect Damaged | 306.11 | 161 | 1011.59 | 162866 | 0.01 | 93703 | Insect Damaged | 0.44629 | -0.361 | 0.0937 | 0.564 | 0.3423 |
| 125 | Insect Damaged | 289.75 | 163 | 1024.16 | 166938 | 0.01 | 83955 | Insect Damaged | 0.5568 | -0.262 | 0.0802 | 0.5241 | 0.3957 |
| 126 | Insect Damaged | 249.76 | 149 | 936.19 | 139493 | 0.01 | 62380 | Insect Damaged | -0.21855 | -0.93 | 0.1929 | 0.6911 | 0.116 |
| 127 | Insect Damaged | 291.11 | 140 | 879.65 | 123150 | 0.01 | 84745 | Insect Damaged | -0.71352 | -1.369 | 0.2758 | 0.681 | 0.0432 |
| 128 | Insect Damaged | 275.76 | 155 | 973.89 | 150954 | 0.01 | 76044 | Insect Damaged | 0.11344 | -0.645 | 0.1405 | 0.6515 | 0.208 |
| 129 | Insect Damaged | 269.11 | 165 | 1036.72 | 171060 | 0.01 | 72420 | Insect Damaged | 0.66731 | -0.163 | 0.0678 | 0.4806 | 0.4516 |
| 130 | Insect Damaged | 276.16 | 170 | 1068.14 | 181584 | 0.01 | 76264 | Disease damaged | 0.94522 | 0.0768 | 0.0417 | 0.367 | 0.5913 |
| 131 | Insect Damaged | 281.11 | 145 | 911.06 | 132104 | 0.01 | 79023 | Insect Damaged | -0.43848 | -1.127 | 0.2287 | 0.6957 | 0.0756 |
| 132 | Insect Damaged | 289.6 | 149 | 936.19 | 139493 | 0.01 | 83868 | Insect Damaged | -0.21772 | -0.936 | 0.192 | 0.6924 | 0.1156 |
| 133 | Insect Damaged | 299.11 | 166 | 1043 | 173139 | 0.01 | 89467 | Disease damaged | 0.72348 | -0.119 | 0.0617 | 0.4594 | 0.4789 |
| 134 | Insect Damaged | 263.71 | 170 | 1068.14 | 181584 | 0.01 | 69543 | Disease damaged | 0.94496 | 0.0786 | 0.0418 | 0.3666 | 0.5916 |
| 135 | Insect Damaged | 246.32 | 159 | 999.03 | 158845 | 0.01 | 60674 | Insect Damaged | 0.33421 | -0.449 | 0.1091 | 0.5966 | 0.2943 |
| 136 | Insect Damaged | 302.11 | 150 | 942.48 | 141372 | 0.01 | 91270 | Insect Damaged | -0.16224 | -0.89 | 0.1828 | 0.6893 | 0.1279 |
| 137 | Insect Damaged | 298.41 | 153 | 942.48 | 141372 | 0.01 | 91270 | Insect Damaged | -0.00542 | -0.745 | 0.1589 | 0.6706 | 0.1705 |
| 138 | Insect Damaged | 290.56 | 157 | 986.46 | 154874 | 0.01 | 84425 | Insect Damaged | 0.2244 | -0.551 | 0.124 | 0.6278 | 0.2482 |
| 139 | Insect Damaged | 288.71 | 169 | 1061.86 | 179454 | 0.01 | 83353 | Disease damaged | 0.8899 | 0.0269 | 0.0462 | 0.3903 | 0.5634 |
| 140 | Insect Damaged | 275.69 | 170 | 1068.14 | 181584 | 0.01 | 76005 | Disease damaged | 0.94521 | 0.0769 | 0.0417 | 0.367 | 0.5913 |
| 141 | Insect Damaged | 261.79 | 167 | 1049.29 | 175232 | 0.01 | 68534 | Disease damaged | 0.77821 | -0.066 | 0.0564 | 0.4354 | 0.5082 |
| 142 | Insect Damaged | 305.11 | 147 | 923.63 | 135773 | 0.01 | 93092 | Insect Damaged | -0.32769 | -1.035 | 0.2097 | 0.6968 | 0.0936 |
| 143 | Insect Damaged | 305.11 | 147 | 923.63 | 135773 | 0.01 | 93092 | Insect Damaged | -0.32769 | -1.035 | 0.2097 | 0.6968 | 0.0936 |
| 144 | Insect Damaged | 310.19 | 151 | 948.76 | 143263 | 0.01 | 96218 | Insect Damaged | -0.10684 | -0.843 | 0.1739 | 0.6848 | 0.1414 |
| 145 | Insect Damaged | 248.3 | 150 | 942.48 | 141372 | 0.01 | 61653 | Insect Damaged | -0.16338 | -0.882 | 0.184 | 0.6874 | 0.1286 |
| 146 | Insect Damaged | 291.63 | 163 | 1024.16 | 166938 | 0.01 | 85048 | Insect Damaged | 0.55684 | -0.262 | 0.0802 | 0.5242 | 0.3957 |
| 147 | Insect Damaged | 283.43 | 169 | 1061.86 | 179454 | 0.01 | 80333 | Disease damaged | 0.88978 | 0.0276 | 0.0462 | 0.3902 | 0.5636 |
| 148 | Insect Damaged | 269.11 | 161 | 1011.59 | 162866 | 0.01 | 72420 | Insect Damaged | 0.44547 | -0.355 | 0.0941 | 0.5626 | 0.3433 |
| 149 | Insect Damaged | 293.7 | 150 | 942.48 | 141372 | 0.01 | 86260 | Insect Damaged | -0.16243 | -0.888 | 0.183 | 0.6889 | 0.128 |
| 150 | Insect Damaged | 279.6 | 150 | 942.48 | 141372 | 0.01 | 78176 | Insect Damaged | -0.16274 | -0.886 | 0.1833 | 0.6885 | 0.1282 |
| 151 | Insect Damaged | 280.19 | 159 | 999.03 | 158845 | 0.01 | 78506 | Insect Damaged | 0.3349 | -0.453 | 0.1087 | 0.5978 | 0.2936 |
| 152 | Insect Damaged | 279 | 149 | 936.19 | 139493 | 0.01 | 77841 | Insect Damaged | -0.21795 | -0.934 | 0.1923 | 0.692 | 0.1157 |
| 153 | Insect Damaged | 273.82 | 166 | 1043.01 | 173139 | 0.01 | 74977 | Disease damaged | 0.72292 | -0.115 | 0.0619 | 0.4585 | 0.4796 |
| 154 | Insect Damaged | 264.42 | 171 | 1074.42 | 183727 | 0.01 | 69918 | Disease damaged | 1.00058 | 0.1266 | 0.0375 | 0.344 | 0.6184 |
| 155 | Insect Damaged | 246.93 | 147 | 923.63 | 135773 | 0.01 | 60974 | Insect Damaged | -0.32893 | -1.026 | 0.2111 | 0.6948 | 0.0941 |
| 156 | Insect Damaged | 296.68 | 168 | 1055.58 | 177337 | 0.01 | 88019 | Disease damaged | 0.83451 | -0.022 | 0.0511 | 0.4136 | 0.5353 |
| 157 | Insect Damaged | 290.12 | 166 | 10433.01 | 173139 | 0.01 | 84170 | Disease damaged | 0.6388 | -0.034 | 0.0751 | 0.4499 | 0.475 |
| 158 | Insect Damaged | 274.04 | 163 | 1024.16 | 166938 | 0.01 | 75098 | Insect Damaged | 0.55645 | -0.26 | 0.0803 | 0.5235 | 0.3962 |
| 159 | Insect Damaged | 306.19 | 151 | 948.76 | 143263 | 0.01 | 93752 | Insect Damaged | -0.10693 | -0.842 | 0.1739 | 0.6846 | 0.1414 |
| 160 | Insect Damaged | 301.94 | 159 | 999.03 | 158845 | 0.01 | 9E+06 | Insect Damaged | 0.69301 | -2.698 | 0.0113 | 0.936 | 0.0527 |
| 161 | Insect Damaged | 299.71 | 155 | 973.89 | 150954 | 0.01 | 89826 | Insect Damaged | 0.11397 | -0.649 | 0.1401 | 0.6524 | 0.2076 |
| 162 | Insect Damaged | 286.32 | 144 | 904.78 | 130288 | 0.01 | 81979 | Insect Damaged | -0.49346 | -1.176 | 0.2379 | 0.6944 | 0.0677 |
| 163 | Insect Damaged | 268.23 | 169 | 1061.86 | 179454 | 0.01 | 71947 | Disease damaged | 0.88946 | 0.0298 | 0.0463 | 0.3897 | 0.5641 |
| 164 | Insect Damaged | 269.54 | 160 | 1005.31 | 160850 | 0.01 | 7E+06 | Insect Damaged | 0.67506 | -2.19 | 0.0181 | 0.8945 | 0.0875 |
| 165 | Insect Damaged | 261.92 | 171 | 1074.42 | 183727 | 0.01 | 68602 | Disease damaged | 1.00053 | 0.1269 | 0.0376 | 0.344 | 0.6185 |
| 166 | Insect Damaged | 247.89 | 140 | 879.65 | 123150 | 0.01 | 6E+06 | Insect Damaged | -0.47337 | -2.875 | 0.0609 | 0.9254 | 0.0137 |
| 167 | Insect Damaged | 30563 | 151 | 948.76 | 143263 | 0.01 | 93410 | Insect Damaged | -0.12521 | -0.995 | 0.1619 | 0.7157 | 0.1224 |
| 168 | Insect Damaged | 304.91 | 150 | 942.48 | 141372 | 0.01 | 9E+06 | Insect Damaged | 0.20252 | -3.176 | 0.0162 | 0.9641 | 0.0197 |
| 169 | Insect Damaged | 271.63 | 159 | 999.03 | 158845 | 0.01 | 7378.9 | Insect Damaged | 0.33209 | -0.435 | 0.1102 | 0.5934 | 0.2964 |
| 170 | Insect Damaged | 266.22 | 154 | 967.61 | 149012 | 0.01 | 70873 | Insect Damaged | 0.05794 | -0.692 | 0.149 | 0.6612 | 0.1898 |
| 171 | Insect Damaged | 270.83 | 144 | 904.78 | 130288 | 0.01 | 73349 | Insect Damaged | -0.49379 | -1.174 | 0.2383 | 0.6938 | 0.0678 |
| 172 | Insect Damaged | 27762 | 155 | 973.89 | 150954 | 0.01 | 77067 | Insect Damaged | 0.09688 | -0.784 | 0.1326 | 0.6836 | 0.1838 |
| 173 | Insect Damaged | 265.73 | 147 | 923.63 | 135773 | 0.01 | 70612 | Insect Damaged | -0.32855 | -1.029 | 0.2107 | 0.6954 | 0.0939 |
| 174 | Insect Damaged | 277.81 | 160 | 1005.31 | 160850 | 0.01 | 77178 | Insect Damaged | 0.39024 | -0.405 | 0.1012 | 0.5809 | 0.3179 |
| 175 | Insect Damaged | 287.36 | 171 | 1074.42 | 183727 | 0.01 | 82576 | Disease damaged | 1.00107 | 0.1233 | 0.0375 | 0.3448 | 0.6178 |
| 176 | Insect Damaged | 303 | 160 | 1005.31 | 160850 | 0.01 | 91809 | Insect Damaged | 0.3908 | -0.408 | 0.101 | 0.5818 | 0.3172 |
| 177 | Insect Damaged | 286.37 | 151 | 948.76 | 143263 | 0.01 | 82008 | Insect Damaged | -0.10738 | -0.839 | 0.1744 | 0.6839 | 0.1417 |
| 178 | Insect Damaged | 302.95 | 153 | 961.33 | 147083 | 0.01 | 91779 | Insect Damaged | 0.00348 | -0.745 | 0.1568 | 0.6712 | 0.1721 |
| 179 | Insect Damaged | 262.56 | 150 | 942.48 | 141372 | 0.01 | 68938 | Insect Damaged | -0.1631 | -0.884 | 0.1837 | 0.6879 | 0.1284 |
| 180 | Insect Damaged | 289.19 | 143 | 898.5 | 128485 | 0.01 | 83631 | Insect Damaged | -0.54847 | -1.225 | 0.2472 | 0.6922 | 0.0606 |
| 181 | Insect Damaged | 258.04 | 161 | 1011.59 | 162866 | 0.01 | 0 | Insect Damaged | 0.44261 | -0.337 | 0.0954 | 0.5581 | 0.3466 |
| 182 | Insect Damaged | 274.36 | 150 | 942.48 | 141372 | 0.01 | 75273 | Insect Damaged | -0.16286 | -0.886 | 0.1835 | 0.6883 | 0.1283 |
| 183 | Insect Damaged | 305.09 | 168 | 1055.58 | 177337 | 0.01 | 9E+06 | Insect Damaged | 1.19983 | -2.313 | 0.0068 | 0.8697 | 0.1236 |
| 184 | Insect Damaged | 265.1 | 149 | 936.19 | 139493 | 0.01 | 70278 | Insect Damaged | -0.21824 | -0.932 | 0.1926 | 0.6916 | 0.1159 |
| 185 | Insect Damaged | 276.44 | 152 | 955.04 | 145167 | 0.01 | 76419 | Insect Damaged | -0.05237 | -0.79 | 0.1659 | 0.6775 | 0.1566 |
| 186 | Insect Damaged | 302.67 | 145 | 911.06 | 132104 | 0.01 | 91609 | Insect Damaged | -0.43799 | -1.13 | 0.2281 | 0.6965 | 0.0755 |
| 187 | Insect Damaged | 262.98 | 161 | 1011.59 | 162866 | 0.01 | 69158 | Insect Damaged | 0.44534 | -0.355 | 0.0942 | 0.5624 | 0.3435 |
| 188 | Insect Damaged | 273.21 | 150 | 942.48 | 141372 | 0.01 | 74644 | Insect Damaged | -0.16288 | -0.885 | 0.1835 | 0.6882 | 0.1283 |
| 189 | Insect Damaged | 268.03 | 149 | 936.19 | 139493 | 0.01 | 71840 | Insect Damaged | -0.21818 | -0.933 | 0.1925 | 0.6916 | 0.1158 |
| 190 | Insect Damaged | 304.11 | 167 | 1049.29 | 175232 | 0.01 | 92483 | Disease damaged | 0.77913 | -0.072 | 0.0562 | 0.4369 | 0.5069 |
| 191 | Insect Damaged | 288.66 | 156 | 980.18 | 152908 | 0.01 | 83325 | Insect Damaged | 0.16903 | -0.599 | 0.1321 | 0.6405 | 0.2274 |
| 192 | Insect Damaged | 283.4 | 171 | 1074.42 | 183727 | 0.01 | 80316 | Disease damaged | 1.00099 | 0.1239 | 0.0375 | 0.3446 | 0.6179 |
| 193 | Insect Damaged | 276 | 161 | 1011.59 | 162866 | 0.01 | 76176 | Insect Damaged | 0.44561 | -0.356 | 0.094 | 0.5628 | 0.3432 |
| 194 | Insect Damaged | 270.39 | 159 | 99.03 | 158845 | 0.01 | 73111 | Insect Damaged | 0.34279 | -0.46 | 0.1068 | 0.5989 | 0.2942 |
| 195 | Insect Damaged | 261.13 | 166 | 1043.01 | 173139 | 0.01 | 68189 | Disease damaged | 0.72266 | -0.114 | 0.062 | 0.458 | 0.48 |
| 196 | Insect Damaged | 307.68 | 170 | 1068.14 | 181584 | 0.01 | 94667 | Disease damaged | 0.94593 | 0.0721 | 0.0416 | 0.3681 | 0.5903 |
| 197 | Insect Damaged | 297.7 | 153 | 961.33 | 147083 | 0.01 | 88625 | Insect Damaged | 0.00336 | -0.745 | 0.1569 | 0.671 | 0.1722 |
| 198 | Insect Damaged | 279.49 | 169 | 1061.86 | 179454 | 0.01 | 78115 | Disease damaged | 0.8897 | 0.0282 | 0.0463 | 0.39 | 0.5637 |
| 199 | Insect Damaged | 282.14 | 143 | 898.5 | 128485 | 0.01 | 79603 | Insect Damaged | -0.54862 | -1.224 | 0.2474 | 0.6919 | 0.0607 |
| 200 | Insect Damaged | 257194 | 142 | 892.21 | 126694 | 0.01 | 66533 | Insect Damaged | -0.75933 | -2.565 | 0.1207 | 0.8655 | 0.0138 |
| 201 | Disease damaged | 305.6 | 171 | 1074.42 | 183727 | 0.01 | 93391 | Disease damaged | 1.00149 | 0.1205 | 0.0374 | 0.3454 | 0.6172 |
| 202 | Disease damaged | 321.7 | 165 | 1036.73 | 171060 | 0.01 | 103497 | Insect Damaged | 0.66851 | -0.171 | 0.0674 | 0.4826 | 0.45 |
| 203 | Disease damaged | 320.36 | 183 | 1149.82 | 210418 | 0.01 | 102631 | Disease damaged | 1.67073 | 0.6956 | 0.0085 | 0.1317 | 0.8599 |
| 204 | Disease damaged | 327.43 | 169 | 1061.86 | 179454 | 0.01 | 107210 | Disease damaged | 0.89082 | 0.0207 | 0.0461 | 0.3918 | 0.5622 |
| 205 | Disease damaged | 299.54 | 172 | 1080.71 | 185882 | 0.01 | 89724 | Disease damaged | 1.05698 | 0.1696 | 0.0336 | 0.3229 | 0.6435 |
| 206 | Disease damaged | 296.82 | 192 | 1206.37 | 23162 | 0.01 | 88102 | Disease damaged | 1.84352 | 1.1425 | 0.0054 | 0.0724 | 0.9222 |
| 207 | Disease damaged | 321.48 | 187 | 1174.96 | 219717 | 0.01 | 103349 | Disease damaged | 1.89436 | 0.8879 | 0.0049 | 0.0899 | 0.9052 |
| 208 | Disease damaged | 298.63 | 183 | 1149.82 | 210418 | 0.01 | 89180 | Disease damaged | 1.67021 | 0.6991 | 0.0085 | 0.1313 | 0.8602 |
| 209 | Disease damaged | 323.58 | 159 | 999.03 | 158845 | 0.01 | 10474 | Insect Damaged | 0.33218 | -0.437 | 0.1101 | 0.5936 | 0.2963 |
| 210 | Disease damaged | 325.62 | 187 | 1174.96 | 219717 | 0.01 | 106028 | Disease damaged | 1.89446 | 0.8872 | 0.0049 | 0.09 | 0.9052 |
| 211 | Disease damaged | 297.39 | 173 | 1086.99 | 188049 | 0.01 | 0 | Disease damaged | 1.10908 | 0.24 | 0.0303 | 0.2967 | 0.673 |
| 212 | Disease damaged | 321.99 | 186 | 1168.67 | 217373 | 0.01 | 103678 | Disease damaged | 1.83844 | 0.8397 | 0.0056 | 0.0991 | 0.8953 |
| 213 | Disease damaged | 307.67 | 169 | 1061.86 | 179454 | 0.01 | 94661 | Disease damaged | 0.89034 | 0.024 | 0.0461 | 0.391 | 0.5628 |
| 214 | Disease damaged | 300.96 | 185 | 1162.39 | 215042 | 0.01 | 90577 | Disease damaged | 1.78202 | 0.795 | 0.0064 | 0.1088 | 0.8848 |
| 215 | Disease damaged | 288.71 | 1.69 | 1061.86 | 179454 | 0.01 | 81630 | Good | -7.85607 | -8.033 | 0.9845 | 0.0155 | 0 |
| 216 | Disease damaged | 307.12 | 182 | 1143.54 | 208124 | 0.01 | 9E+06 | Insect Damaged | 1.98456 | -1.67 | 0.0025 | 0.6132 | 0.3843 |
| 217 | Disease damaged | 306.11 | 174 | 1093.27 | 190230 | 0.01 | 93703 | Disease damaged | 1.16845 | 0.2648 | 0.0267 | 0.2804 | 0.6929 |
| 218 | Disease damaged | 304.13 | 173 | 1086.99 | 188049 | 0.01 | 92995 | Disease damaged | 1.11276 | 0.2169 | 0.03 | 0.3014 | 0.6687 |
| 219 | Disease damaged | 307.98 | 180 | 1130.97 | 203575 | 0.01 | 94852 | Disease damaged | 1.50294 | 0.5533 | 0.0127 | 0.1724 | 0.815 |
| 220 | Disease damaged | 297.54 | 167 | 1049.29 | 175232 | 0.01 | 88530 | Disease damaged | 0.77898 | -0.071 | 0.0562 | 0.4366 | 0.5071 |
| 221 | Disease damaged | 321.82 | 159 | 999.03 | 158845 | 0.01 | 103568 | Insect Damaged | 0.33587 | -0.46 | 0.1081 | 0.5994 | 0.2925 |
| 222 | Disease damaged | 293.67 | 189 | 1187.52 | 224442 | 0.01 | 86242 | Disease damaged | 2.00561 | 0.9885 | 0.0037 | 0.0735 | 0.9229 |
| 223 | Disease damaged | 322.78 | 177 | 1112.12 | 196846 | 0.01 | 104187 | Disease damaged | 1.33599 | 0.4065 | 0.0186 | 0.2226 | 0.7589 |
| 224 | Disease damaged | 311 | 188 | 1181024 | 222073 | 0.01 | 96721 | Good | -8.66438 | 11.452 | 1 | 0 | 0 |
| 225 | Disease damaged | 299.87 | 190 | 1193.81 | 226823 | 0.01 | 89922 | Disease damaged | 2.06174 | 1.0357 | 0.0032 | 0.0665 | 0.9303 |
| 226 | Disease damaged | 309.71 | 189 | 1187.52 | 224442 | 0.01 | 95920 | Disease damaged | 2.00599 | 0.9861 | 0.0037 | 0.0736 | 0.9227 |
| 227 | Disease damaged | 310.8 | 176 | 1105.84 | 194628 | 0.01 | 96597 | Disease damaged | 1.27997 | 0.3603 | 0.021 | 0.2408 | 0.7382 |
| 228 | Disease damaged | 319.72 | 183 | 1149.82 | 210418 | 0.01 | 102221 | Disease damaged | 1.67071 | 0.6957 | 0.0085 | 0.1317 | 0.8599 |
| 229 | Disease damaged | 314.59 | 178 | 1118.41 | 199076 | 0.01 | 98967 | Disease damaged | 1.39154 | 0.456 | 0.0164 | 0.2047 | 0.7789 |
| 230 | Disease damaged | 299.6 | 181 | 1137.26 | 205843 | 0.01 | 89760 | Disease damaged | 1.55855 | 0.6027 | 0.0111 | 0.1576 | 0.8313 |
| 231 | Disease damaged | 297.58 | 186 | 1168.67 | 217373 | 0.01 | 88554 | Disease damaged | 1.83785 | 0.8436 | 0.0056 | 0.0988 | 0.8956 |
| 232 | Disease damaged | 263.61 | 186 | 1168.67 | 217373 | 0.01 | 69490 | Disease damaged | 1.83712 | 0.8485 | 0.0056 | 0.0983 | 0.8961 |
| 233 | Disease damaged | 306.81 | 167 | 1049.29 | 175232 | 0.01 | 34132 | Disease damaged | 0.77682 | -0.057 | 0.0567 | 0.4334 | 0.5099 |
| 234 | Disease damaged | 314.72 | 176 | 1105.84 | 194628 | 0.01 | 99049 | Disease damaged | 1.28006 | 0.3597 | 0.021 | 0.2409 | 0.7381 |
| 235 | Disease damaged | 295.08 | 187 | 1174.96 | 219717 | 0.01 | 87072 | Disease damaged | 1.89373 | 0.8921 | 0.0049 | 0.0896 | 0.9056 |
| 236 | Disease damaged | 304 | 175 | 1099.56 | 192483 | 0.01 | 92416 | Disease damaged | 1.22419 | 0.3133 | 0.0237 | 0.26 | 0.7163 |
| 237 | Disease damaged | 295.41 | 189 | 1187.52 | 224442 | 0.01 | 87267 | Disease damaged | 2.00565 | 0.9883 | 0.0037 | 0.0735 | 0.9229 |
| 238 | Disease damaged | 272.82 | 159 | 999.03 | 158845 | 0.01 | 74431 | Insect Damaged | 0.33474 | -0.452 | 0.1088 | 0.5975 | 0.2937 |
| 239 | Disease damaged | 275 | 174 | 1093.27 | 190230 | 0.01 | 75625 | Disease damaged | 1.16776 | 0.2695 | 0.0268 | 0.2795 | 0.6938 |
| 240 | Disease damaged | 245.76 | 179 | 1124.69 | 201320 | 0.01 | 60398 | Disease damaged | 1.44582 | 0.514 | 0.0145 | 0.1867 | 0.7989 |
| 241 | Disease damaged | 275.86 | 182 | 1143.54 | 0 | 0.01 | 75625 | Disease damaged | 1.28418 | 0.6646 | 0.0213 | 0.1837 | 0.795 |
| 242 | Disease damaged | 280.56 | 176 | 1105.84 | 194628 | 0.01 | 78714 | Disease damaged | 1.27928 | 0.3649 | 0.0211 | 0.24 | 0.739 |
| 243 | Disease damaged | 277.46 | 181 | 1137.26 | 205843 | 0.01 | 76984 | Disease damaged | 1.55806 | 0.606 | 0.0111 | 0.1572 | 0.8317 |
| 244 | Disease damaged | 273.86 | 169 | 1061.86 | 179454 | 0.01 | 74999 | Disease damaged | 0.88958 | 0.029 | 0.0463 | 0.3898 | 0.5639 |
| 245 | Disease damaged | 299.67 | 180 | 1130.97 | 203575 | 0.01 | 89802 | Disease damaged | 1.50274 | 0.5546 | 0.0127 | 0.1722 | 0.8152 |
| 246 | Disease damaged | 320.11 | 178 | 1118.41 | 199076 | 0.01 | 102470 | Disease damaged | 1.39167 | 0.4551 | 0.0164 | 0.2049 | 0.7787 |
| 247 | Disease damaged | 295.7 | 182 | 1124.69 | 201320 | 0.01 | 87438 | Disease damaged | 1.60368 | 0.6516 | 0.01 | 0.1451 | 0.8449 |
| 248 | Disease damaged | 298.14 | 176 | 1105.84 | 194628 | 0.01 | 88887 | Disease damaged | 1.27967 | 0.3623 | 0.021 | 0.2404 | 0.7385 |
| 249 | Disease damaged | 298.41 | 192 | 1206.37 | 23162 | 0.01 | 89049 | Disease damaged | 1.84356 | 1.1423 | 0.0054 | 0.0724 | 0.9222 |
| 250 | Disease damaged | 300.98 | 169 | 1061.86 | 2E+06 | 0.01 | 90589 | Disease damaged | 3.44778 | -0.054 | 0.0001 | 0.0591 | 0.9408 |
| 251 | Disease damaged | 298.57 | 183 | 1149.82 | 210418 | 0.01 | 89144 | Disease damaged | 1.67021 | 0.6991 | 0.0085 | 0.1313 | 0.8602 |
| 252 | Disease damaged | 295.58 | 176 | 1105.84 | 194628 | 0.01 | 87368 | Disease damaged | 1.27961 | 0.3627 | 0.021 | 0.2404 | 0.7386 |
| 253 | Disease damaged | 295.78 | 167 | 1049.29 | 175232 | 0.01 | 87486 | Disease damaged | 0.77894 | -0.07 | 0.0563 | 0.4366 | 0.5072 |
| 254 | Disease damaged | 275.49 | 184 | 1156.11 | 212724 | 0.01 | 75895 | Disease damaged | 1.72557 | 0.7506 | 0.0074 | 0.1192 | 0.8734 |
| 255 | Disease damaged | 300.16 | 173 | 1086.99 | 188049 | 0.01 | 90096 | Disease damaged | 1.11264 | 0.2176 | 0.03 | 0.3012 | 0.6688 |
| 256 | Disease damaged | 288.67 | 170 | 1068.14 | 181584 | 0.01 | 83330 | Disease damaged | 0.94549 | 0.075 | 0.0417 | 0.3674 | 0.5909 |
| 257 | Disease damaged | 283.98 | 185 | 1162.39 | 215042 | 0.01 | 80645 | Disease damaged | 1.78164 | 0.7975 | 0.0065 | 0.1085 | 0.885 |
| 258 | Disease damaged | 311.41 | 160 | 1005.31 | 160850 | 0.01 | 96976 | Insect Damaged | 0.391 | -0.41 | 0.1009 | 0.5822 | 0.317 |
| 259 | Disease damaged | 319.69 | 169 | 1061.86 | 179454 | 0.01 | 102202 | Disease damaged | 0.89063 | 0.022 | 0.0461 | 0.3915 | 0.5624 |
| 260 | Disease damaged | 281.91 | 189 | 1187.52 | 224442 | 0.01 | 79473 | Disease damaged | 2.00535 | 0.9903 | 0.0037 | 0.0734 | 0.923 |
| 261 | Disease damaged | 284.28 | 163 | 1024.16 | 166938 | 0.01 | 80815 | Insect Damaged | 0.55668 | -0.261 | 0.0803 | 0.5239 | 0.3959 |
| 262 | Disease damaged | 314.17 | 180 | 1130.97 | 203575 | 0.01 | 98703 | Disease damaged | 1.50309 | 0.5523 | 0.0127 | 0.1725 | 0.8148 |
| 263 | Disease damaged | 297.51 | 161 | 1011.59 | 162866 | 0.01 | 88512 | Insect Damaged | 0.44609 | -0.359 | 0.0938 | 0.5636 | 0.3426 |
| 264 | Disease damaged | 287.25 | 176 | 1105.84 | 194628 | 0.01 | 82513 | Disease damaged | 1.27942 | 0.364 | 0.0211 | 0.2401 | 0.7388 |
| 265 | Disease damaged | 279.3 | 183 | 1149.82 | 210418 | 0.01 | 78008 | Disease damaged | 1.66978 | 0.702 | 0.0085 | 0.131 | 0.8605 |
| 266 | Disease damaged | 300.69 | 193 | 1212.65 | 234042 | 0.01 | 90414 | Disease damaged | 2.22985 | 1.1799 | 0.0021 | 0.049 | 0.9489 |
| 267 | Disease damaged | 312.62 | 182 | 1143.54 | 208124 | 0.01 | 97731 | Disease damaged | 1.61469 | 0.6488 | 0.0097 | 0.1442 | 0.8461 |
| 268 | Disease damaged | 294.14 | 178 | 1118.41 | 199076 | 0.01 | 86518 | Disease damaged | 1.39106 | 0.4592 | 0.0164 | 0.2042 | 0.7794 |
| 269 | Disease damaged | 283 | 167 | 1049.29 | 175232 | 0.01 | 80089 | Disease damaged | 0.77865 | -0.069 | 0.0563 | 0.4361 | 0.5076 |
| 270 | Disease damaged | 253.05 | 187 | 1174.96 | 219717 | 0.01 | 64034 | Disease damaged | 1.89284 | 0.898 | 0.0049 | 0.0891 | 0.906 |
| 271 | Disease damaged | 275.14 | 170 | 1068.14 | 181584 | 0.01 | 75702 | Disease damaged | 0.9452 | 0.077 | 0.0417 | 0.367 | 0.5913 |
| 272 | Disease damaged | 281.62 | 190 | 1193.81 | 226823 | 0.01 | 79310 | Disease damaged | 2.06133 | 1.0384 | 0.0032 | 0.0664 | 0.9305 |
| 273 | Disease damaged | 301.02 | 173 | 1086.99 | 188049 | 0.01 | 90613 | Disease damaged | 1.11266 | 0.2175 | 0.03 | 0.3013 | 0.6688 |
| 274 | Disease damaged | 273.88 | 186 | 1168.67 | 217373 | 0.01 | 81642 | Disease damaged | 1.83759 | 0.8454 | 0.0056 | 0.0986 | 0.8958 |
| 275 | Disease damaged | 273.88 | 163 | 1036.73 | 171060 | 0.01 | 75010 | Insect Damaged | 0.56287 | -0.26 | 0.0794 | 0.5227 | 0.398 |
| 276 | Disease damaged | 263.05 | 167 | 1049.29 | 175232 | 0.01 | 69195 | Disease damaged | 0.77823 | -0.066 | 0.0564 | 0.4354 | 0.5082 |
| 277 | Disease damaged | 273.11 | 191 | 1200.09 | 229217 | 0.01 | 75010 | Disease damaged | 2.11718 | 1.0877 | 0.0028 | 0.0599 | 0.9373 |
| 278 | Disease damaged | 288.15 | 173 | 1086.99 | 188049 | 0.01 | 83030 | Disease damaged | 1.11237 | 0.2195 | 0.03 | 0.3009 | 0.6691 |
| 279 | Disease damaged | 282.94 | 186 | 1168.67 | 217373 | 0.01 | 80055 | Disease damaged | 1.83753 | 0.8458 | 0.0056 | 0.0986 | 0.8958 |
| 280 | Disease damaged | 298.06 | 189 | 1187.52 | 224442 | 0.01 | 88840 | Disease damaged | 2.00571 | 0.9879 | 0.0037 | 0.0735 | 0.9228 |
| 281 | Disease damaged | 282.01 | 170 | 1068.14 | 181584 | 0.01 | 0 | Disease damaged | 0.94219 | 0.0957 | 0.0422 | 0.3627 | 0.5951 |
| 282 | Disease damaged | 275.06 | 192 | 1206.37 | 231623 | 0.01 | 75658 | Disease damaged | 2.17323 | 1.1356 | 0.0024 | 0.0541 | 0.9435 |
| 283 | Disease damaged | 294.15 | 179 | 1124.69 | 201320 | 0.01 | 86524 | Disease damaged | 1.44683 | 0.5073 | 0.0144 | 0.1877 | 0.7979 |
| 284 | Disease damaged | 272.7 | 171 | 1074.42 | 183727 | 0.01 | 74365 | Disease damaged | 1.00076 | 0.1254 | 0.0375 | 0.3443 | 0.6182 |
| 285 | Disease damaged | 300.14 | 160 | 1005.31 | 160850 | 0.01 | 90084 | Insect Damaged | 0.39074 | -0.408 | 0.101 | 0.5817 | 0.3173 |
| 286 | Disease damaged | 288.19 | 174 | 1093.27 | 190230 | 0.01 | 83053 | Disease damaged | 1.16804 | 0.2676 | 0.0267 | 0.2798 | 0.6934 |
| 287 | Disease damaged | 294.9 | 170 | 1068.14 | 181584 | 0.01 | 86966 | Disease damaged | 0.94563 | 0.0741 | 0.0417 | 0.3677 | 0.5907 |
| 288 | Disease damaged | 311.57 | 183 | 1149.82 | 210418 | 0.01 | 97076 | Disease damaged | 1.67051 | 0.6971 | 0.0085 | 0.1315 | 0.86 |
| 289 | Disease damaged | 297.17 | 174 | 1093.27 | 190230 | 0.01 | 88310 | Disease damaged | 1.16825 | 0.2662 | 0.0267 | 0.2801 | 0.6932 |
| 290 | Disease damaged | 280.14 | 169 | 1061.86 | 179454 | 0.01 | 78478 | Disease damaged | 0.88971 | 0.0281 | 0.0463 | 0.3901 | 0.5637 |
| 291 | Disease damaged | 306.8 | 170 | 1068.14 | 181584 | 0.01 | 94126 | Disease damaged | 0.94591 | 0.0722 | 0.0416 | 0.3681 | 0.5903 |
| 292 | Disease damaged | 269.98 | 158 | 992.74 | 156853 | 0.01 | 72889 | Insect Damaged | 0.27931 | -0.5 | 0.1165 | 0.6129 | 0.2706 |
| 293 | Disease damaged | 307.16 | 186 | 1168.67 | 217373 | 0.01 | 94347 | Disease damaged | 1.83808 | 0.8421 | 0.0056 | 0.0989 | 0.8955 |
| 294 | Disease damaged | 312.99 | 190 | 1193.81 | 226823 | 0.01 | 97963 | Disease damaged | 2.06205 | 1.0336 | 0.0032 | 0.0666 | 0.9302 |
| 295 | Disease damaged | 299.6 | 178 | 1118.41 | 199076 | 0.01 | 87960 | Disease damaged | 1.39111 | 0.4588 | 0.0164 | 0.2043 | 0.7793 |
| 296 | Disease damaged | 276.12 | 187 | 1174.96 | 219717 | 0.01 | 76242 | Disease damaged | 1.89331 | 0.8949 | 0.0049 | 0.0893 | 0.9058 |
| 297 | Disease damaged | 287.71 | 172 | 1080.71 | 185882 | 0.01 | 82777 | Disease damaged | 1.05671 | 0.1714 | 0.0336 | 0.3225 | 0.6439 |
| 298 | Disease damaged | 297.5 | 185 | 1162.39 | 215042 | 0.01 | 88506 | Disease damaged | 1.78194 | 0.7955 | 0.0065 | 0.1087 | 0.8848 |
| 299 | Disease damaged | 306.11 | 168 | 1055.58 | 177337 | 0.01 | 93703 | Disease damaged | 0.83473 | -0.024 | 0.051 | 0.414 | 0.535 |
| 300 | Disease damaged | 323.06 | 186 | 1168.67 | 217373 | 0.01 | 104368 | Disease damaged | 1.83847 | 0.8395 | 0.0056 | 0.0991 | 0.8953 |

A = Amplitude, F = Frequency, I = Intensity, T = Period, V = Velocity, ʎ = Wavelength,

‘Pred. Yam quality’ is the predicted yam quality class.

Quality class: 1= Good, 2 = Insect damaged and 3 = Diseased damaged

‘DSF1’ is ‘Discriminant Scores from Function (Model) 1’.

‘DSF2’ is ‘Discriminant Scores from Function (Model) 2’,

‘PMC1’ is the ‘Probabilities of Membership in Yam Quality Class 1’.

‘PMC2’ is the ‘Probabilities of Membership in Yam Quality Class 2’.

‘PMC3’ is the ‘Probabilities of Membership in Yam Quality Class 3’

Table S8: Acoustic properties of yellow yam qualities for surface impact sound technique and their classification training result using machine learning algorithm

| SN | Yam Quality | A | T | F | W | V | I | Pred. Yam quality | DSF1 | DSF2 | PMC1 | PMC2 | PMC3 |
| --- | --- | --- | --- | --- | --- | --- | --- | --- | --- | --- | --- | --- | --- |
| 1 | Good | 152.110 | 0.010 | 182.000 | 1143.540 | 20810.280 | 2317.450 | Good | 1.528 | -0.119 | 0.572 | 0.423 | 0.005 |
| 2 | Good | 152.110 | 0.010 | 182.000 | 1143.540 | 20810.280 | 23137.450 | Good | 1.538 | -0.012 | 0.581 | 0.414 | 0.005 |
| 3 | Good | 143.660 | 0.010 | 144.000 | 904.780 | 130288.130 | 20638.200 | Insect Damaged | 0.339 | -0.081 | 0.408 | 0.512 | 0.081 |
| 4 | Good | 161.320 | 0.000 | 252.000 | 1583.360 | 399007.400 | 26024.140 | Good | 3.495 | 0.378 | 0.792 | 0.208 | 0.000 |
| 5 | Good | 143.910 | 0.010 | 144.000 | 904.780 | 130288.130 | 20710.090 | Insect Damaged | 0.339 | -0.080 | 0.408 | 0.512 | 0.081 |
| 6 | Good | 141.610 | 0.010 | 141.000 | 885.930 | 124916.010 | 20053.390 | Insect Damaged | 0.247 | -0.091 | 0.390 | 0.512 | 0.098 |
| 7 | Good | 140.050 | 0.010 | 144.000 | 904.780 | 130288.130 | 19614.000 | Insect Damaged | 0.338 | -0.086 | 0.407 | 0.512 | 0.081 |
| 8 | Good | 143.100 | 0.010 | 180.000 | 1130.970 | 203575.200 | 20477.610 | Good | 1.411 | 0.033 | 0.569 | 0.425 | 0.007 |
| 9 | Good | 143.290 | 0.010 | 180.000 | 1130.970 | 203575.200 | 20532.020 | Good | 1.411 | 0.033 | 0.569 | 0.425 | 0.007 |
| 10 | Good | 144.210 | 0.010 | 180.000 | 1130.970 | 203575.200 | 20796.520 | Good | 1.411 | 0.034 | 0.569 | 0.425 | 0.007 |
| 11 | Good | 136.230 | 0.010 | 144.000 | 904.780 | 130288.130 | 18558.610 | Insect Damaged | 0.338 | -0.092 | 0.407 | 0.512 | 0.081 |
| 12 | Good | 123.630 | 0.000 | 252.000 | 1583.360 | 399007.400 | 15284.380 | Good | 3.490 | 0.322 | 0.789 | 0.211 | 0.000 |
| 13 | Good | 164.110 | 0.010 | 180.000 | 1130.970 | 203575.200 | 26932.090 | Good | 1.414 | 0.066 | 0.571 | 0.422 | 0.007 |
| 14 | Good | 116.900 | 0.010 | 108.000 | 678.580 | 73287.070 | 13665.610 | Disease damaged | -0.781 | -0.169 | 0.145 | 0.310 | 0.545 |
| 15 | Good | 134.050 | 0.010 | 108.000 | 678.580 | 73287.070 | 17969.400 | Disease damaged | -0.779 | -0.146 | 0.146 | 0.310 | 0.544 |
| 16 | Good | 162.080 | 0.010 | 143.000 | 898.500 | 128484.860 | 26269.930 | Insect Damaged | 0.311 | -0.054 | 0.404 | 0.510 | 0.086 |
| 17 | Good | 151.430 | 0.010 | 144.000 | 904.780 | 130288.130 | 22931.040 | Insect Damaged | 0.340 | -0.069 | 0.408 | 0.511 | 0.081 |
| 18 | Good | 142.600 | 0.010 | 144.000 | 904.780 | 130288.130 | 20334.760 | Insect Damaged | 0.338 | -0.082 | 0.407 | 0.512 | 0.081 |
| 19 | Good | 101.900 | 0.010 | 180.000 | 1130.970 | 203575.200 | 10383.610 | Good | 1.406 | -0.020 | 0.564 | 0.429 | 0.007 |
| 20 | Good | 131.030 | 0.010 | 180.000 | 1130.970 | 203575.200 | 17168.870 | Good | 1.410 | 0.015 | 0.567 | 0.426 | 0.007 |
| 21 | Good | 174.140 | 0.010 | 180.000 | 1130.970 | 203575.200 | 30324.740 | Good | 1.416 | 0.084 | 0.573 | 0.421 | 0.007 |
| 22 | Good | 158.620 | 0.010 | 182.000 | 1143.540 | 20810.280 | 25160.300 | Good | 1.539 | -0.002 | 0.581 | 0.414 | 0.005 |
| 23 | Good | 146.330 | 0.010 | 181.000 | 1137.260 | 205843.430 | 21412.470 | Good | 1.443 | 0.039 | 0.573 | 0.421 | 0.006 |
| 24 | Good | 160.150 | 0.000 | 288.000 | 1809.560 | 521152.520 | 25648.020 | Good | 4.514 | 0.561 | 0.864 | 0.136 | 0.000 |
| 25 | Good | 122.730 | 0.010 | 182.000 | 1143.540 | 20810.280 | 15062.650 | Good | 1.534 | -0.054 | 0.577 | 0.418 | 0.005 |
| 26 | Good | 134.150 | 0.000 | 218.000 | 1369.730 | 298602.100 | 17996.220 | Good | 2.516 | 0.176 | 0.697 | 0.302 | 0.000 |
| 27 | Good | 158.720 | 0.010 | 144.000 | 904.780 | 130288.130 | 25192.030 | Insect Damaged | 0.341 | -0.057 | 0.409 | 0.510 | 0.081 |
| 28 | Good | 150.700 | 0.010 | 180.000 | 1130.970 | 203575.200 | 22710.490 | Good | 1.412 | 0.044 | 0.570 | 0.424 | 0.007 |
| 29 | Good | 138.300 | 0.000 | 252.000 | 1583.360 | 399007.400 | 19126.890 | Good | 3.491 | 0.342 | 0.790 | 0.210 | 0.000 |
| 30 | Good | 152.150 | 0.010 | 180.000 | 1130.970 | 203575.200 | 23149.620 | Good | 1.414 | 0.044 | 0.570 | 0.424 | 0.007 |
| 31 | Good | 142.220 | 0.000 | 216.000 | 1357.170 | 293148.290 | 20226.530 | Good | 2.460 | 0.179 | 0.692 | 0.308 | 0.001 |
| 32 | Good | 152.110 | 0.010 | 182.000 | 1143.540 | 208124.230 | 23137.450 | Good | 1.472 | 0.054 | 0.577 | 0.417 | 0.006 |
| 33 | Good | 143.220 | 0.000 | 252.000 | 1583.360 | 399007.400 | 20511.970 | Good | 3.492 | 0.350 | 0.790 | 0.210 | 0.000 |
| 34 | Good | 151.110 | 0.010 | 141.000 | 885.930 | 124916.010 | 22834.230 | Insect Damaged | 0.248 | -0.076 | 0.391 | 0.511 | 0.098 |
| 35 | Good | 143.110 | 0.010 | 108.000 | 678.580 | 73287.070 | 0.000 | Disease damaged | -0.788 | -0.238 | 0.142 | 0.311 | 0.547 |
| 36 | Good | 141.110 | 0.010 | 180.000 | 1130.970 | 203575.200 | 19912.030 | Good | 1.411 | 0.030 | 0.568 | 0.425 | 0.007 |
| 37 | Good | 140.260 | 0.000 | 208.000 | 1306.900 | 271835.730 | 19672.870 | Good | 2.228 | 0.141 | 0.666 | 0.333 | 0.001 |
| 38 | Good | 143.100 | 0.010 | 140.000 | 879.640 | 123150.430 | 20477.610 | Insect Damaged | 0.217 | -0.091 | 0.384 | 0.511 | 0.105 |
| 39 | Good | 143.290 | 0.010 | 180.000 | 1130.970 | 203575.200 | 20532.020 | Good | 1.411 | 0.033 | 0.569 | 0.425 | 0.007 |
| 40 | Good | 144.210 | 0.000 | 250.000 | 1570.800 | 392699.080 | 20769.520 | Good | 3.435 | 0.341 | 0.786 | 0.214 | 0.000 |
| 41 | Good | 136.220 | 0.000 | 210.000 | 1319.470 | 277088.470 | 18555.890 | Good | 2.286 | 0.144 | 0.672 | 0.327 | 0.001 |
| 42 | Good | 152.210 | 0.010 | 141.000 | 885.930 | 124916.010 | 23167.880 | Insect Damaged | 0.249 | -0.075 | 0.391 | 0.511 | 0.098 |
| 43 | Good | 149.230 | 0.010 | 160.000 | 1005.310 | 160849.540 | 2226959 | Good | 1.907 | 11.319 | 0.975 | 0.024 | 0.001 |
| 44 | Good | 199.370 | 0.000 | 216.000 | 1357.170 | 293148.290 | 39748.400 | Good | 2.469 | 0.280 | 0.699 | 0.301 | 0.001 |
| 45 | Good | 138.770 | 0.010 | 160.000 | 1005.310 | 160849.540 | 19257.110 | Good | 0.818 | -0.042 | 0.487 | 0.486 | 0.027 |
| 46 | Good | 139.080 | 0.010 | 146.000 | 917.350 | 133932.380 | 19343.250 | Insect Damaged | 0.398 | -0.082 | 0.418 | 0.511 | 0.071 |
| 47 | Good | 142.210 | 0.010 | 136.000 | 854.510 | 116213.800 | 20237.910 | Insect Damaged | 0.095 | -0.101 | 0.359 | 0.506 | 0.135 |
| 48 | Good | 136.260 | 0.010 | 108.000 | 678.580 | 73287.070 | 18566.790 | Disease damaged | -0.779 | -0.143 | 0.146 | 0.310 | 0.544 |
| 49 | Good | 148.260 | 0.010 | 120.000 | 753.980 | 90477.870 | 21981.030 | Insect Damaged | -0.398 | -0.118 | 0.241 | 0.428 | 0.331 |
| 50 | Good | 144.270 | 0.010 | 141.000 | 885.930 | 124916.010 | 20813.830 | Insect Damaged | 0.248 | -0.087 | 0.390 | 0.512 | 0.098 |
| 51 | Good | 146.220 | 0.010 | 160.000 | 1005.310 | 160849.540 | 213802.290 | Good | 0.914 | 0.959 | 0.568 | 0.409 | 0.022 |
| 52 | Good | 143.260 | 0.000 | 216.000 | 1357.170 | 293148.290 | 20523.430 | Good | 2.460 | 0.180 | 0.692 | 0.308 | 0.001 |
| 53 | Good | 138.290 | 0.000 | 250.000 | 1570.800 | 392699.080 | 19124.120 | Good | 3.434 | 0.332 | 0.785 | 0.215 | 0.000 |
| 54 | Good | 129.990 | 0.010 | 140.000 | 879.650 | 123150.430 | 21397.840 | Insect Damaged | 0.218 | -0.087 | 0.384 | 0.511 | 0.105 |
| 55 | Good | 146.280 | 0.010 | 182.000 | 1143.540 | 208124.230 | 24404.690 | Good | 1.472 | 0.060 | 0.578 | 0.416 | 0.006 |
| 56 | Good | 156.220 | 0.010 | 182.000 | 945.980 | 176539.540 | 22019.590 | Good | 1.483 | 0.040 | 0.578 | 0.417 | 0.006 |
| 57 | Good | 148.390 | 0.010 | 168.000 | 1055.580 | 177336.620 | 22019.590 | Good | 1.076 | -0.030 | 0.523 | 0.463 | 0.015 |
| 58 | Good | 149.110 | 0.010 | 149.000 | 936.190 | 139493.000 | 22233.790 | Insect Damaged | 0.490 | -0.059 | 0.435 | 0.507 | 0.058 |
| 59 | Good | 147.990 | 0.010 | 160.000 | 1005.310 | 160849.540 | 21901.040 | Good | 0.820 | -0.029 | 0.488 | 0.485 | 0.027 |
| 60 | Good | 158.220 | 0.010 | 190.000 | 1193.810 | 226822.990 | 25033.570 | Good | 1.707 | 0.094 | 0.607 | 0.390 | 0.003 |
| 61 | Good | 145.370 | 0.010 | 145.000 | 911.060 | 132103.970 | 21132.440 | Insect Damaged | 0.369 | -0.075 | 0.413 | 0.511 | 0.076 |
| 62 | Good | 129.980 | 0.010 | 145.000 | 911.060 | 132103.970 | 16894.800 | Insect Damaged | 0.367 | -0.097 | 0.412 | 0.513 | 0.076 |
| 63 | Good | 148.410 | 0.010 | 182.000 | 1143.540 | 208124.230 | 22025.520 | Good | 1.471 | 0.048 | 0.577 | 0.417 | 0.006 |
| 64 | Good | 110.320 | 0.010 | 144.000 | 904.780 | 130288.130 | 21969.170 | Insect Damaged | 0.339 | -0.075 | 0.408 | 0.511 | 0.081 |
| 65 | Good | 148.220 | 0.010 | 145.000 | 911.060 | 132103.970 | 21969.170 | Insect Damaged | 0.369 | -0.071 | 0.414 | 0.511 | 0.076 |
| 66 | Good | 141.110 | 0.010 | 145.000 | 911.060 | 132103.970 | 19912.030 | Insect Damaged | 0.368 | -0.081 | 0.413 | 0.511 | 0.076 |
| 67 | Good | 142.370 | 0.010 | 141.000 | 885.950 | 124916.010 | 20269.220 | Insect Damaged | 0.247 | -0.090 | 0.390 | 0.512 | 0.098 |
| 68 | Good | 147.290 | 0.010 | 145.000 | 911.060 | 132103.920 | 21694.340 | Insect Damaged | 0.369 | -0.072 | 0.414 | 0.511 | 0.076 |
| 69 | Good | 149.220 | 0.010 | 143.000 | 898.500 | 128484.860 | 19046.760 | Insect Damaged | 0.307 | -0.091 | 0.401 | 0.512 | 0.086 |
| 70 | Good | 138.010 | 0.010 | 148.000 | 911.060 | 132103.970 | 21924.720 | Insect Damaged | 0.456 | -0.057 | 0.430 | 0.508 | 0.062 |
| 71 | Good | 148.070 | 0.010 | 139.000 | 873.360 | 121397.420 | 21924.720 | Insect Damaged | 0.187 | -0.086 | 0.379 | 0.510 | 0.112 |
| 72 | Good | 142.370 | 0.010 | 180.000 | 1130.970 | 203575.200 | 20269.220 | Good | 1.411 | 0.032 | 0.569 | 0.425 | 0.007 |
| 73 | Good | 123.620 | 0.010 | 182.000 | 1143.540 | 208124.230 | 15281.900 | Good | 1.468 | 0.013 | 0.574 | 0.420 | 0.006 |
| 74 | Good | 171.150 | 0.010 | 145.000 | 911.060 | 132103.970 | 29292.320 | Insect Damaged | 0.373 | -0.033 | 0.417 | 0.508 | 0.075 |
| 75 | Good | 164.090 | 0.010 | 144.000 | 904.780 | 130288.130 | 26925.530 | Insect Damaged | 0.341 | -0.048 | 0.410 | 0.509 | 0.080 |
| 76 | Good | 116.940 | 0.010 | 146.000 | 917.350 | 133932.380 | 13674.960 | Insect Damaged | 0.396 | -0.112 | 0.416 | 0.513 | 0.071 |
| 77 | Good | 147.650 | 0.010 | 182.000 | 1143.540 | 208124.230 | 21800.520 | Good | 1.471 | 0.047 | 0.577 | 0.417 | 0.006 |
| 78 | Good | 162.880 | 0.010 | 182.000 | 1143.540 | 208124.230 | 26529.890 | Good | 1.473 | 0.071 | 0.579 | 0.416 | 0.006 |
| 79 | Good | 162.880 | 0.010 | 145.000 | 911.060 | 132103.970 | 26529.890 | Insect Damaged | 0.371 | -0.047 | 0.415 | 0.509 | 0.075 |
| 80 | Good | 134.020 | 0.010 | 180.000 | 1130.970 | 203575.200 | 17961.360 | Good | 1.410 | 0.020 | 0.568 | 0.426 | 0.007 |
| 81 | Good | 134.000 | 0.010 | 144.000 | 904.780 | 130288.130 | 17961.380 | Insect Damaged | 0.337 | -0.095 | 0.406 | 0.513 | 0.081 |
| 82 | Good | 181.620 | 0.000 | 72.000 | 452.390 | 32572.030 | 32985.820 | Disease damaged | -1.468 | -0.755 | 0.035 | 0.121 | 0.844 |
| 83 | Good | 171.220 | 0.010 | 145.000 | 911.060 | 132103.970 | 29316.290 | Insect Damaged | 0.373 | -0.032 | 0.417 | 0.508 | 0.075 |
| 84 | Good | 151.440 | 0.010 | 145.000 | 911.060 | 132103.970 | 22934.070 | Insect Damaged | 0.370 | -0.066 | 0.414 | 0.510 | 0.076 |
| 85 | Good | 142.650 | 0.010 | 146.000 | 917.350 | 133932.380 | 26349.020 | Insect Damaged | 0.402 | -0.046 | 0.421 | 0.509 | 0.071 |
| 86 | Good | 101.900 | 0.010 | 145.000 | 911.060 | 132103.970 | 10383.610 | Insect Damaged | 0.364 | -0.131 | 0.409 | 0.515 | 0.076 |
| 87 | Good | 131.030 | 0.010 | 144.000 | 904.780 | 32572.030 | 17168.860 | Insect Damaged | 0.372 | -0.133 | 0.410 | 0.515 | 0.075 |
| 88 | Good | 17414.000 | 0.010 | 145.000 | 911.060 | 132103.970 | 30324.790 | Insect Damaged | 0.252 | 0.330 | 0.416 | 0.484 | 0.099 |
| 89 | Good | 158.620 | 0.010 | 182.000 | 1143.540 | 208124.230 | 25160.300 | Good | 1.472 | 0.064 | 0.578 | 0.416 | 0.006 |
| 90 | Good | 160.080 | 0.010 | 149.000 | 956.190 | 139493.000 | 25625.610 | Insect Damaged | 0.492 | -0.042 | 0.437 | 0.506 | 0.058 |
| 91 | Good | 122.730 | 0.010 | 145.000 | 911.060 | 132103.970 | 15062.650 | Insect Damaged | 0.366 | -0.107 | 0.411 | 0.513 | 0.076 |
| 92 | Good | 134.150 | 0.010 | 149.000 | 936.190 | 139493.000 | 17996.220 | Insect Damaged | 0.488 | -0.081 | 0.433 | 0.509 | 0.058 |
| 93 | Good | 154.720 | 0.010 | 145.000 | 911.060 | 132103.970 | 23938.280 | Insect Damaged | 0.370 | -0.060 | 0.414 | 0.510 | 0.076 |
| 94 | Good | 158.650 | 0.000 | 324.000 | 2035.750 | 659583.660 | 25169.820 | Good | 5.523 | 0.756 | 0.914 | 0.086 | 0.000 |
| 95 | Good | 151.900 | 0.010 | 145.000 | 911.060 | 132103.970 | 23073.610 | Insect Damaged | 0.370 | -0.065 | 0.414 | 0.510 | 0.076 |
| 96 | Good | 150.700 | 0.010 | 144.000 | 904.780 | 130288.130 | 22710.490 | Insect Damaged | 0.339 | -0.070 | 0.408 | 0.511 | 0.081 |
| 97 | Good | 138.290 | 0.010 | 146.000 | 917.350 | 133932.380 | 19124.120 | Insect Damaged | 0.398 | -0.083 | 0.418 | 0.511 | 0.071 |
| 98 | Good | 173.290 | 0.010 | 145.000 | 911.060 | 132103.970 | 30029.420 | Insect Damaged | 0.373 | -0.029 | 0.417 | 0.508 | 0.075 |
| 99 | Good | 163.080 | 0.010 | 149.000 | 936.190 | 139493.000 | 26585.300 | Insect Damaged | 0.492 | -0.036 | 0.437 | 0.505 | 0.058 |
| 100 | Good | 152.110 | 0.010 | 170.000 | 1068.140 | 181584.060 | 23137.450 | Good | 1.118 | 0.010 | 0.531 | 0.456 | 0.014 |
| 101 | Insect Damaged | 107.230 | 0.010 | 71.000 | 446.110 | 31673.540 | 11498.270 | Disease damaged | -1.835 | -0.379 | 0.015 | 0.056 | 0.929 |
| 102 | Insect Damaged | 107.100 | 0.000 | 71.000 | 446.110 | 31673.540 | 11470.410 | Disease damaged | -1.522 | -0.848 | 0.031 | 0.111 | 0.858 |
| 103 | Insect Damaged | 111.670 | 0.010 | 120.000 | 753.980 | 90477.870 | 12470.190 | Insect Damaged | -0.403 | -0.168 | 0.238 | 0.429 | 0.332 |
| 104 | Insect Damaged | 126.750 | 0.010 | 180.000 | 1130.970 | 203575.200 | 16060.490 | Good | 1.409 | 0.010 | 0.567 | 0.426 | 0.007 |
| 105 | Insect Damaged | 92.670 | 0.000 | 215.000 | 1350.880 | 290440.240 | 8587.730 | Good | 2.426 | 0.113 | 0.684 | 0.315 | 0.001 |
| 106 | Insect Damaged | 110.720 | 0.010 | 160.000 | 100849.54 | 160849.540 | 12258.920 | Insect Damaged | 0.632 | -1.771 | 0.347 | 0.615 | 0.038 |
| 107 | Insect Damaged | 120.320 | 0.010 | 145.000 | 911.060 | 132103.970 | 14476.900 | Insect Damaged | 0.366 | -0.110 | 0.411 | 0.513 | 0.076 |
| 108 | Insect Damaged | 100.690 | 0.010 | 146.000 | 917.350 | 133932.380 | 10138.480 | Insect Damaged | 0.394 | -0.130 | 0.414 | 0.514 | 0.071 |
| 109 | Insect Damaged | 110.720 | 0.000 | 74.000 | 464.960 | 34406.720 | 12258.920 | Disease damaged | -1.521 | -0.702 | 0.031 | 0.109 | 0.860 |
| 110 | Insect Damaged | 120.300 | 0.000 | 36.000 | 226.190 | 8143.010 | 14472.090 | Disease damaged | -2.583 | -0.923 | 0.002 | 0.013 | 0.985 |
| 111 | Insect Damaged | 92.650 | 0.000 | 73.000 | 458.670 | 33483.090 | 8584.020 | Disease damaged | -1.466 | -0.853 | 0.035 | 0.123 | 0.842 |
| 112 | Insect Damaged | 110.750 | 0.010 | 145.000 | 911.060 | 132103.970 | 12265.560 | Insect Damaged | 0.373 | -0.134 | 0.410 | 0.515 | 0.075 |
| 113 | Insect Damaged | 120.110 | 0.010 | 146.000 | 917.350 | 133932.380 | 14426.410 | Insect Damaged | 0.396 | -0.108 | 0.416 | 0.513 | 0.071 |
| 114 | Insect Damaged | 110.000 | 0.010 | 145.000 | 911.060 | 132103.970 | 12100.000 | Insect Damaged | 0.373 | -0.134 | 0.410 | 0.515 | 0.075 |
| 115 | Insect Damaged | 149.010 | 0.010 | 108.000 | 678.580 | 73287.070 | 22203.980 | Disease damaged | -0.777 | -0.124 | 0.147 | 0.310 | 0.544 |
| 116 | Insect Damaged | 151.000 | 0.010 | 180.000 | 1130.970 | 203575.200 | 22801.000 | Good | 1.412 | 0.045 | 0.570 | 0.424 | 0.007 |
| 117 | Insect Damaged | 152.070 | 0.010 | 144.000 | 904.780 | 130288.130 | 23125.280 | Insect Damaged | 0.340 | -0.068 | 0.409 | 0.511 | 0.081 |
| 118 | Insect Damaged | 150.090 | 0.010 | 144.000 | 904.780 | 130288.130 | 22527.010 | Insect Damaged | 0.339 | -0.071 | 0.408 | 0.511 | 0.081 |
| 119 | Insect Damaged | 152.070 | 0.010 | 144.000 | 904.780 | 130288.130 | 23125.280 | Insect Damaged | 0.340 | -0.068 | 0.409 | 0.511 | 0.081 |
| 120 | Insect Damaged | 151.000 | 0.010 | 144.000 | 904.780 | 130288.130 | 22801.000 | Insect Damaged | 0.340 | -0.069 | 0.408 | 0.511 | 0.081 |
| 121 | Insect Damaged | 153.820 | 0.010 | 146.000 | 917.350 | 133932.380 | 23660.590 | Insect Damaged | 0.400 | -0.060 | 0.420 | 0.510 | 0.071 |
| 122 | Insect Damaged | 151.600 | 0.000 | 72.000 | 452.390 | 32572.030 | 22982.560 | Disease damaged | -1.589 | -0.631 | 0.027 | 0.095 | 0.879 |
| 123 | Insect Damaged | 152.060 | 0.010 | 127.000 | 797.960 | 101341.460 | 23122.240 | Insect Damaged | -0.180 | -0.103 | 0.296 | 0.474 | 0.229 |
| 124 | Insect Damaged | 124.060 | 0.010 | 145.000 | 911.060 | 132103.070 | 15390.880 | Insect Damaged | 0.366 | -0.105 | 0.411 | 0.513 | 0.076 |
| 125 | Insect Damaged | 137.090 | 0.010 | 144.000 | 904.780 | 130288.130 | 18793.670 | Insect Damaged | 0.338 | -0.090 | 0.407 | 0.512 | 0.081 |
| 126 | Insect Damaged | 156.000 | 0.000 | 296.000 | 1859.820 | 550507.560 | 24336.000 | Good | 4.739 | 0.597 | 0.877 | 0.123 | 0.000 |
| 127 | Insect Damaged | 126.090 | 0.010 | 141.000 | 885.930 | 124916.010 | 15898.690 | Insect Damaged | 0.245 | -0.113 | 0.388 | 0.513 | 0.099 |
| 128 | Insect Damaged | 123.110 | 0.010 | 145.000 | 911.060 | 132103.970 | 15156.070 | Insect Damaged | 0.374 | -0.118 | 0.411 | 0.514 | 0.075 |
| 129 | Insect Damaged | 123.330 | 0.010 | 144.000 | 904.780 | 130288.130 | 15210.290 | Insect Damaged | 0.336 | -0.109 | 0.405 | 0.514 | 0.081 |
| 130 | Insect Damaged | 123.090 | 0.000 | 218.000 | 1369.730 | 298602.100 | 15151.150 | Good | 2.515 | 0.161 | 0.696 | 0.303 | 0.000 |
| 131 | Insect Damaged | 109.300 | 0.010 | 180.000 | 1130.970 | 203575.200 | 11946.490 | Good | 1.407 | -0.012 | 0.565 | 0.428 | 0.007 |
| 132 | Insect Damaged | 206.400 | 0.010 | 182.000 | 1143.540 | 208124.230 | 42600.960 | Good | 1.481 | 0.155 | 0.585 | 0.409 | 0.006 |
| 133 | Insect Damaged | 157.090 | 0.010 | 182.000 | 1143.540 | 208124.230 | 24677.270 | Good | 1.472 | 0.062 | 0.578 | 0.416 | 0.006 |
| 134 | Insect Damaged | 152.060 | 0.010 | 144.000 | 904.780 | 130288.130 | 23122.240 | Insect Damaged | 0.340 | -0.068 | 0.409 | 0.511 | 0.081 |
| 135 | Insect Damaged | 109.760 | 0.000 | 215.000 | 1350.880 | 290440.240 | 12047.260 | Good | 2.427 | 0.131 | 0.686 | 0.314 | 0.001 |
| 136 | Insect Damaged | 107.630 | 0.010 | 144.000 | 904.780 | 130288.130 | 11584.220 | Insect Damaged | 0.334 | -0.128 | 0.404 | 0.515 | 0.081 |
| 137 | Insect Damaged | 114.060 | 0.010 | 141.000 | 885.930 | 124916.010 | 13009.680 | Insect Damaged | 0.244 | -0.128 | 0.387 | 0.514 | 0.099 |
| 138 | Insect Damaged | 129.640 | 0.010 | 144.000 | 904.780 | 130288.130 | 16806.530 | Insect Damaged | 0.337 | -0.101 | 0.406 | 0.513 | 0.081 |
| 139 | Insect Damaged | 109.600 | 0.010 | 180.000 | 113097 | 203575.200 | 12012.160 | Insect Damaged | 1.201 | -1.909 | 0.409 | 0.581 | 0.010 |
| 140 | Insect Damaged | 152.360 | 0.010 | 180.000 | 113097 | 203575.200 | 23213.670 | Insect Damaged | 1.207 | -1.851 | 0.413 | 0.576 | 0.010 |
| 141 | Insect Damaged | 114.070 | 0.010 | 180.000 | 113097 | 203575.200 | 13011.960 | Insect Damaged | 1.202 | -1.904 | 0.409 | 0.581 | 0.010 |
| 142 | Insect Damaged | 157.070 | 0.010 | 140.000 | 879.650 | 123150.430 | 24670.980 | Insect Damaged | 0.219 | -0.069 | 0.386 | 0.510 | 0.105 |
| 143 | Insect Damaged | 117.090 | 0.000 | 230.000 | 1445.130 | 332380.500 | 1371.070 | Good | 2.854 | 0.145 | 0.727 | 0.273 | 0.000 |
| 144 | Insect Damaged | 105.000 | 0.010 | 180.000 | 1130.970 | 203575.200 | 11025.000 | Good | 1.407 | -0.017 | 0.565 | 0.429 | 0.007 |
| 145 | Insect Damaged | 125.690 | 0.010 | 108.000 | 678.580 | 73287.070 | 15797.980 | Disease damaged | -0.780 | -0.158 | 0.145 | 0.310 | 0.544 |
| 146 | Insect Damaged | 118.920 | 0.010 | 143.000 | 898.500 | 128484.860 | 14141.970 | Insect Damaged | 0.305 | -0.117 | 0.399 | 0.514 | 0.087 |
| 147 | Insect Damaged | 144.760 | 0.010 | 144.000 | 904.780 | 130288.130 | 20955.460 | Insect Damaged | 0.339 | -0.079 | 0.408 | 0.512 | 0.081 |
| 148 | Insect Damaged | 142.090 | 0.010 | 140.000 | 879.650 | 123150.430 | 20189.570 | Insect Damaged | 0.217 | -0.093 | 0.384 | 0.511 | 0.105 |
| 149 | Insect Damaged | 127.640 | 0.010 | 180.000 | 1130.970 | 203575.200 | 16291.970 | Good | 1.409 | 0.011 | 0.567 | 0.426 | 0.007 |
| 150 | Insect Damaged | 125.820 | 0.010 | 180.000 | 1130.970 | 203575.200 | 15830.670 | Good | 1.409 | 0.008 | 0.567 | 0.427 | 0.007 |
| 151 | Insect Damaged | 101.650 | 0.010 | 182.000 | 1143.540 | 208124.230 | 10332.720 | Good | 1.466 | -0.013 | 0.572 | 0.422 | 0.006 |
| 152 | Insect Damaged | 156.390 | 0.000 | 218.000 | 1369.730 | 298602.100 | 24457.780 | Good | 2.519 | 0.210 | 0.699 | 0.300 | 0.000 |
| 153 | Insect Damaged | 131.300 | 0.010 | 144.000 | 904.780 | 130288.130 | 17239.690 | Insect Damaged | 0.337 | -0.098 | 0.406 | 0.513 | 0.081 |
| 154 | Insect Damaged | 107.900 | 0.010 | 180.000 | 1130.970 | 203575.200 | 10383.610 | Good | 1.406 | -0.020 | 0.564 | 0.429 | 0.007 |
| 155 | Insect Damaged | 101.900 | 0.000 | 216.000 | 1357.170 | 293148.290 | 18152.170 | Good | 2.459 | 0.167 | 0.691 | 0.309 | 0.001 |
| 156 | Insect Damaged | 134.730 | 0.000 | 252.000 | 1583.360 | 399007.400 | 18152.170 | Good | 3.491 | 0.337 | 0.790 | 0.210 | 0.000 |
| 157 | Insect Damaged | 160.730 | 0.010 | 146.000 | 917.350 | 133932.380 | 25834.130 | Insect Damaged | 0.401 | -0.048 | 0.421 | 0.509 | 0.071 |
| 158 | Insect Damaged | 171.620 | 0.000 | 209.000 | 1313.190 | 274455.870 | 29453.420 | Good | 2.262 | 0.196 | 0.673 | 0.326 | 0.001 |
| 159 | Insect Damaged | 134.650 | 0.010 | 161.000 | 1011.590 | 162866.450 | 18130.620 | Good | 0.848 | -0.045 | 0.491 | 0.484 | 0.026 |
| 160 | Insect Damaged | 142.020 | 0.010 | 180.000 | 1130.970 | 203575.200 | 20169.680 | Good | 1.411 | 0.031 | 0.569 | 0.425 | 0.007 |
| 161 | Insect Damaged | 163.730 | 0.010 | 121.000 | 760.270 | 91992.120 | 26807.510 | Insect Damaged | -0.364 | -0.093 | 0.251 | 0.435 | 0.314 |
| 162 | Insect Damaged | 115.000 | 0.010 | 130.000 | 816.810 | 106185.830 | 13225.000 | Insect Damaged | -0.092 | -0.149 | 0.315 | 0.491 | 0.194 |
| 163 | Insect Damaged | 149.650 | 0.010 | 143.000 | 898.500 | 128484.860 | 22395.120 | Insect Damaged | 0.309 | -0.074 | 0.403 | 0.511 | 0.086 |
| 164 | Insect Damaged | 147.150 | 0.010 | 161.000 | 1011.590 | 162866.450 | 21653.120 | Good | 0.849 | -0.027 | 0.492 | 0.482 | 0.025 |
| 165 | Insect Damaged | 180.000 | 0.010 | 160.000 | 1005.310 | 160849.540 | 32400.000 | Good | 0.825 | 0.026 | 0.492 | 0.481 | 0.027 |
| 166 | Insect Damaged | 128.110 | 0.010 | 171.000 | 1074.420 | 183726.620 | 16412.170 | Good | 1.144 | -0.021 | 0.532 | 0.455 | 0.013 |
| 167 | Insect Damaged | 119.500 | 0.010 | 131.000 | 823.100 | 107825.740 | 14280.250 | Insect Damaged | -0.061 | -0.142 | 0.323 | 0.494 | 0.183 |
| 168 | Insect Damaged | 112.040 | 0.010 | 121.000 | 760.270 | 91992.120 | 12552.960 | Insect Damaged | -0.371 | -0.166 | 0.246 | 0.437 | 0.316 |
| 169 | Insect Damaged | 137.100 | 0.010 | 125.000 | 785.400 | 98174.770 | 18796.410 | Insect Damaged | -0.244 | -0.128 | 0.280 | 0.464 | 0.257 |
| 170 | Insect Damaged | 130.460 | 0.010 | 124.000 | 779.110 | 96610.260 | 17019.810 | Insect Damaged | -0.276 | -0.139 | 0.271 | 0.458 | 0.271 |
| 171 | Insect Damaged | 123.700 | 0.010 | 135.000 | 848.230 | 114511.050 | 15301.690 | Insect Damaged | 0.062 | -0.129 | 0.350 | 0.506 | 0.144 |
| 172 | Insect Damaged | 143.600 | 0.010 | 161.000 | 1011.590 | 162866.450 | 20620.960 | Good | 0.849 | -0.032 | 0.492 | 0.483 | 0.025 |
| 173 | Insect Damaged | 150.460 | 0.010 | 151.000 | 948.760 | 143262.910 | 22638.210 | Insect Damaged | 0.551 | -0.052 | 0.446 | 0.504 | 0.051 |
| 174 | Insect Damaged | 134.210 | 0.010 | 125.000 | 785.400 | 98177.770 | 18012.320 | Insect Damaged | -0.245 | -0.132 | 0.279 | 0.464 | 0.257 |
| 175 | Insect Damaged | 176.800 | 0.010 | 146.000 | 917.340 | 133932.380 | 31258.240 | Insect Damaged | 0.404 | -0.020 | 0.423 | 0.507 | 0.070 |
| 176 | Insect Damaged | 134.220 | 0.010 | 163.000 | 1024.160 | 166937.950 | 18015.010 | Good | 0.907 | -0.040 | 0.499 | 0.478 | 0.022 |
| 177 | Insect Damaged | 179.020 | 0.010 | 136.000 | 854.510 | 116213.790 | 32048.160 | Insect Damaged | 0.101 | -0.040 | 0.363 | 0.503 | 0.134 |
| 178 | Insect Damaged | 167.300 | 0.010 | 159.000 | 999.030 | 158845.210 | 27989.290 | Good | 0.793 | 0.000 | 0.486 | 0.485 | 0.029 |
| 179 | Insect Damaged | 159.410 | 0.010 | 147.000 | 923.630 | 135773.350 | 25411.550 | Insect Damaged | 0.431 | -0.048 | 0.426 | 0.508 | 0.066 |
| 180 | Insect Damaged | 170.470 | 0.010 | 126.000 | 791.680 | 99751.850 | 29060.020 | Insect Damaged | -0.209 | -0.073 | 0.291 | 0.467 | 0.242 |
| 181 | Insect Damaged | 125.700 | 0.010 | 160.000 | 1005.310 | 160849.540 | 15800.490 | Insect Damaged | 0.817 | -0.061 | 0.485 | 0.487 | 0.027 |
| 182 | Insect Damaged | 112.040 | 0.010 | 132.000 | 829.380 | 109478.220 | 12552.960 | Insect Damaged | -0.031 | -0.149 | 0.329 | 0.498 | 0.173 |
| 183 | Insect Damaged | 119.800 | 0.010 | 147.000 | 923.630 | 135773.350 | 14352.040 | Insect Damaged | 0.426 | -0.106 | 0.421 | 0.512 | 0.067 |
| 184 | Insect Damaged | 176.500 | 0.010 | 160.000 | 1005.310 | 160849.540 | 31152.250 | Good | 0.824 | 0.020 | 0.492 | 0.481 | 0.027 |
| 185 | Insect Damaged | 167.390 | 0.010 | 169.000 | 1061.860 | 179454.060 | 28019.410 | Good | 1.090 | 0.033 | 0.529 | 0.457 | 0.014 |
| 186 | Insect Damaged | 134.490 | 0.010 | 146.000 | 917.350 | 133932.380 | 18087.560 | Insect Damaged | 0.398 | -0.089 | 0.418 | 0.512 | 0.071 |
| 187 | Insect Damaged | 125.800 | 0.010 | 150.000 | 942.480 | 141371.670 | 15825.640 | Insect Damaged | 0.517 | -0.090 | 0.438 | 0.508 | 0.054 |
| 188 | Insect Damaged | 156.390 | 0.010 | 153.000 | 961.330 | 147083.080 | 24457.830 | Insect Damaged | 0.611 | -0.036 | 0.456 | 0.500 | 0.044 |
| 189 | Insect Damaged | 125.820 | 0.010 | 144.000 | 904.780 | 130288.130 | 15830.670 | Insect Damaged | 0.336 | -0.106 | 0.406 | 0.513 | 0.081 |
| 190 | Insect Damaged | 144.760 | 0.010 | 130.000 | 816.810 | 106185.830 | 20955.460 | Insect Damaged | -0.089 | -0.109 | 0.318 | 0.489 | 0.194 |
| 191 | Insect Damaged | 157.070 | 0.010 | 159.000 | 999.030 | 158845.210 | 24670.980 | Insect Damaged | 0.791 | -0.017 | 0.484 | 0.486 | 0.029 |
| 192 | Insect Damaged | 144.760 | 0.010 | 166.000 | 1043.010 | 173139.450 | 20955.460 | Good | 0.998 | -0.015 | 0.513 | 0.469 | 0.018 |
| 193 | Insect Damaged | 142.090 | 0.010 | 128.000 | 804.250 | 102943.710 | 20189.570 | Insect Damaged | -0.151 | -0.116 | 0.303 | 0.480 | 0.217 |
| 194 | Insect Damaged | 109.760 | 0.010 | 132.000 | 829.380 | 109478.220 | 12047.260 | Insect Damaged | -0.032 | -0.152 | 0.329 | 0.498 | 0.173 |
| 195 | Insect Damaged | 152.120 | 0.010 | 156.000 | 980.180 | 152907.600 | 23140.490 | Insect Damaged | 0.701 | -0.034 | 0.470 | 0.494 | 0.036 |
| 196 | Insect Damaged | 109.360 | 0.010 | 147.000 | 923.630 | 135773.350 | 11959.610 | Insect Damaged | 0.425 | -0.118 | 0.420 | 0.513 | 0.067 |
| 197 | Insect Damaged | 125.090 | 0.010 | 132.000 | 829.380 | 109478.220 | 15647.510 | Insect Damaged | -0.030 | -0.133 | 0.330 | 0.497 | 0.173 |
| 198 | Insect Damaged | 118.920 | 0.010 | 159.000 | 999.030 | 158845.210 | 11141.970 | Insect Damaged | 0.785 | -0.088 | 0.479 | 0.492 | 0.030 |
| 199 | Insect Damaged | 144.040 | 0.010 | 141.000 | 885.930 | 124916.010 | 20747.520 | Insect Damaged | 0.247 | -0.087 | 0.390 | 0.512 | 0.098 |
| 200 | Insect Damaged | 139.920 | 0.010 | 160.000 | 1005.310 | 160849.540 | 19577.610 | Good | 0.819 | -0.041 | 0.487 | 0.486 | 0.027 |
| 201 | Disease damaged | 109.110 | 0.010 | 76.000 | 477.500 | 36291.680 | 11904.990 | Disease damaged | -1.853 | -0.108 | 0.015 | 0.052 | 0.933 |
| 202 | Disease damaged | 139.010 | 0.010 | 96.000 | 603.200 | 57907.200 | 19323.780 | Disease damaged | -1.168 | -0.132 | 0.072 | 0.183 | 0.745 |
| 203 | Disease damaged | 150.900 | 0.010 | 92.000 | 578.100 | 53185.200 | 22770.810 | Disease damaged | -1.298 | -0.107 | 0.055 | 0.147 | 0.798 |
| 204 | Disease damaged | 129.810 | 0.010 | 84.000 | 527.800 | 44335.200 | 16850.640 | Disease damaged | -1.571 | -0.117 | 0.030 | 0.090 | 0.880 |
| 205 | Disease damaged | 140.220 | 0.010 | 110.000 | 691.200 | 76032.000 | 19661.650 | Disease damaged | -0.715 | -0.137 | 0.161 | 0.332 | 0.507 |
| 206 | Disease damaged | 103.550 | 0.010 | 72.000 | 452.400 | 32572.800 | 10722.600 | Disease damaged | -1.998 | -0.090 | 0.011 | 0.038 | 0.951 |
| 207 | Disease damaged | 128.600 | 0.010 | 114.000 | 716.300 | 81658.200 | 16537.960 | Disease damaged | -0.589 | -0.152 | 0.191 | 0.374 | 0.435 |
| 208 | Disease damaged | 155.060 | 0.010 | 86.000 | 540.400 | 46474.400 | 24043.600 | Disease damaged | -1.500 | -0.085 | 0.035 | 0.102 | 0.863 |
| 209 | Disease damaged | 137.390 | 0.010 | 111.000 | 697.400 | 77411.400 | 18876.010 | Disease damaged | -0.683 | -0.141 | 0.169 | 0.343 | 0.489 |
| 210 | Disease damaged | 158.120 | 0.010 | 99.000 | 622.000 | 61578.000 | 25001.930 | Disease damaged | -1.067 | -0.105 | 0.089 | 0.212 | 0.699 |
| 211 | Disease damaged | 106.490 | 0.010 | 85.000 | 534.100 | 45398.500 | 1134.120 | Disease damaged | -1.545 | -0.202 | 0.031 | 0.096 | 0.873 |
| 212 | Disease damaged | 120.060 | 0.010 | 100.000 | 628.300 | 62830.000 | 14414.400 | Disease damaged | -1.039 | -0.162 | 0.093 | 0.223 | 0.684 |
| 213 | Disease damaged | 148.130 | 0.010 | 96.000 | 603.200 | 57907.200 | 21942.500 | Disease damaged | -1.166 | -0.118 | 0.073 | 0.183 | 0.745 |
| 214 | Disease damaged | 139.430 | 0.010 | 84.000 | 527.800 | 44335.200 | 19440.720 | Disease damaged | -1.570 | -0.103 | 0.030 | 0.090 | 0.880 |
| 215 | Disease damaged | 143.670 | 0.010 | 96.000 | 603.200 | 57907.200 | 20641.070 | Disease damaged | -1.167 | -0.125 | 0.072 | 0.183 | 0.745 |
| 216 | Disease damaged | 161.320 | 0.010 | 85.000 | 534.100 | 45398.500 | 26024.140 | Disease damaged | -1.533 | -0.072 | 0.033 | 0.096 | 0.871 |
| 217 | Disease damaged | 116.020 | 0.010 | 85.000 | 534.100 | 45398.500 | 13460.640 | Disease damaged | -1.539 | -0.138 | 0.032 | 0.096 | 0.872 |
| 218 | Disease damaged | 114.210 | 0.010 | 98.000 | 615.800 | 45398.500 | 13043.920 | Disease damaged | -1.099 | -0.173 | 0.082 | 0.204 | 0.713 |
| 219 | Disease damaged | 113.350 | 0.010 | 76.000 | 477.500 | 36290.000 | 12848.220 | Disease damaged | -1.853 | -0.103 | 0.015 | 0.052 | 0.933 |
| 220 | Disease damaged | 126.620 | 0.010 | 10.000 | 62.800 | 628.000 | 16032.620 | Disease damaged | -3.623 | -0.606 | 0.000 | 0.001 | 0.999 |
| 221 | Disease damaged | 157.760 | 0.010 | 86.000 | 540.400 | 46474.400 | 24888.220 | Disease damaged | -1.499 | -0.081 | 0.035 | 0.102 | 0.863 |
| 222 | Disease damaged | 113.360 | 0.100 | 100.000 | 628.300 | 628300.000 | 12850.490 | Disease damaged | -4.869 | 5.484 | 0.000 | 0.000 | 1.000 |
| 223 | Disease damaged | 149.610 | 0.010 | 7.000 | 44.000 | 308.000 | 22383.150 | Disease damaged | -3.880 | -0.327 | 0.000 | 0.001 | 0.999 |
| 224 | Disease damaged | 158.720 | 0.010 | 104.000 | 653.500 | 67964.000 | 25192.040 | Disease damaged | -0.904 | -0.108 | 0.119 | 0.265 | 0.615 |
| 225 | Disease damaged | 126.080 | 0.010 | 87.000 | 546.600 | 47554.200 | 15896.170 | Disease damaged | -1.469 | -0.131 | 0.038 | 0.109 | 0.854 |
| 226 | Disease damaged | 116.910 | 0.010 | 106.000 | 666.000 | 70596.000 | 13667.950 | Disease damaged | -0.845 | -0.169 | 0.131 | 0.288 | 0.581 |
| 227 | Disease damaged | 115.620 | 0.010 | 76.000 | 477.500 | 36290.000 | 13367.980 | Disease damaged | -1.853 | -0.100 | 0.015 | 0.052 | 0.933 |
| 228 | Disease damaged | 153015 | 0.010 | 84.000 | 527.800 | 0.000 | 23454.920 | Disease damaged | -2.628 | 3.069 | 0.003 | 0.006 | 0.991 |
| 229 | Disease damaged | 111.110 | 0.010 | 79.000 | 496.400 | 39215.600 | 12345.430 | Disease damaged | -1.747 | -0.120 | 0.020 | 0.064 | 0.917 |
| 230 | Disease damaged | 136.370 | 0.010 | 83.000 | 521.500 | 43284.500 | 18596.780 | Disease damaged | -1.605 | -0.104 | 0.027 | 0.084 | 0.889 |
| 231 | Disease damaged | 161.070 | 0.010 | 96.000 | 603.190 | 57905.840 | 25943.540 | Disease damaged | -1.164 | -0.097 | 0.073 | 0.183 | 0.744 |
| 232 | Disease damaged | 133.890 | 0.010 | 100.000 | 628.820 | 62831.880 | 17926.530 | Disease damaged | -1.037 | -0.143 | 0.093 | 0.223 | 0.684 |
| 233 | Disease damaged | 148.600 | 0.010 | 86.000 | 540.350 | 46470.440 | 22081.960 | Disease damaged | -1.501 | -0.096 | 0.035 | 0.102 | 0.863 |
| 234 | Disease damaged | 130.560 | 0.010 | 94.000 | 590.620 | 55518.230 | 17048.910 | Disease damaged | -1.235 | -0.141 | 0.063 | 0.164 | 0.773 |
| 235 | Disease damaged | 169.980 | 0.010 | 77.000 | 483.810 | 37253.060 | 26237.520 | Disease damaged | -1.811 | -0.038 | 0.017 | 0.055 | 0.928 |
| 236 | Disease damaged | 115.300 | 0.010 | 86.000 | 540.350 | 46470.400 | 13291.780 | Disease damaged | -1.505 | -0.142 | 0.035 | 0.102 | 0.863 |
| 237 | Disease damaged | 150.060 | 0.100 | 104.000 | 653.450 | 67958.930 | 22518.000 | Disease damaged | -4.397 | 5.127 | 0.000 | 0.000 | 1.000 |
| 238 | Disease damaged | 144.150 | 0.010 | 75.000 | 471.240 | 35342.920 | 20779.220 | Disease damaged | -1.885 | -0.056 | 0.014 | 0.048 | 0.938 |
| 239 | Disease damaged | 120.450 | 0.010 | 99.000 | 622.040 | 61581.500 | 14508.200 | Disease damaged | -1.071 | -0.161 | 0.087 | 0.213 | 0.700 |
| 240 | Disease damaged | 131.210 | 0.010 | 114.000 | 716.280 | 81656.280 | 17216.060 | Disease damaged | -0.589 | -0.148 | 0.192 | 0.374 | 0.434 |
| 241 | Disease damaged | 167.860 | 0.010 | 104.000 | 653.450 | 67958.930 | 28176.980 | Disease damaged | -0.903 | -0.092 | 0.120 | 0.265 | 0.615 |
| 242 | Disease damaged | 127.600 | 0.010 | 96.000 | 603.190 | 57905.840 | 16281.760 | Disease damaged | -1.169 | -0.148 | 0.072 | 0.183 | 0.745 |
| 243 | Disease damaged | 140.000 | 0.010 | 85.000 | 534.070 | 45396.010 | 19600.000 | Disease damaged | -1.536 | -0.106 | 0.032 | 0.096 | 0.872 |
| 244 | Disease damaged | 117.390 | 0.010 | 74.000 | 464.960 | 34406.730 | 13780.410 | Disease damaged | -1.924 | -0.087 | 0.013 | 0.045 | 0.943 |
| 245 | Disease damaged | 137.770 | 0.010 | 92.000 | 578.050 | 53180.880 | 18980.570 | Disease damaged | -1.300 | -0.127 | 0.055 | 0.147 | 0.798 |
| 246 | Disease damaged | 156.330 | 0.010 | 110.000 | 691.150 | 76026.540 | 24439.070 | Disease damaged | -0.712 | -0.112 | 0.162 | 0.331 | 0.506 |
| 247 | Disease damaged | 100.130 | 0.010 | 81.000 | 508.940 | 41223.980 | 10026.020 | Disease damaged | -1.678 | -0.141 | 0.023 | 0.073 | 0.904 |
| 248 | Disease damaged | 120.950 | 0.010 | 81.000 | 508.940 | 41223.980 | 14628.900 | Disease damaged | -1.668 | -0.130 | 0.024 | 0.075 | 0.902 |
| 249 | Disease damaged | 152.910 | 0.010 | 77.000 | 483.810 | 37253.060 | 23381.470 | Disease damaged | -1.812 | -0.053 | 0.017 | 0.055 | 0.928 |
| 250 | Disease damaged | 129.620 | 0.010 | 96.000 | 603.190 | 57905.840 | 22159.300 | Disease damaged | -1.166 | -0.117 | 0.073 | 0.183 | 0.745 |
| 251 | Disease damaged | 148.860 | 0.010 | 98.000 | 615.750 | 60343.710 | 13169.860 | Disease damaged | -1.105 | -0.166 | 0.082 | 0.202 | 0.716 |
| 252 | Disease damaged | 114.760 | 0.090 | 111.000 | 69.710 | 77415.130 | 13169.860 | Disease damaged | -3.955 | 4.755 | 0.000 | 0.000 | 1.000 |
| 253 | Disease damaged | 136.920 | 0.010 | 85.000 | 534.070 | 45396.010 | 18747.090 | Disease damaged | -1.536 | -0.110 | 0.032 | 0.096 | 0.872 |
| 254 | Disease damaged | 125.710 | 0.010 | 77.000 | 483.810 | 37253.060 | 15803.000 | Disease damaged | -1.816 | -0.092 | 0.017 | 0.055 | 0.928 |
| 255 | Disease damaged | 156.100 | 0.010 | 90.000 | 565.490 | 33483.010 | 24367.210 | Disease damaged | -1.358 | -0.101 | 0.048 | 0.132 | 0.819 |
| 256 | Disease damaged | 146.670 | 0.010 | 104.000 | 653.450 | 67958.930 | 21512.090 | Disease damaged | -0.906 | -0.127 | 0.119 | 0.266 | 0.616 |
| 257 | Disease damaged | 116.450 | 0.010 | 87.000 | 546.640 | 47557.430 | 13560.600 | Disease damaged | -1.470 | -0.144 | 0.037 | 0.109 | 0.854 |
| 258 | Disease damaged | 131.710 | 0.010 | 93.000 | 584.340 | 54343.270 | 17347.520 | Disease damaged | -1.267 | -0.138 | 0.059 | 0.155 | 0.786 |
| 259 | Disease damaged | 149.860 | 0.010 | 80.000 | 502.650 | 40212.390 | 22458.020 | Disease damaged | -1.707 | -0.072 | 0.022 | 0.069 | 0.910 |
| 260 | Disease damaged | 136.750 | 0.010 | 90.000 | 565.490 | 33483.090 | 18700.560 | Disease damaged | -1.356 | -0.137 | 0.048 | 0.133 | 0.818 |
| 261 | Disease damaged | 124.110 | 0.010 | 68.000 | 427.260 | 29053.450 | 15403.290 | Disease damaged | -2.143 | -0.036 | 0.007 | 0.028 | 0.965 |
| 262 | Disease damaged | 104.010 | 0.010 | 85.000 | 534.070 | 45396.010 | 10818.080 | Disease damaged | -1.540 | -0.152 | 0.032 | 0.096 | 0.872 |
| 263 | Disease damaged | 156.000 | 0.010 | 87.000 | 546.640 | 47557.430 | 24336.000 | Disease damaged | -1.465 | -0.087 | 0.038 | 0.109 | 0.853 |
| 264 | Disease damaged | 135.060 | 0.010 | 83.000 | 521.500 | 43284.860 | 18241.200 | Disease damaged | -1.605 | -0.106 | 0.027 | 0.084 | 0.889 |
| 265 | Disease damaged | 124.860 | 0.010 | 75.000 | 471.240 | 0.000 | 15590.020 | Disease damaged | -1.875 | -0.096 | 0.014 | 0.049 | 0.936 |
| 266 | Disease damaged | 149.150 | 0.010 | 105.000 | 659.730 | 69272.120 | 22245.720 | Disease damaged | -0.873 | -0.124 | 0.126 | 0.277 | 0.598 |
| 267 | Disease damaged | 113.980 | 0.010 | 96.000 | 603.190 | 57905.840 | 12991.440 | Disease damaged | -1.170 | -0.165 | 0.072 | 0.183 | 0.746 |
| 268 | Disease damaged | 148.730 | 0.010 | 86.000 | 540.350 | 46470.440 | 22135.490 | Disease damaged | -1.501 | -0.095 | 0.035 | 0.102 | 0.863 |
| 269 | Disease damaged | 138.210 | 0.010 | 99.000 | 622.040 | 61581.800 | 19102.000 | Disease damaged | -1.069 | -0.137 | 0.088 | 0.212 | 0.700 |
| 270 | Disease damaged | 149.670 | 0.010 | 104.000 | 653.450 | 67958.930 | 22401.110 | Disease damaged | -0.906 | -0.122 | 0.119 | 0.266 | 0.616 |
| 271 | Disease damaged | 156.710 | 0.010 | 91.000 | 571.770 | 52031.060 | 24558.020 | Disease damaged | -1.331 | -0.096 | 0.051 | 0.139 | 0.810 |
| 272 | Disease damaged | 163.000 | 0.010 | 86.000 | 540.350 | 46470.440 | 26569.000 | Disease damaged | -1.499 | -0.072 | 0.035 | 0.102 | 0.863 |
| 273 | Disease damaged | 114.670 | 0.010 | 90.000 | 565.490 | 50893.800 | 13149.210 | Disease damaged | -1.370 | -0.153 | 0.047 | 0.131 | 0.822 |
| 274 | Disease damaged | 146.450 | 0.010 | 100.000 | 628.320 | 62831.850 | 21447.600 | Disease damaged | -1.035 | -0.125 | 0.094 | 0.223 | 0.683 |
| 275 | Disease damaged | 123.860 | 0.010 | 91.000 | 571.770 | 52031.060 | 15341.300 | Disease damaged | -1.335 | -0.144 | 0.051 | 0.139 | 0.811 |
| 276 | Disease damaged | 150.750 | 0.010 | 75.000 | 471.240 | 0.000 | 22725.560 | Disease damaged | -1.871 | -0.058 | 0.014 | 0.049 | 0.936 |
| 277 | Disease damaged | 100.920 | 0.010 | 86.000 | 540.350 | 4647044.000 | 10184.850 | Disease damaged | -3.138 | 1.457 | 0.001 | 0.003 | 0.997 |
| 278 | Disease damaged | 145.980 | 0.010 | 73.000 | 458.670 | 33483.090 | 21310.160 | Disease damaged | -1.957 | -0.041 | 0.012 | 0.041 | 0.947 |
| 279 | Disease damaged | 139.420 | 0.010 | 90.000 | 565.490 | 50893.800 | 19437.940 | Disease damaged | -1.367 | -0.120 | 0.047 | 0.131 | 0.822 |
| 280 | Disease damaged | 114.310 | 0.010 | 106.000 | 666.020 | 70597.870 | 13066.780 | Disease damaged | -0.845 | -0.172 | 0.131 | 0.288 | 0.581 |
| 281 | Disease damaged | 161.610 | 0.010 | 91.000 | 571.770 | 52031.060 | 26117.790 | Disease damaged | -1.330 | -0.088 | 0.052 | 0.139 | 0.810 |
| 282 | Disease damaged | 123.890 | 0.010 | 83.000 | 521.500 | 43284.860 | 15348.730 | Disease damaged | -1.607 | -0.121 | 0.027 | 0.084 | 0.889 |
| 283 | Disease damaged | 145.420 | 0.010 | 77.000 | 483.810 | 37253.010 | 21146.980 | Disease damaged | -1.813 | -0.064 | 0.017 | 0.055 | 0.928 |
| 284 | Disease damaged | 136.140 | 0.010 | 106.000 | 666.020 | 70597.870 | 18534.100 | Disease damaged | -0.843 | -0.143 | 0.132 | 0.288 | 0.580 |
| 285 | Disease damaged | 129.580 | 0.010 | 96.000 | 603.190 | 57905.840 | 16790.980 | Disease damaged | -1.169 | -0.145 | 0.072 | 0.183 | 0.745 |
| 286 | Disease damaged | 152.060 | 0.010 | 87.000 | 546.640 | 47557.430 | 23122.240 | Disease damaged | -1.466 | -0.094 | 0.038 | 0.109 | 0.853 |
| 287 | Disease damaged | 163.480 | 0.010 | 96.000 | 603.190 | 57905.840 | 26725.710 | Disease damaged | -1.164 | -0.093 | 0.073 | 0.183 | 0.744 |
| 288 | Disease damaged | 130.110 | 0.010 | 70.000 | 439182 | 30787.610 | 16928.610 | Disease damaged | -2.875 | -7.480 | 0.001 | 0.022 | 0.977 |
| 289 | Disease damaged | 138.590 | 0.010 | 83.000 | 521.500 | 43284.430 | 19207.190 | Disease damaged | -1.605 | -0.101 | 0.028 | 0.084 | 0.888 |
| 290 | Disease damaged | 139.710 | 0.010 | 81.000 | 508.940 | 41223.980 | 19518.880 | Disease damaged | -1.674 | -0.091 | 0.023 | 0.073 | 0.903 |
| 291 | Disease damaged | 153.210 | 0.010 | 91.000 | 571.770 | 52031.060 | 23473.300 | Disease damaged | -1.331 | -0.101 | 0.051 | 0.139 | 0.810 |
| 292 | Disease damaged | 130.060 | 0.010 | 87.000 | 546.640 | 47557.430 | 16915.600 | Disease damaged | -1.469 | -0.126 | 0.038 | 0.109 | 0.854 |
| 293 | Disease damaged | 147.340 | 0.010 | 96.000 | 605.190 | 57905.840 | 21709.080 | Disease damaged | -1.166 | -0.119 | 0.073 | 0.183 | 0.745 |
| 294 | Disease damaged | 103.400 | 0.010 | 73.000 | 458.670 | 33485.090 | 10755.760 | Disease damaged | -1.961 | -0.096 | 0.012 | 0.041 | 0.947 |
| 295 | Disease damaged | 144.020 | 0.010 | 86.000 | 540.550 | 4647044.000 | 20741.760 | Disease damaged | -3.133 | 1.513 | 0.001 | 0.003 | 0.997 |
| 296 | Disease damaged | 135.590 | 0.010 | 96.000 | 603.190 | 57905.840 | 18584.650 | Disease damaged | -1.168 | -0.135 | 0.072 | 0.183 | 0.745 |
| 297 | Disease damaged | 118.110 | 0.010 | 75.000 | 471.240 | 35342.920 | 13949.970 | Disease damaged | -1.888 | -0.092 | 0.014 | 0.048 | 0.938 |
| 298 | Disease damaged | 128.840 | 0.010 | 85.000 | 534.070 | 45516.010 | 16599.750 | Disease damaged | -1.537 | -0.122 | 0.032 | 0.096 | 0.872 |
| 299 | Disease damaged | 123.550 | 0.010 | 76.000 | 477.520 | 36291.680 | 15264.600 | Disease damaged | -1.851 | -0.091 | 0.015 | 0.052 | 0.933 |
| 300 | Disease damaged | 157.070 | 0.010 | 68.000 | 427.260 | 29053.450 | 24670.980 | Disease damaged | -2.139 | 0.012 | 0.007 | 0.028 | 0.964 |

A = Amplitude, F = Frequency, I = Intensity, T = Period, V = Velocity, ʎ = Wavelength,

‘Pred. Yam quality’ is the predicted yam quality class.

Quality class: 1= Good, 2 = Insect damaged and 3 = Diseased damaged

‘DSF1’ is ‘Discriminant Scores from Function (Model) 1’.

‘DSF2’ is ‘Discriminant Scores from Function (Model) 2’,

‘PMC1’ is the ‘Probabilities of Membership in Yam Quality Class 1’.

‘PMC2’ is the ‘Probabilities of Membership in Yam Quality Class 2’.

‘PMC3’ is the ‘Probabilities of Membership in Yam Quality Class 3’

Table S9: Test for equality of variance, group means and covariance matrices of yellow yam quality for two acoustic techniques

| Tests of Equality of Group Means | | | | | | | | | | | | | | | | | | | | | | | |  |  |
| --- | --- | --- | --- | --- | --- | --- | --- | --- | --- | --- | --- | --- | --- | --- | --- | --- | --- | --- | --- | --- | --- | --- | --- | --- | --- |
| Acoustic Property | Software Sound generation technique | | | | | | | | | | Surface impact sound generation technique | | | | | | | | | | | | |  |  |
|  | Wilks' Lambda | F | df1 | | df2 | | | Sig. | | | | Wilks' Lambda | F | | df1 | df2 | | | | Sig. | | | |  |  |
| Amplitude | 0.996 | 0.622 | 2 | | 297 | | | 0.538 | | | | 0.994 | 0.884 | | 2 | 297 | | | | 0.414 | | | |  |  |
| Frequency | 0.611 | 94.378 | 2 | | 297 | | | 0.000 | | | | 0.844 | 27.508 | | 2 | 297 | | | | 1.10E-11 | | | |  |  |
| Intensity | 0.629 | 87.471 | 2 | | 297 | | | 0.000 | | | | 0.470 | 167.569 | | 2 | 297 | | | | 1.92E-49 | | | |  |  |
| Period | 0.849 | 26.447 | 2 | | 297 | | | 0.000 | | | | 0.995 | 0.701 | | 2 | 297 | | | | 0.497 | | | |  |  |
| Velocity | 1.000 | 0.000 | 2 | | 297 | | | 1.000 | | | | 0.999 | 0.160 | | 2 | 297 | | | | 0.852 | | | |  |  |
| Wavelength | 0.977 | 3.554 | 2 | | 297 | | | 0.030 | | | | 0.990 | 1.466 | | 2 | 297 | | | | 0.232 | | | |  |  |
| *a. Cannot be computed because this variable is a constant.* | | | | | | | | | | | | | | | | | | | | | | | |  |  |
| Box's Test of Equality of Covariance Matrices | | | | | | | | | | | | | | | | | | | | | | | |  |  |
| Log Determinants | | | | | | | | | | | | | | | | | | | | | | | |  |  |
| Yam quality | Rank | Log Determinant | |  | | |  | | |  | | Rank | | Log Determinant | | | |  | | | |  |  | |  |
| Good | 5 | 90.789 | |  | | |  | | |  | | 6 | | 60.103 | | | |  | | | |  |  | |  |
| Disease damaged | 5 | 86.235 | |  | | |  | | |  | | 6 | | 54.137 | | | |  | | | |  |  | |  |
| Insect Damaged | 5 | 86.494 | |  | | |  | | |  | | 6 | | 81.392 | | | |  | | | |  |  | |  |
| Pooled within-groups | 5 | 99.372 | |  | | |  | | |  | | 6 | | 85.272 | | | |  | | | |  |  | |  |
| *The ranks and natural logarithms of determinants printed are those of the group covariance matrices.* | | | | | | | | | | | | | | | | | | | | | | | |  |  |
| Box's Test Results | | | | | | | | | | | | | | | | | | | | | | | |  |  |
| Box's M | | 3425.295 | | | |  | | |  | | 5958.295 | | | | | |  | |  | |  | | |  | |
| F | Approx. | 111.430 | |  | |  | | |  | | 137.793 | | | | | |  | |  | |  | | |  | |
|  | df1 | 30.000 | |  | |  | | |  | | 42 | | | | | |  | |  | |  | | |  | |
|  | df2 | 279508.225 | |  | |  | | |  | | 261873.843 | | | | | |  | |  | |  | | |  | |
|  | Sig. | 0.000 | |  | |  | | |  | | 0.000 | | | | | |  | |  | |  | | |  | |
| *Tests null hypothesis of equal population covariance matrices.* | | | | | | | | | | | | | | | | | | | | | | | |  |  |

Table S10: Summary of canonical discriminant score functions of yellow yam for both acoustic techniques

| Software Sound generation technique | | | | | Surface impact sound generation technique | | | |
| --- | --- | --- | --- | --- | --- | --- | --- | --- |
| Eigen values | | | | | | | | |
| Function (DSF) | Eigen value | % of Variance | Cumulative % | Canonical Correlation | Eigen value | % of Variance | Cumulative % | Canonical Correlation |
| 1 | 1.134^a^ | 82.2 | 82.2 | 0.73 | 1.320^a^ | 98.969 | 98.969 | 0.754 |
| 2 | 0.246^a^ | 17.8 | 100.0 | 0.44 | 0.014^a^ | 1.031 | 100.000 | 0.116 |
| *a. First 2 canonical discriminant functions were used in the analysis.* | | | | | | | | |
| Wilks' Lambda | | | | | | | | |
| Test of Function(s) (DSF) | Wilks' Lambda | Chi-square | df | Sig. | Wilks' Lambda | Chi-square | df | Sig. |
| 1 through 2 | 0.38 | 288.421 | 10 | 4.35E-56 | 0.425 | 251.814 | 12.000 | 5.72E-47 |
| 2 | 0.80 | 64.833 | 4 | 2.79E-13 | 0.986 | 4.023 | 5.000 | 0.546 |
| Standardized Canonical Discriminant Function Coefficients | | | | | | | | |
| Acoustic Property | Function (DSF) | |  |  | Function (DSF) | |  |  |
|  | 1 | 2 |  |  | 1 | 2 |  |  |
| Amplitude | -0.013 | -0.107 |  |  | -0.062 | 0.184 |  |  |
| Frequency | 0.701 | 0.646 |  |  | 0.938 | 0.158 |  |  |
| Velocity | -0.662 | 0.655 |  |  | -0.135 | 0.134 |  |  |
| Wavelength | 0.247 | -0.008 |  |  | -0.052 | -0.478 |  |  |
| Intensity | 0.047 | -0.292 |  |  | 0.063 | 0.658 |  |  |
| period |  |  |  |  | -0.340 | 0.512 |  |  |
| Structure Matrix | | | | | | | | |
| Acoustic Property | Function (DSF) | |  |  | Function (DSF) | |  |  |
|  | 1 | 2 |  |  | 1 | 2 |  |  |
| Wavelength | 0.396^*^ |  |  |  | -0.034 | -0.481^*^ |  |  |
| Velocity | -0.642 | 0.999^*^ |  |  | 0.023 | 0.169^*^ |  |  |
| Frequency | 0.676 | 0.078 |  |  | 0.925^*^ | 0.149 |  |  |
| Intensity | 0.059 |  |  |  | 0.055 | 0.656^*^ |  |  |
| Period |  |  |  |  | -0.371 | 0.507^*^ |  |  |
| Amplitude | -0.042 |  |  |  | -0.065 | 0.178^*^ |  |  |
| *Pooled within-groups correlations between discriminating variables and standardized canonical discriminant functions   Variables ordered by absolute size of correlation within function.* | | | | | | | | |
| **. Largest absolute correlation between each variable and any discriminant function* | | | | | | | | |
| Discriminant Score Function (DSF) Equations | | | | | | | | |
| DSF1 = - 6.038E-07 A + 5.227E-02 F - 8.996E-06 V + 1.584E-06 W + 3.962E-08 I - 8.222 | | | | | DSF1 = - 7.034E-06 A - 4.033E+01 T + 2.898E-02 F - 1.838E-06 W - 3.546E-07 V + 4.931E-07 I - 3.517 | | | |
| DSF2 = -5.045E-06 A + 4.817E-02 F + 8.912E-06 V - 4.866E-08 W - 2.484E-07 I - 8.093 | | | | | DSF2 = 2.072E-05 A + 6.062E+01 T + 4.890E-03 F - 1.695E-05 W + 3.510E-07 V + 5.146E-06 I - 1.345E | | | |
| *A = Amplitude, F = Frequency, I = Intensity, T = Period, W = Wavelength* | | | | | |  |  |  |
